# Integrating variant functional annotation scores have varied abilities to improve power of genome-wide association studies

**DOI:** 10.1038/s41598-022-14924-1

**Published:** 2022-06-24

**Authors:** Jianhui Gao, Osvaldo Espin-Garcia, Andrew D. Paterson, Lei Sun

**Affiliations:** 1 Division of Biostatistics, Dalla Lana School of Public Health, University of Toronto, Toronto, Canada; 2 Department of Biostatistics, Princess Margaret Cancer Centre, University Health Network, Toronto, Canada; 3 Program in Genetics and Genome Biology, The Hospital for Sick Children, Toronto, Canada; 4 Department of Statistical Sciences, Faculty of Arts and Science, University of Toronto, Toronto, Canada

**Keywords:** Computational biology and bioinformatics, Genetics

## Abstract

Functional annotations have the potential to increase power of genome-wide association studies (GWAS) by prioritizing variants according to their biological function, but this potential has not been well studied. We comprehensively evaluated all 1132 traits in the UK Biobank whose SNP-heritability estimates were given “medium” or “high” labels by Neale’s lab. For each trait, we integrated GWAS summary statistics of close to 8 million common variants (minor allele frequency $>1\%$) with either their 75 individual functional scores or their meta-scores, using three different data-integration methods. Overall, the number of new genome-wide significant findings after data-integration increases as a trait SNP-heritability estimate increases. However, there is a trade-off between new findings and loss of baseline GWAS findings, resulting in similar total numbers of significant findings between using GWAS alone and integrating GWAS with functional scores, across all 1132 traits analyzed and all three data-integration methods considered. Our findings suggest that, even with the current biobank-level sample size, more informative functional scores and/or new data-integration methods are needed to further improve the power of GWAS of common variants. For example, studying variants in coding sequence and obtaining cell-type-specific scores are potential future directions.

## Introduction

In the last decade, genome-wide association studies (GWAS) have enabled the discovery and identification of thousands of genetic loci across a wide range of phenotypes^1^. However, despite their increasingly large sample sizes (e.g. $n>100{,}000$) there is a need to improve the often modest power of GWAS, as effect sizes of causal variants are believed to be small for most complex human traits^2^.

The standard GWAS approaches are designed for discovering common variants with relatively large effects (i.e. low polygenicity), and so they are not optimized for analyzing the large number of small effects in highly polygenic traits^3^. To increase the power of GWAS, earlier work have leveraged linkage results^4,5^ or summary statistics from independent GWAS of the same or related traits^6–9^. To integrate information across sources, meta-analysis^10^ and Fisher’s method^11^ are two standard and powerful approaches. For example, meta-analysis of summary statistics has been shown to be as powerful as mega-analysis of individual-level data, when there is no heterogeneity between the studies^12,13^. On the other hand, Fisher’s method is more robust to differential directions of effect by combining *p* values from different studies.

Recently, it has been shown that variant functional annotations can prioritize according to their biological relevance^14–18^. To overcome limitations such as incomparable metrics of measurement and differential ascertainment biases across different annotations, several authors have proposed methods to integrate multiple annotations into one single measure: a meta-score^19–23^. For instance, Kircher, M. et al.^22^ combined more than 60 genomic features into one combined annotation dependent depletion (CADD) meta-score to provide a measure of the relative deleteriousness for each variant, while Ionita-laza, I. et al.^23^ developed Eigen, a functional meta-score of similar nature using an unsupervised spectral approach.

Despite the popularity of using these meta-scores for genomic studies^24–26^, their potential for improving power of GWAS has not been well studied or understood. To integrate GWAS summary statistics with meta-scores, in addition to meta-analysis and Fisher’s method, we also consider the weighted *p* value approach^27^ and the stratified false discovery rate (sFDR) control method^28^, which extended the traditional FDR control methodology^29^. Both weighted *p* value and sFDR have been used to leverage linkage evidence^5,30^, gene-expression data^31,32^ and pleiotropy^33^ to increase power of GWAS. Here we use these data-integration methods to integrate CADD or Eigen functional meta-scores with GWAS summary statistics of 1132 phenotypes from the UK Biobank data^34^.

Integrating functional annotation scores with GWAS summary statistics has been previously studied. Recently, Kichaev, G. et al.^35^ proposed a modified weighted *p* value-based method called FINDOR to leverage polygenic functional enrichment to improve power of GWAS. To achieve this, FINDOR uses a stratified linkage disequilibrium (LD) score regression method^36^ to compute the expected $\chi ^2_1$ statistic for each GWAS SNP, by regressing the observed GWAS $\chi ^2_1$ statistics of the tagging SNPs against their 75 functional annotation scores^37^. FINDOR then stratifies the GWAS SNPs into 100 equally-sized bins based on their expected GWAS $\chi ^2_1$ values and applies bin-specific weights to the corresponding GWAS *p* values. An application of FINDOR by Kichaev, G. et al.^35^ to 27 traits, selected from the UK Biobank data^34^, showed that the method was able to improve power of GWAS by identifying additional associated variants. Based on FINDOR^35^, these 27 traits were constructed from “a set of 27 (roughly) independent and heritable traits, retaining only traits that exhibited a phenotypic correlation $r^2< 0.1$” and “to ensure adequate power to estimate functional enrichment, we also required that the traits have a heritability Z-score > 6 in the 145K dataset to be included in our analysis”.

To answer the question of whether prioritizing variants according to their biological function could improve power of GWAS, our study here is different from the FINDOR evaluation of Kichaev, G. et al.^35^ in several ways. First, unlike FINDOR, we use methods that prioritize GWAS findings based on external information alone to minimize concern of over-fitting. That is, the weighting factor and stratification are determined based on the annotations alone, independent of the observed GWAS summary statistics. Second, we utilize existing meta-scores that are already calibrated and easier to implement in practice, instead of using many individual functional scores. Third, we focus on evaluating methods’ robustness to the possibility of uninformative or even misleading functional annotations, because our understanding of the functionality of a genetic variant is incomplete and evolving. Finally, we comprehensively examine all 1132 UK Biobank traits for which the confidence for their SNP-heritability estimates were considered medium to high by Benjamin Neale’s lab from the Broad Institute (hereafter referred to as Nealelab; Web Resources). For each trait, we integrated GWAS summary statistics of close to 8 million common variants with their functional scores using three different data-integration methods: FINDOR with 75 individual functional scores, and weighted *p* value and stratified false discovery control methods with CADD (or Eigen) meta-scores.

In addition to the large-scale UK Biobank application, we also conducted a large-scale simulation study using different study designs, from leveraging the observed genomic data combined with simulated genetic data or vice versa to using only simulated data. We also considered different and complementary performance measures, from the traditional family-wise error rate (FWER) to false discovery rate (FDR), power, recall, precision, and relative efficiency. Finally, we sought to evaluate the functional annotation similarity between variants in linkage disequilibrium (LD), which has not been previously studied but an important consideration when integrating functional scores with GWAS.

## Results

### Method overview

Focusing on integrating functional scores with GWAS summary statistics to improve power of GWAS, we considered five data-integration methods, namely meta-analysis^10^, Fisher’s method^11^, weighted *p* value^27^, sFDR^28^, and FINDOR^35^. The meta-analysis and Fisher’s method were only included in some of the simulation studies to demonstrate that, although commonly used in many other scientific settings, they are not suitable for integrating GWAS with genomic functional scores.

Prior to the large-scale UK Biobank application using methods for which we understand their performance properties, we conducted a large-scale simulation study using three complementary study designs: (i) leveraging the observed functional annotations and integrating them with simulated GWAS, (ii) simulating functional annotations and combining them with observed GWAS, and (iii) using only simulated data. For an unbiased method evaluation, we also considered different performance measures, ranging from the traditional FWER to false discover control, and from the traditional power to recall, precision and relative efficiency.

**Figure 1:**
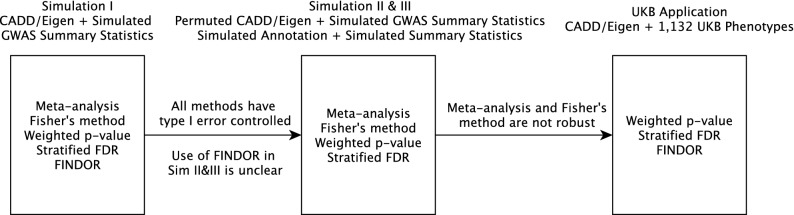
A visual summary of evaluating different methods across the simulation and application study settings.

In simulation study design I, we evaluated the type I error rate of all five methods. Overall, all methods showed reasonable type I error control in this setting. In simulation study designs II and III, we only evaluated four methods (meta-analysis, Fisher, weighted *p* value, and sFDR) because it was unclear if FINDOR remained valid when the LD structure between SNPs was not preserved. Based on the results from simulation study designs II and III, we found that meta-analysis and Fisher’s method had severe robustness issues to partially informative, uninformative or misleading additional information. Thus, we decided to exclude them from real data application. Finally, in the UK Biobank data application, we compared the recently proposed FINDOR method with weighted *p* value and sFDR, the two robust methods found through the earlier simulation studies. Figure 1 provides a visual summary of this process of evaluating different methods across the simulation and application study settings.

### Results of simulation design I, leveraging the observed genomic data

Here, simulated GWAS summary statistics, generated under the null of no association, were integrated with real functional annotations. The empirical FWER were estimated from 50,000 simulated replicates.

For the baseline analysis, using the null GWAS summary statistics alone, the empirical FWER is 0.0496 (Table S1). For the five different data-integration methods, the empirical rates are 0.0477, 0.0366, 0.0501, 0.0474, and 0.0537, respectively, for meta-analysis, Fisher’s method, weight *p* value, sFDR, and FINDOR, where FINDOR is the only method with slightly increased type I error rate. Although a method with an empirical FWER estimate outside [0.047, 0.053] can be considered inaccurate, overall all methods have reasonable type I error control in this setting.

Table S1 also provides a detailed account of the numbers of replicates, out of a total of 50,000 replicates, with at least one, two or three false findings for each of the methods; no method had more than three false findings per GWAS.

### Results of simulation design II, leveraging the observed genetic data

Here, real UK Biobank GWAS summary statistics of the 1132 traits were integrated with *permuted* CADD (or Eigen) meta-scores. We were unable to evaluate FINDOR in this setting, because FINDOR implements LD score regression (LDSC)^38^ and the validity of using LDSC for permuted annotation is unclear. The performance measures used here are $Recall_t={TP_{t}}/{m_{1,t}}$ and $Precision_t=1-FDR_t={TP_t}/{P_t}$, where $m_{1,t}$ is the number of genome-wide significant GWAS findings prior to data-integration for trait *t*, and $P_t$ and $TP_{t}$ are, respectively, the numbers of total positives and true positives after data-integration.

**Figure 2:**
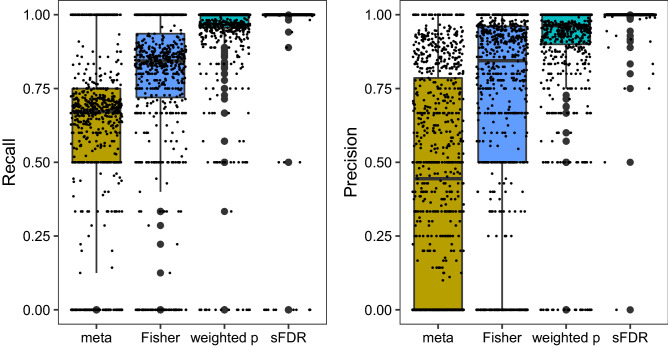
The *Recall* and *Precision* rates obtained from simulation study design II, integrating the 1132 UK Biobank GWAS summary statistics with *permuted* CADD functional meta-scores, using meta-analysis, Fisher’s method, the weighted *p* value approach, and the stratified FDR control. $Recall_t={TP_{t}}/{m_{1,t}}$ and $Precision_t=1-FDR_t={TP_t}/{P_t}$, where $m_{1,t}$ is the number of genome-wide significant independent loci prior to data-integration for trait *t*, and $P_t$ and $TP_{t}$ are the numbers of positives and true positives after data-integration; see Table S2 for additional results. Independent loci were defined using PLINK’s LDclumping algorithm with a 1 Mb window and an $r^2$ threshold of 0.1.

The *Recall* results shown in Fig. 2 confirm that meta-analysis and Fisher’s method are not suitable for integrating functional annotations with GWAS summary statistics. Across the 723 GWAS with at least one significant finding prior to data integration ($m_{1,t}>0$), the [Q1, median, Q3] *Recall* rates are [50%, 66.67%, 73.34%] for meta-analysis and [70%, 84.23%, 92.15%] for Fisher’s method after integrating *permuted* CADD scores. In contrast, these values are [95.87%, 100%, 100%] for the weight *p* value method and [100%, 100%, 100%] for the sFDR control. The *Precision* results in Fig. 2 corroborate the findings based on *Recall*.

The results here consistently show the sensitivity issue of meta-analysis and Fisher’s methods, and they confirm that sFDR is more robust than the weighted *p* value approach, which was demonstrated by Yoo, Y.J. et al.^5^ when integrating linkage results with GWAS. Results stratified by the four types of traits analyzed, nonsig, nominal, z4, and z7 (Figure S1), counting significant SNPs instead of loci (Figure S2), or using permuted Eigen scores (Figure S3) led to the same conclusion.

Permuting the meta-scores provides random functional annotations, independent of the GWAS summary statistics, for type I error evaluation. However, as noted earlier, it is of value to examine annotation similarities between SNPs in linkage disequilibrium. A similarity measure, $s^2_{i,j}$, was introduced to be compared with the LD measure, $r^2_{i,j}$. Results in Figure S4 show that there is no clear concordance between the two measures. A closer examination of $s^2_{i,j}$ and $r^2_{i,j}$ for two randomly selected regions is shown in Figure S5, and the contrast between variant-specific CADD meta-score and LD score across the genome is shown Figure S6. Both figures led to the same conclusion that functional scores of SNPs in strong LD are not necessarily similar.

### Results of simulation design III, varying the informativeness of genomic information

Here, both the GWAS summary statistics and the additional information available for data-integration were simulated, with varying degree of informativeness, including uninformative or possibly misleading annotation scores. The performance measures here are power and relative efficiency (*RE*), where *RE* was defined as one minus (the average ranks of the truly associated SNP after data-integration) divided by (their average baseline ranks using GWAS data alone).

**Figure 3:**
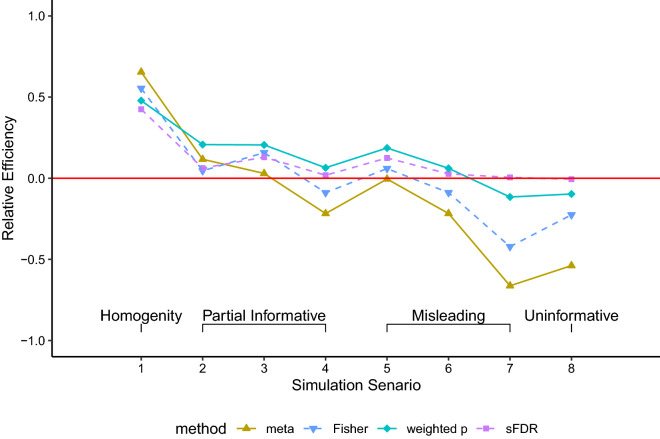
The relative efficiency (*RE*) obtained from simulation study design III, integrating simulated GWAS summary statistics with simulated additional information with varying degrees of informativeness, using meta-analysis, Fisher’s method, the weighted *p* value approach, and the stratified FDR control. There were 10,000 independent SNPs, among which 100 were truly associated whose summary statistics were drawn from *N*(3, 1); the rest from *N*(0, 1). For the additional information available for data integration, the details of the eight simulation scenarios are provided in the text and illustrated in Figure S7. *RE* is one minus (the average ranks of the truly associated SNP after data-integration) divided (by their average base-line ranks using GWAS data alone), averaged across 1000 simulation replicates.

The *RE* results in Fig. 3 are consistent with those from simulation study design II. While meta-analysis and Fisher’s method work well when the additional information is completely informative (i.e. the two data resources are homogeneous with each other), they are not suitable data-integration methods for this study setting.

The the *RE* results were consistent with the power results in Figure S8, across different rejection rules including controlling FWER at 5%, rejecting top 100 ranked SNPs, and controlling FDR at 5% or 20%. In addition, in Category II (partially informative), the *RE* of the different methods were compared as $\mu _{add}$ varied from 0.1 to 4 (Figure S9). As expected, all methods achieved higher *RE* as the informativeness of additional information (i.e. $\mu _{add}$) increased, with the weighted *p* value and sFDR methods being the most robust, when $\mu _{add}$ was relatively small. In Figure S10, we examined the impact of the number overlapping truly associated SNPs between the two sources under Category III (partial informative/misleading). We observed that both meta-analysis and Fisher’s method were unable to gain efficiency if the overlap is less than 80%. Consistent results were also observed when $\mu _1$ was varied from 0.1 to 4 to represent different power scenarios of a GWAS (Figures S11 to S18). Thus, meta-analysis and Fisher’s method were excluded from the application study.

### Results from integrating functional annotations to improve power of UK Biobank GWAS

The UK Biobank GWAS summary statistics from Nealelab (between each of the 7,895,174 common SNPs and each of the 1132 UK Biobank traits) were integrated with variant’s functional annotation scores using three data-integration methods, where FINDOR used 75 individual annotation scores, and weighted *p* value and sFDR used CADD (or Eigen) meta-scores as described earlier. The total number of independent, significant loci detected at the $5\times 10^{-8}$ level, as well as *Recall* and *New Discoveries* were calculated.

#### No striking improvements across the 1132 traits

**Figure 4:**
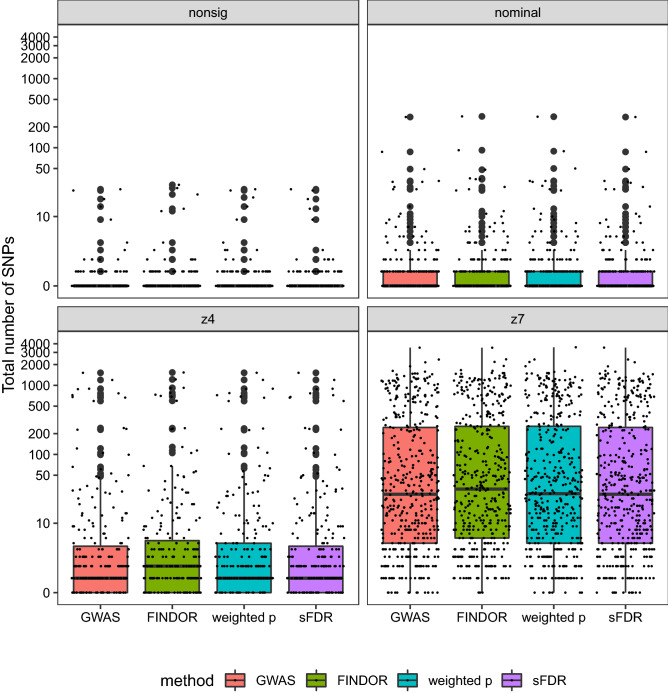
The total numbers of genome-wide significant independent loci of the UK Biobank GWAS application study, before and after data-integration with functional annotations, stratified by the four phenotype categories. In each figure, the total number of significant loci identified based on the UK Biobank GWAS data alone serves as a baseline. The GWAS baseline box-plot is followed by the box-plots for the total numbers of significant loci after integrating the UK Biobank GWAS summary statistics with functional annotations using FINDOR (using 75 individual annotation scores), and the weighted *p* value and stratified FDR control methods (each using the CADD meta-score), analyzing 7,895,174 variants for each of the 1132 UK Biobank traits. The 1132 traits were rated by Nealelab as having medium to high confidence for their heritability estimates, and they fall into four categories: nonsig (182 traits; heritability testing *p* value $p>0.05$), nominal (277 traits; $p < 0.05$), z4 (235 traits; $p < 3.17\times 10^{-5}$), and z7 (438 traits; $p < 1.28 \times 10^{-12}$). Independent loci were defined using PLINK’s LDclumping algorithm with a 1 Mb window and an $r^2$ threshold of 0.1.

Figure 4 shows the distributions of the total number of independent, significant loci identified by using GWAS alone (as a baseline, the first box-plot within each sub-figure) or after applying FINDOR, weight *p* value and sFDR data-integration methods, stratified by the four types of traits analyzed (nonsig, nominal, z4, and z7).

Overall, integrating the existing functional annotations with the UK Biobank GWAS association statistics did not lead to striking improvements, irrespective of the data-integration method. Results of using Eigen (Figure S19) or counting SNPs instead of independent loci (Figure S19) are characteristically similar.

The overall limited improvement is also evident from Table 1. For example, among the 1132 traits, prior to data-integration 772 have at least one genome-significant, independent loci. After data-integration these numbers are 738, 746 and 717 by, respectively, FINDOR, weighted *p* value and sFDR; the counts stratified by the four trait categories are also provided in Table 1. Similarly, 337 traits have more than ten significant loci prior to data-integration, and the numbers are 353, 346 and 337 post-data-integration by the three method.

**Table 1:** Results of the UK Biobank application study, before and after data-integration with functional annotations, stratified by the four phenotype categories.

	GWAS alone	After data-integration with functional annotation scores		
		75 individual scores	CADD meta-score	
		FINDOR	Weighted *p* value	Stratified FDR
**# of traits with > 0; 5; 10 significant loci**				
All traits	772; 402; 337	738; 420; 353	746; 408; 346	717; 403; 337
Nonsig	36; 5; 4	39; 5; 5	40; 5; 4	36; 5; 4
Nominal	110; 18,11	102; 21; 11	113; 21; 11	109; 18; 11
z4	160; 55; 42	169; 59; 45	172; 56; 43	157; 56; 42
z7	416; 324; 280	428; 335; 292	421; 326; 288	415; 324; 280
**[Q1, Median, Q3] of # of significant loci across traits**				
All traits	[0, 2, 18]	[0, 2, 21]	[0, 2, 20]	[0, 2, 18]
Nonsig	[0, 0, 0]	[0, 0, 0]	[0, 0, 0]	[0, 0, 0]
Nominal	[0, 0, 1]	[0, 0, 1]	[0, 0, 1]	[0, 0, 1]
z4	[0, 1, 5]	[0, 2, 6]	[0, 1, 5]	[0, 1, 5]
z7	[5, 27, 246]	[6, 32, 255]	[5, 27, 255]	[5, 27, 246]
**# of traits with > 0; 5; 10** ***New Discoveries***				
All traits	NA	553; 227; 165	472; 180; 139	89; 0; 0
Nonsig	NA	20; 0; 0	15; 0; 0	0; 0; 0
Nominal	NA	50; 3; 1	37; 1; 0	3; 0; 0
z4	NA	103; 22; 11	77; 16; 10	9; 0; 0
z7	NA	380; 202; 153	343; 163; 130	77; 0; 0
**[Q1, Median, Q3] of # of** ***New Discoveries*** **across traits**				
All traits	NA	[0, 0, 3]	[0, 0, 2]	[0, 0, 0]
Nonsig	NA	[0, 0, 0]	[0, 0, 0]	[0, 0, 0]
Nominal	NA	[0, 0, 0]	[0, 0, 0]	[0, 0, 0]
z4	NA	[0, 0, 1]	[0, 0, 1]	[0, 0, 0]
z7	NA	[1, 4, 19]	[1, 3, 14]	[0, 0, 0]

The three data-integration methods integrated the UK Biobank GWAS summary statistics with functional annotations using FINDOR (using 75 individual annotation scores), and the weighted *p* value andstratified FDR control methods (each using the Eigen meta-score), analyzing 7,895,174 variants for each of the 1132 UK Biobank traits. The 1132 traits were rated by Nealelab having medium to high confidence for their heritability estimates, and they fall into four categories: nonsig (182 traits; heritability testing $p>0.05$), nominal (277 traits; $p < 0.05$), z4 (235 traits; $p < 3.17\times 10^{-5}$), and z7 (438 traits; $p < 1.28 \times 10^{-12}$). See Table S1 for additional results.

Further, the intersection of significant loci between methods displayed in Figure S21 shows that out of a total of 59,764 significant loci identified in the z7 category, 46,631 (78%) were common across the three data-integration methods *and* the baseline GWAS alone. Additionally, Figure S22 shows that the total numbers of significant loci after data-integration are similar to those based on GWAS alone, for all three data-integration methods and across all 1132 traits.

#### *New Discoveries* for the 182 traits in the nonsig category

Although the ground-truth is unknown in application studies, the *New Discoveries* for traits in the nonsig category may be considered as false positives, as their SNP-heritablity testing *p* values were $>0.05$ and the inferences were given “medium” or “high” confidence by Nealelab; traits in this category include, for example, “Fizzy drink intake”, “Apple intake”, “Time spent doing moderate physical activity”, and “Work hours”.

**Figure 5:**
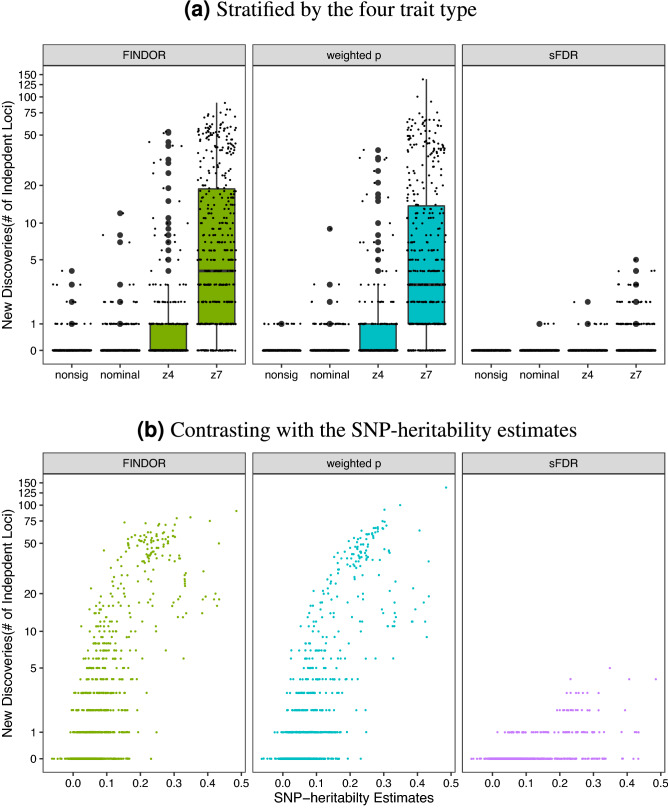
The total numbers of genome-wide significant independent loci of the UK Biobank GWAS application study, before and after data-integration with functional annotations, stratified by the four phenotype categories. In each figure, the total number of significant loci identified based on the UK Biobank GWAS data alone serves as a baseline. The GWAS baseline box-plot is followed by the box-plots for the total numbers of significant loci after integrating the UK Biobank GWAS summary statistics with functional annotations using FINDOR (using 75 individual annotation scores), and the weighted *p* value and stratified FDR control methods (each using the CADD meta-score), analyzing 7,895,174 variants for each of the 1132 UK Biobank traits. The 1132 traits were rated by Nealelab as having medium to high confidence for their heritability estimates, and they fall into four categories: nonsig (182 traits; heritability testing *p* value $p>0.05$), nominal (277 traits; $p < 0.05$), z4 (235 traits; $p < 3.17\times 10^{-5}$), and z7 (438 traits; $p < 1.28 \times 10^{-12}$). Independent loci were defined using PLINK’s LDclumping algorithm with a 1 Mb window and an $r^2$ threshold of 0.1.

Among the 182 traits in the nonsig category, 20, 15 and 0 traits had at least one *New Discoveries* after data-integration using, respectively, FINDOR, and weighted *p* value and sFDR when using CADD (Table 1 and Fig. 5). Weighted *p* value and sFDR using Eigen led to 9 and 1 traits with at least one *New Discoveries* (Table S2). Reassuringly, no data-integration methods led to more than five *New Discoveries* for any of the 182 traits in the nonsig category.

#### *New Discoveries* for the 438 traits in the z7 category

The number of *New Discoveries* increases as a trait’s SNP-heritability estimate increases (Fig. 5b), and there are increased numbers of *New Discoveries* for the 438 traits in the z7 category (Table 1). This is consistent with the results by Kiachaev et al.^35^ who studied 27 highly heritable traits in the UK Biobank, which we replicated here. However, among the 27 supposedly uncorrelated traits studied by Kiachaev et al.^35^, we note that traits with data field 30050 (mean corpuscular hemoglobin) and 30010 (red blood cell (RBC) count) have phenotypic correlation of − 0.51 and genetic correlation of − 0.66, using Nealab co-heritability browser (see Web Resources).

Among the 438 traits in the z7 category, 380, 343 and 77 traits had at least one *New Discoveries* after data-integration using, respectively, FINDOR, and weighted *p* value and sFDR when using CADD (Table 1 and Fig. 5). Weighted *p* value and sFDR using Eigen led to characteristically similar results (Table S2 and Figure S23).

Additionally, FINDOR and weighted *p* value led to more than ten *New Discoveries* for, respectively, 153 and 130 traits in the z7 category. However, the two methods also led to loss of significant loci that were present in the baseline GWAS, resulting in similar total numbers of significant loci before and after data-integration (Figure S24).In general, FINDOR and weighted *p* value methods yielded similar performance, which is somewhat expected as FINDOR applies the weighted *p* value principle. Both methods have noticeably more findings than sFDR. This is also expected given the trade-off between power and robustness, which we explore further.

#### Trade-off between *New Discoveries* and *Recall*

**Figure 6:**
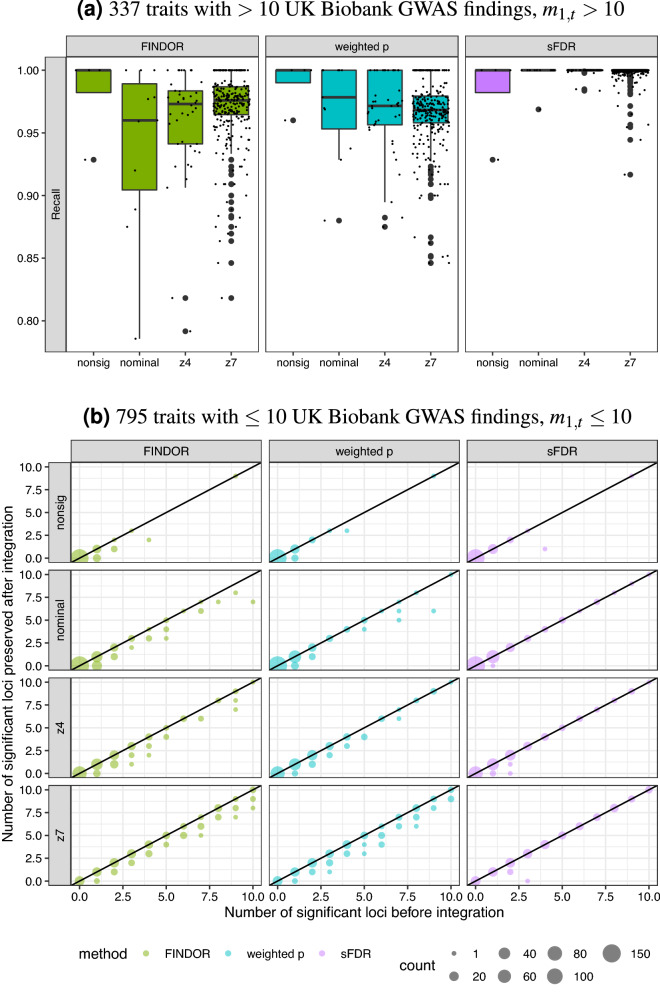
Results of the UK Biobank GWAS application study, before and after data-integration with functional annotations, stratified by the four phenotype categories. (**a**) $Recall_t={TP_{t}}/{m_{1,t}}$, where $m_{1,t}$ is the number of genome-wide significant independent loci prior to data-integration for trait *t*, and $TP_{t}$ is the number of true positives after data-integration. *Recall* estimation is not stable when $m_{1,t}$ is small so for $m_{1,t}\le 10$, (**b**) contrasts the number of significant loci preserved after data-integration with $m_{1,t}$. The three data-integration methods integrated the UK Biobank GWAS summary statistics with functional annotations using FINDOR (using 75 individual annotation scores), and the weighted *p* value andstratified FDR control methods (each using the CADD meta-score), analyzing 7,895,174 variants for each of the 1132 UK Biobank traits. The 1132 traits were rated by Nealelab having medium to high confidence for their heritability estimates, and they fall into four categories: nonsig (182 traits; heritability testing $p>0.05$), nominal (277 traits; $p < 0.05$), z4 (235 traits; $p < 3.17\times 10^{-5}$), and z7 (438 traits; $p < 1.28 \times 10^{-12}$). Independent loci were defined using PLINK’s LDclumping algorithm with a 1 Mb window and an $r^2$ threshold of 0.1.

Figure 6a shows the *Recall* for the 337 traits with more than ten GWAS signals prior to data-integration ($m_{1t}>10$), as the stability of a *Recall* estimate depends on $m_{1t}$. For the 795 traits with $m_{1t}\le 10$, instead of showing $P_{t}/m_{1t}$, Fig. 6b contrasts $P_{t}$ with $m_{1t}$; see Figure S25(A) for *Recall* of all traits and Figure S25(B) for *Recall* versus SNP-heritablity estimates.

It is clear that sFDR has better *Recall* than either FINDOR or weighted *p* value for the traits in the nominal, z4 and z7 categories. For example, for the 280 traits in the z7 category with at least one significant finding prior to data-integration, the median *Recall* is 97.6%, 96.8% and 100%, respectively, for the FINDOR, weighted *p* value and sFDR methods (Table S2).

The trade-off between power and robustness is also supported by results in Figure S26 (*Recall* of the 402 traits with $m_{1,t}>5$), and other supplementary figures including Figures S27 ($P_{t}$ versus $m_{1t}$ for traits with $m_{1,t}\le 50$), Figure S28 (traits with $m_{1,t}\le 10$) and Figure S22 (for all traits), as well as Figure S29 (*Recall* of weighted *p* value and sFDR using Eigen, instead of CADD). It is also clear that, *Recall* increases as a trait SNP-heritability estimate increases (Figure S25(B)), which was also observed for *New Discoveries* (Fig. 5b).

## Discussion

There has been much discussion about the value of integrating functional annotations into genetic association studies^39–41^, but theoretical evaluation and large-scale application to test this hypothesis has been limited^35^. In addition to conducting comprehensive simulation studies, we performed a large-scale application study of all 1132 traits in the UK Biobank, for which the SNP-heritability estimates were given “medium” or “high” labels by Nealelab. For each trait, we integrated GWAS summary statistics of close to 8 million common variants with their functional scores using three different data-integration methods: FINDOR with 75 individual functional scores, and weighted *p* value and stratified false discovery control methods with CADD (or Eigen) meta-scores.

We observed that, although the numbers of new genome-wide significant findings after data-integration increase as trait SNP- heritability estimates increase, there is a trade-off between new findings and loss of the original GWAS findings. This resulted in similar total numbers of significant findings between using GWAS alone and integrating GWAS with functional scores, across all 1132 traits analyzed and all three data-integration methods considered. A closer examination of method performance and trait heritability revealed that all methods performed better (more *New Discoveries* and higher *Recall*) for traits with higher estimates of SNP-heritability (Figs. 5B and S25(B)).

Our study used CADD and Eigen as the functional meta-score available for data-integration using weighted *p* value and sFDR. To the best our knowledge, CADD was the first meta-score in the literature and Eigen was the first to use unsupervised learning approach, and both meta-scores have been shown to be superior to other scores in some genomic studies^22,23^. However, the recent work by Li, X. et al.^24^ has proposed annotation-PCs, an alternative that warrants further investigation.

In one of our simulation studies, we permuted CADD (and Eigen) to provide a set of meta-scores that are independent of the GWAS summary statistics of the UK Biobank data. Although this approach is valid for examining type I error control, we were intrigued by the question of whether meta-scores of SNPs in strong LD are similar. To answer this question, we defined $s^2_{i,j}=1-{|CADD_i-CADD_j|}/{(CADD_i+CADD_j)}$ as the functional similarity measure between SNPs *i* and *j*. Interestingly, there was no clear concordance between $s^2_{i,j}$ and $r^2$, the LD measure for genotype similarity (Figures S4 and S5). Additionally, there was no relationship between CADD and LD score (Figure S6).

Throughout this paper, we have used the default tuning parameter values, $\beta =2$ for the weighted *p* value approach and $k=2$ for the sFDR method. We did not tune the parameters to select values that lead to the ‘best’ results, for which valid result interpretation requires adjustment for the inherent data-dredging or selective inference. The choice of different $\beta$ and *k* values, however, has an effect on method performance. Figure S30 shows the results of the full analysis of the UK Biobank application study. For the weighted *p* value approach, the default $\beta =2$ led to the highest number of $New \, Discoveries$, but at the same time it resulted in the lowest *Recall* rate. For the sFDR method, $k=10$ or 20 lead to an increased number of $New \, Discoveries$ as compared with the default $k=2$, at the cost of slightly reduced *Recall* rates. Thus, unlike the previous linkage and GWAS integration setting, the default value of $k=2$ for sFDR appears to be sub-optimal for integrating functional meta-score with GWAS.

Among the five data-integration methods, despite meta-analysis and Fisher’s method being applicable in many scientific settings, their applications to genetic association studies are typically restricted to combining evidence from multiple GWAS of the same phenotype and from the same population. This is because the statistical power of meta-analysis (and Fisher’s method) relies on the assumption of homogeneity beyond direction of effect^42^. In practice, given two families of multiple tests, the underlying compositions of the null and alternative hypotheses may differ, unless the two studies used the same study design including phenotype definition, genotyping platform, environmental exposure, and study population^43^. When the truly associated SNPs do not completely overlap between the different studies, using random-effect (instead of fixed-effect meta-analysis) does not guarantee improved power, because it violates the assumption that the effect sizes come from the *same* distribution.

The use of meta-analysis and Fisher’s method is also questionable when $z_i$ and $z_{i, add}$ from the two studies offer different types of information. In essence, the use of weights $\sqrt{1/v_i}$ and $\sqrt{1/v_{i,add}}$ notwithstanding, meta-analysis and Fisher’s method implicitly assume $z_{i}$ and $z_{i, add}$ carry ‘exchangeable’ information. For our study, however, $z_i$ is the genetic association summary statistic, while $z_{i,add}$ is the genomic annotation meta-score. Thus, meta-analysis and Fisher’s method are likely to be sub-optimal for the purpose of this study. However, for completeness we include the two classical data-integration methods in our initial method evaluation.

Compared to meta-analysis and Fisher’s method, weighted *p* value and sFDR are more natural choices when only integrating one piece of additional information (e.g. functional annotation meta scores), while FINDOR is more suitable when integrating a set of functional annotations. All of these integration methods only require summary statistics, and once functional annotations are prepared in appropriate format, all methods can compute millions of SNPs within a few minutes. Possible limitations of weighted *p* value include the difficulty of handling categorical additional information. This is also true for FINDOR, but can be easily handled using sFDR. The performances of all methods, however, are likely to be affected by different, subjective choices of groups and weighting schemes, and gold standard stratification and weight do not yet exist in this setting. Overall, FINDOR and weighted *p* value were similar to each other, and they led to more new discoveries for traits considered heritable as compared with sFDR (the traits in the nominal, z4 and z7 categories; Fig. 5), but at the cost of lower Recall rates (Fig. 6).

## Conclusions

The classical meta-analysis and Fisher’s method are not suitable for integrating functional annotations with GWAS summary statistics, as calibrating evidence between the two data sources is difficult. When the functional annotations are truly informative, FINDOR and weighted *p* value methods are more powerful than sFDR, but sFDR is more robust to uninformative or even misleading added information. In the application to the UK Biobank data, none of the methods led to striking improvements. This suggests the need for more informative functional scores and/or new data integration methods to further improve the power of GWAS through leveraging variant functional annotations. It is important to note that this conclusion applies to bi-allelic common autosomal variants with MAF greater than 1%, which may not be generalizable to rare variants.

Potential future work include (1) leveraging cell-type-specific annotations as complex traits often exhibit cell-type-specific functional enrichments^44^; (2) obtaining GWAS summary statistics for previously understudied variants e.g. in coding sequence, which tend to have higher functional effects than the variants currently studied as they came from the exome sequencing data of the UK Biobank^45^.

## Methods

### The integration methods: meta-analysis, Fisher’s method, weighted *p* value, and stratified false discovery rate (sFDR) control

#### Notation and set-up

Let $z_i$ and $p_i$ be the association test statistic and its corresponding *p* value for SNP *i*, $i=1,...m$, from a genome-wide association study, the primary data of interest. Without loss of generality, we assume $z_i$ follows *N*(0, 1), the standard normal distribution, under the null hypothesis of no association between the SNP and the GWAS trait under the study.

Let $z_{i,add}$ and $p_{i,add}$ be additional information available for the SNP, based on data *independent* of $z_i$ and $p_i$ from the GWAS. Note that $z_{i,add}$ may or may not be normally distributed depending on the application setting, e.g. $z_{i,add}$ can be the CADD^22^ or Eigen^23^ functional meta-score available for SNP *i*, which will be the focus of this study.

#### Meta-analysis and Fisher’s method

For the meta-analysis approach, we first assume the best-case scenario where $z_{i,add}$ is normally distributed. We then use the inverse variance approach^46^ to integrate $z_i$ and $z_{i,add}$, $Z^{meta}_{i}={(\sqrt{1/v_{i}}\, z_i+\sqrt{1/v_{i,add}}\, z_{i,add})}/{\sqrt{1/v_{i}+1/v_{i,add}}}\,$, where the weights depend on $v_{i}$ and $v_{i,add}$, the variance estimates associated with, respectively, the GWAS and the additional study available for data integration. Under the null hypothesis of no association *and* assuming the functional meta-score is uninformative, $Z^{meta}_{i}$ is *N*(0, 1) distributed.

Fisher’s method combines *p* values instead of the test statistics, $Z_{i}^{Fisher}=-2(\log (p_i)+\log (p_{i,add}))$. Fisher’s method is omnibus to directions of effect, and as a result it can be more powerful than meta-analysis when signs of $z_i$ and $z_{i,add}$ differ. Under the null that both $p_i$ and $p_{i,add}$ are independently $\text {Unif}(0,1)$ distributed, $Z_{i}^{Fisher}$ is $\chi ^2_{df=4}$ distributed.

Although meta-analysis and Fisher’s method are applicable in many scientific settings, their applications to genetic association studies are typically restricted to combining evidence from multiple GWAS of the same phenotype and from the same population. This is because the statistical power of meta-analysis (and Fisher’s method) relies on the assumption of homogeneity beyond direction of effect^42^. In practice, given two families of multiple tests, the underlying compositions of the null and alternative hypotheses may differ, unless the two studies used the same study design including phenotype definition, genotyping platform, environmental exposure, and study population^43^. When the truly associated SNPs do not completely overlap between the different studies, using random-effect (instead of fixed-effect meta-analysis) does not guarantee improved power, because it violates the assumption that the effect sizes come from the *same* distribution. However, for completeness we include the two classical data-integration methods in our initial method evaluation.

For a practical implementation of meta-analysis when $z_{i,add}$ is the CADD or Eigen meta-score, we used equal weights as the sample size of a functional study is not suitable. Further, we used the inverse normal transformation to re-scale $z_{i,add}$ while keeping the sign of the re-scaled $z_{i,add}$ to be the same as $z_{i}$, creating the best-case scenario for the meta-analysis. Similarly, for a practical implementation of Fisher’s method, we use a rank-based transformation and let $p_{i,add}=(\text {rank of } z_{i,add}/m$), which is also related the phred-scaled CADD and Eigen scores which we discuss later.

#### The weighted *p* value approach

Unlike meta-analysis and Fisher’s method, which assume $z_{i}$ and $z_{i, add}$ carry similar information, the weighted *p* value approach^27^ treats $z_{i}$ and $z_{i, add}$ differently. That is, the method considers $z_{i}$ and $p_i$ as the primary data of interest, and it transforms $z_{i, add}$ to $w_i$, a weight to be applied to $p_i$. Thus, the weighted *p* value approach is an attractive method for this study setting, where the primary data are GWAS summary statistics, and the additional information available are genomic functional scores derived *independently* from the GWAS of interest.

For a valid weighted *p* value implementation, the $w_i$’s must satisfy two conditions: $w_i \ge 0$ and ${\bar{w}}=\sum w_i/m = 1$^27^. To transform $z_{i,add}$ to $w_i$^30^, studied two possible weighting schemes: exponential, $w_i = m(\exp (\beta \times z_{i,add})/\sum _{i}\exp (\beta \times z_{i,add}))$, and cumulative,

1 $$\begin{aligned} w_i = m\frac{\Phi (z_{i,add}-\beta )}{\sum _{i}\Phi (z_{i,add}-\beta )}, \end{aligned}$$

where $\Phi$ is the cumulative distribution function of the standard normal. In either case,

2 $$\begin{aligned} p_{i,weighted} = \min \left\{ \frac{p_i}{w_i},1\right\} . \end{aligned}$$

Here we choose the cumulative weighting scheme, with the recommended default value of $\beta =2$^30^. This is because the exponential weighting scheme is highly sensitive to large values of $z_{i,add}$, which is the case here; functional meta-scores can be as large as 80^22^.

#### Stratified false discovery rate (sFDR) control

Unlike the weighted *p* value approach that up- or down-weights each SNP according to its external information $z_{i,add}$, the sFDR method separates the GWAS SNPs into different groups based on $z_{i,add}$, which can be categorical or continuous^28^. When $z_{i,add}$ is continuous, it has been shown that categorizing $z_{i,add}$ does not necessarily result in loss of power, as the additional information available are unlikely to be precisely informative^5^. In addition, sFDR is robust to the situation when $z_{i,add}$ is uninformative (i.e. random) or possibly misleading.

To implement sFDR in our setting where $z_{i,add}$ is the continuous functional meta-score, without loss of generality, we first stratify GWAS SNPs into two groups based on whether their meta-scores are among the top five percent or not, *irrespective* of $z_{i}$ and $p_{i}$, the GWAS summary statistics. (The choice of the number of groups and thresholds, however, is subjective, similar to choosing the weighting scheme and $\beta$ value for the weighted *p* value approach above.) As a result, there are two groups of GWAS SNPs, where group 1 contains 5% of the GWAS SNPs with the highest functional meta-scores and group 2 contains the remaining SNPs. It is worth emphasizing that group 1 is only presumed to be the high-priority group, as the stratification is based on genomic $z_{i,add}$ alone, independent of the GWAS $z_i$ or $p_i$.

We then apply FDR control, separately, to the two groups of GWAS *p* values, but using the same pre-specified FDR $\gamma$% level. Following the sFDR method of Sun, L. et al.^28^, for each group of SNPs we first transform their GWAS *p* values, $p_i$’s, to q-values, $q_i$’s^47^, and we then reject the SNPs with $q_i<\gamma$%; this sFDR procedure controls the overall FDR at the $\gamma$% level. Although sFDR does not explicitly use weights, group-specific weights can be derived^5^.

Let $m^k$ be the number of SNPs in group *k*, and let $\pi _0^{(k)}$ be the proportion of null SNPs in the group. Within each group, we obtain q-values recursively^47^, $q_i = \min \{{\hat{\pi }_0^{(k)}m^{(k)}p_{(i)}}/{i},q_{i+1}\},$ where $p_{(1)}\le \cdots \le p_{(i)}\le \cdots \le p_{(m)}$ are the ordered GWAS *p* values, and the procedure starts from $q_{(m)}=\widehat{\pi_0}p_{(m)}$. To obtain $\widehat{\pi_0}$, we choose the commonly used conservative estimate^48^, $\widehat{\pi_0}=\{\text {the number of SNPs with } p_i >0.5\}/\{0.5 m^{(k)}\}$.

After rejecting SNPs with $q_i < \gamma$% separately for each group of SNPs, let $\alpha ^{(k)}$ be the maximum GWAS *p* values among the rejected SNPs for group *k*, the group-specific weight is,

3 $$\begin{aligned} w^{(k)} = m\frac{\alpha ^{(k)}}{\sum _k \alpha ^{(k)} m^{(k)}}. \end{aligned}$$

We can then obtain sFDR weighted *p* values,

4 $$\begin{aligned} p_{i,sFDR} =\min \{\frac{p_i}{w^{(k)}},1\}. \end{aligned}$$

If group 1 has no rejections at the pre-specified FDR $\gamma$% level, we set $w^{(1)}=0$ and $w^{(2)}={m}/{m^{(2)}}$. Similarly, if group 2 has no rejections, $w^{(1)}={m}/{m^{(1)}}$ and $w^{(2)}=0$. If both groups have no rejections at the $\gamma$% level, then $w^{(1)}=w^{(2)}=1$. That is, the study is reduced to the unweighted case.

The sFDR group-specific weights, $w^{(k)}$’s, satisfy the constraints imposed by the weighted *p* value approach^27^, and they have been shown to be a robust version of the SNP-specific $w_i$’s^5^. If the additional information is truly informative, $w^{(1)}>1$ while $w^{(2)}<1$, and they can be considered as dichotomized $w_{i}$’s of the weighted *p* value approach. In that case, the weighted *p* value approach is more powerful than sFDR. On the other hand, if the information is just random noise, $w^{(1)}\approx w^{(2)} \approx 1$ for sFDR, while the weighted *p* value method still up- or down-weights the GWAS *p* values according to the SNP-specific $w_i$’s, which are proportional to the observed $z_{i,add}$’s. In the event of misleading information, $w^{(1)}<1$ while $w^{(2)}>1$ even though group 1 was presumed to be the high-priority group. Thus, sFDR is robust to uninformative or even misleading added information.

### The UK Biobank GWAS summary statistics for 1132 complex traits

We obtained the UK Biobank GWAS round 2 summary statistics from Nealelab (Web Resources). Nealelab performed association studies for 4236 complex traits using regression model with additively coded genotype, as well as age, sex and the first 20 principal components as covariates. For each of these traits, Nealelab also applied the LD-score regression method^38^ to estimate the SNP-heritability, which ranges from 0 to 48%.

In addition to testing if the SNP-heritability is 0%, Nealelab also provided a confidence level (“low”, “medium” or “high”) to the heritability inference for each trait. Thus, we restricted our analysis to the 1132 traits denoted with “medium” or “high” confidence labels, which were primarily based on the effective sample sizes $>20{,}000$. Of the 1132 traits analyzed, 531 are continuous and 601 are binary traits. For a binary trait, the effective sample size depends on the number of cases or controls; see Figure S31 for a histogram of the case rates for the 601 binary traits. Nealelab then classified these 1132 traits into four categories: nonsig (182 traits with SNP-heritability testing *p* value $p>0.05$), nominal (277 traits; $p<0.05$), z4 (235 traits; $p<3.17\times 10^{-5}$), and z7 (438 traits; $p<1.28\times 10^{-12}$), where the nonsig category can serve a negative control for the purpose of this study. Figure S32 contrasts the heritability $h_{g}^2$ estimates of the 1132 traits with their SNP-heritability testing z-values.

The UK Biobank GWAS results of Nealelab were derived from $n=361{,}194$ individuals of white-British ancestry and 10.9 million variants that passed a set of quality control (QC) steps; see Web Resources for the detailed QC steps performed by Nealelab. Our data-integration analysis focused on $m=7{,}895{,}174$ common bi-allelic autosomal SNPs. We excluded indel variants because their functional meta-scores are unavailable. We additionally excluded X-chromosomal variants because their functional annotations are not always available and the association testing may not be optimal^49^. Lastly, we excluded SNPs with minor allele frequency (MAF) less than 1%, as joint analysis of multiple rare variants simultaneously^50^ is beyond the scope of our study.

### CADD and Eigen functional meta-scores

We obtained the CADD meta-scores (v1.6), using the CADD tool^51^, and the Eigen meta-scores (v1.0), using the ANNOVAR tool^52^, for all the 7,895,174 common, bi-allelic autosomal SNPs.

In addition to the raw CADD meta-scores, the CADD tool also made available rank-based scores called phred scores,

$$\begin{aligned} -10\log _{10}(\text {ranks of the raw scores/the total number SNPs}); \end{aligned}$$

the phred-scaled scores are positive and have better interpretation compared to the raw scores. For example, a phred score of 10 or greater indicates that the SNP is predicted to be among the top 10% most deleterious variants of the human genome, while a phred score 20 or greater implies top 1% most deleterious.

For consistency between CADD and Eigen, we similarly obtained phred-scaled Eigen scores. Figure S33 in Supplementary Information shows the histograms of CADD and Eigen phred-scaled scores; each is expected to be $2.17 \chi ^2_2$ distributed, because the ranks of the raw scores/the total number SNPs are Unif(0,1) distributed, and $-2\log (\text {Unif(0,1)})$ is $\chi ^2_2$ distributed; hereafter scores mean phred-scaled scores unless specified otherwise.

Because Eigen scores were calculated using an unsupervised learning approach, in contrast to CADD scores inferred using labeled data, we also compared these two scores genome-wide (Figure S34) and across four different consequence categories (Figure S35): missense, non-coding, synonymous, and protein truncating variants (PTV). Variants in in the missense and PTV categories tend to have higher CADD than Eigen scores, while variants in the non-coding and synonymous categories tned to have higher Eigen than CADD scores. However, overall the two meta-scores are consistent and led to qualitatively comparable data-integration results, which we discuss next.

### Simulation study design I, leveraging the observed genomic data

Here we used the real CADD and Eigen functional meta-scores, combined with simulated GWAS summary statistics, to evaluate type I error control of the data-integration methods examined.

#### Simulated GWAS summary statistics under the null of no association combined with real functional annotation scores

To simulate GWAS summary statistics that contain realistic LD patterns, we utilized the publicly available genotype data of the 1000 Genomes Project^53^. Independent of the observed genotype data, we simulated trait values, from *N*(0, 1), for 1756 individuals from the 1000 Genomes Project who are unrelated to each other^54^. We examined 422,923 autosomal, bi-allelic and common ($\hbox {MAF} >5\%$) SNPs that (a) passed the quality control conducted by Rosilin, N.M. et al.^54^, (b) have CADD and Eigen meta-scores available, and (c) have the 75 annotations used by FINDOR.

We then obtained GWAS summary statistics for the 422,923 SNPs by regressing the trait values of the 1,756 individuals on their additively coded genotypes. Because the trait values were randomly generated, independent of the genotypes and populations, the resulting GWAS $z_i$’s are *N*(0, 1) distributed and $p_i$’s Unif(0,1) distributed, as expected under the null of no association; the histograms of $z_i$’s and $p_i$’s from one randomly selected simulation run are shown in Figure S36.

Finally, we integrated the GWAS summary statistics with their corresponding CADD (or Eigen) meta-scores using the four methods, meta-analysis, Fisher’s method, weighted *p* value, and sFDR control as described above. Although Kichaev, G. et al.^35^ showed that FINDOR calibrates well when a GWAS consists of a mixture of null and associated SNPs, we also examined the performance of FINDOR in this setting when all GWAS SNPs are under the null hypothesis of no association. We applied the FINDOR tool using the same set of LDscores and the 75 annotations^37^ that were used by Kichaev, G et al.^35^ for their study; see Web Resources.

#### Method evaluation: family-wise error rate (FWER)

For each simulation replicate (i.e. a GWAS simulated under the null of no association combined with real functional scores), we obtained the number of false positives using the conservative Bonferroni corrected significance level, $\alpha =0.05/422923=1.2\times 10^{-7}$. We repeated the simulation, independently, 50,000 times, and calculated the FWER as the proportion of the number of replicates with at least one significant finding. Assuming the true FWER is 0.05, we expect the FWER estimate obtained from the 50,000 independent simulation replicates to have a standard error of $\sqrt{0.05\times 0.95/50000}\approx 0.001$. Thus, a method with an empirical FWER outside [0.047, 0.053] can be considered inaccurate.

### Simulation study design II, leveraging the observed genetic data

Here we combined the observed UK Biobank GWAS summary statistics with *permuted* CADD (or Eigen) scores to evaluate robustness of a method to random annotation scores. Prior to the permutation, we examined the similarity of functional annotations between SNPs in linkage disequilibrium.

#### Permuted functional annotation scores combined with real GWAS summary statistics

Permutation does not preserve the potential correlation between functional scores of nearby SNPs. However, for the purpose of evaluating type I error control, it provides a valid set of annotation scores that are independent of the GWAS summary statistics. Nevertheless, we examined if SNPs in strong LD have similar annotation scores, as this has not been previously studied.

Using CADD as an example, let $CADD_i$ and $CADD_j$ be the annotation scores of SNPs *i* and *j*, respectively. We first defined a pair-wise similarity measure as $s^2_{i,j}=1-{|CADD_i-CADD_j|}/{(CADD_i+CADD_j)}$. The measure $s^2_{i,j}$ is bounded between 0 and 1, where 1 means two scores are identical whereas a value close to 0 suggests a lack of similarity. We then contrasted $s^2_{i,j}$ with $r^2_{i,j}$, the traditional LD measure of genotype similarity between two SNPs.

After we permuted the functional scores of the 7,895,174 common, bi-allelic autosomal SNPs, for each of the 1132 traits of the UK Biobank data, we integrated the GWAS summary statistics with the permuted annotation scores, using meta-analysis, Fisher’s method, weighted *p* value, and sFDR control. We were not able to evaluate FINDOR here, because FINDOR implements the LD SCoring (LDSC) tool^38^ and the validity of using LDSC for permuted annotation is not clear.

#### Method evaluation: *Recall*, *Precision* and *FDR*

Before data integration, we first used $\alpha =5\times 10^{-8}$^55^ to identify genome-wide significance findings, $m_{1,t}$, for each trait *t*, $t=1,\ldots , 1132$, based on the UK Biobank summary statistics alone. For the purpose of this simulation study, we treated $m_{1,t}$ as the total number of truly associated SNPs to be discovered after data-integration for trait *t*. In addition to counting the number of significant SNPs per GWAS, we also counted the number of independent, significant loci. We first defined independent loci using the LDclumping algorithm of PLINK (v1.07)^56^, with a sliding window of 1 Mb and a LD $r^2$ threshold of 0.1 as per standard practice. We then considered a locus significant if it contained at least one genome-wide significant SNP.

After integrating the UK Biobank GWAS summary statistics with *permuted* functional scores for each trait *t*, we used the same $\alpha =5\times 10^{-8}$ to identify genome-wide significance findings (SNPs or loci as defined above), denoted as $P_t$. Among the $P_t$ positives, we defined false positives, $FP_t$, as the new findings that were not part of $m_{1,t}$, because the information used here for data integration were random noise. Similarly, we defined $TP_t=P_t-FP_t$ as the number of true positives for trait *t*.

Finally, we defined and calculated recall, precision and false discovery rate by

$$\begin{aligned} Recall_t=\frac{TP_{t}}{m_{1,t}},\,\, Precision_t=\frac{TP_t}{P_t} \,\, \text { and } \,\, FDR_t=1-Precision_t=\frac{FP_t}{P_t}. \end{aligned}$$

*Recall* is conceptually the same as *Power*, defined later for our simulation studies where the ground truth is known and $m_{1,t}$ SNPs were simulated as truly associated SNPs. We calculated $Recall_t$ only when $m_{1,t}>0$. That is, for the 409 out 1132 traits with no GWAS significant findings *before* data-integration (i.e. $m_{1,t}=0$) we did not calculate $Recall_t$. Regardless of whether $m_{1,t}=0$ or not, for traits with no significant findings *after* data-integration (i.e. $P_t=0$) we conservatively defined $Precision_t=1$ and $FDR_t=0$.

### Simulation study design III, varying the informativeness of genomic information

To further investigate method performance in the presence of completely informative, partially informative, uninformative, or even misleading added information, we performed an additional set of simulation studies. Although LD is an important aspect of GWAS, given the simulation study designs I and II and our findings in the results section, the simulation studies here focused on independent SNPs to delineate other potentially influencing factors.

Without loss of generality, we assumed the total number of SNPs $m=10{,}000$, among which the first $m_1=100$ SNPs are truly associated. The corresponding summary statistics $z_i$’s were drawn, independently, from $N(\mu _1,1)$ for the $m_1$ associated SNPs, and from *N*(0, 1) for the remaining null SNPs. The top left plot in Figure S7 shows the Manhattan plot for one simulated GWAS replicate with $\mu _1=3$; we also varied $\mu _1$ from 0.1 to 4 to represent different power scenarios of a GWAS.

We then assumed $z_{i,add}$’s as the additional information available, which were drawn, independently, from $N(\mu _{add},1)$ for the $m_{add}$ SNPs and from *N*(0, 1) for the remaining SNPs. Importantly, the locations of the $m_{add}$ SNPs may differ from those of the $m_1$ associated SNPs. That is, the additional information available for a truly associated SNP may be random noise. On the other hand, for a null SNP with no association (i.e. $z_i$ drawn from $N(\mu _1,1)$), its $z_{i,add}$ could be drawn from $N(\mu _{add},1)$, representing misleading information. We also varied $\mu _{add}$, which may or may not be the same as $\mu _1$.

Using $m_1=100$ and $\mu _1=3$ as an example for the GWAS component, we considered the following eight scenarios for the additional information available for data integration (Figure S7), which fall into four categories.

- Category I is completely informative (homogeneity): (1) $m_{add}=100$, $\mu _{add}=3$, and locations of the $m_{add}$ SNPs perfectly match those of $m_1$ GWAS truly associated SNPs.
- Category II is partially informative: (2) $m_{add}=100$ and $\mu _{add}=1.5$; (3) $m_{add}=50$ and $\mu _{add}=3$; (4) $m_{add}=50$, $\mu _{add}=1.5$, and all $m_{add}$ SNPs coincide with (some of) the $m_1$ SNPs.
- Category III is (partially or completely) misleading: (5) $m_{add}=100$ and $\mu _{add}=3$; (6) $m_{add}=100$ and $\mu _{add}=1.5$, but in both scenarios only 50 out of the $m_{add}$ SNPs coincide with 50 of the $m_1$ SNPs. And (7) $m_{add}=100$ and $\mu _{add}=3$, but none of the $m_{add}$ SNPs coincide with the $m_1$ SNPs.
- Category IV is uninformative: (8) $m_{add}=0$ and $\mu _{add}=0$. That is, the additional information available is white noise.

For each of the eight scenarios, we simulated 1000 data replicates, independently of each other. For each replicate, we applied the four data-integration methods that are suitable for this simulation study, namely meta-analysis, Fisher’s method, weighted *p* value, and sFDR control. Finally, we evaluated the methods using various performance measures, which we describe below.

#### Method evaluation: power and relative efficiency (*RE*)

We first used the Bonferroni corrected threshold to declare significance, *p* value $< 0.05/10000=5\times 10^{-6}$. Let $P_{rep_t}$ be the number of positives for each of the 1000 simulation replicates after data-integration, we defined power as

$$\begin{aligned} Power_{rep_t}=\frac{P_{rep_t}}{m_1}, \end{aligned}$$

the proportion of the truly associated GWAS SNPs that were found after data-integration, which is similar to $Recall_t$ defined earlier in simulation study design I.

As the Bonferroni approach can be conservative, we explored two alternative decision rules: fixed-region and fixed-FDR rejections. The fixed-region rule rejected the top *k* SNPs (e.g. $k =100$), while the fixed-FDR rule rejected SNPs by controlling FDR at $\gamma \%$ level (e.g. $\gamma \%=5\%$). For each rejection rule, we then calculated power of a method as described above.

Finally, we considered ranked-based relative efficiency as a performance measure. To this end, we first ranked all the truly associated $m_1$ SNPs based on the GWAS summary statistics alone, denoted as $R_{baseline}$. After data-integration, we use $R_{method}$ to denote the ranks of the $m_1$ SNPs based on their $Z^{meta}$, $Z^{Fisher}$, $p_{weighted}$, and $p_{sFDR}$ values. Finally, after averaging $R_{baseline}$ and $R_{method}$ across the $m_1$ SNPs and across the 1000 simulated replicates, we defined relative efficiency as

$$\begin{aligned} RE_{method}=1-\frac{\overline{R}_{method}}{\overline{R}_{baseline}}. \end{aligned}$$

A positive $RE_{method}$ value means the truly associated $m_1$ SNPs are ranked higher, on average, after data-integration using the method; a $RE_{method}$ value of zero means that the date-integration method did not improve performance; and a negative $RE_{method}$ value suggests that the data-integration effort was counter-productive.

### Integrating UK Biobank GWAS summary statistics with functional annotations

We studied all 1132 UK Biobank traits for which the confidence for their heritability inference was considered “medium” or “high” by Nealelab. For each trait, we analyzed the 7,895,174 autosomal SNPs that are bi-allelic and common (MAF $>5\%$), integrating their GWAS summary statistics with the CADD (or Eigen) meta-scores using the weighted *p* value and sFDR methods. We excluded meta-analysis and Fisher’s method from the analysis here, because severe robustness issues (to partially informative, uninformative, or misleading $z_{i,add}$) were found in simulation studies; see results for details. For comparison, we also applied FINDOR using the set of 75 publicly available annotations recommended by the authors; see Web Resources.

To summarize the application results, we first counted the numbers of independent, significant loci (at the $5\times 10^{-8}$ level) identified before and after data-integration for each of the 1132 traits, stratified by the four trait categories (nonsig, nomimal, z4, and z7). We then calculated *Recall*, the proportion of the initial GWAS findings that were retained after data-integration, as previously defined for the simulation studies. Finally, we used *New Discoveries* to represent the number of new genome-wide significant findings at the $5\times 10^{-8}$ level.

## Supplementary Information

Supplementary Tables.

Supplementary Figures.

## Data Availability

All codes used for data analyses and simulation studies are open-resource at https://github.com/jianhuig/Integrate-gwas//#readme. Data used in this work are GWAS summary statistics and functional annotation scores, which are all publicly available: UK Biobank GWAS summary statistics from Nealelab, http://www.nealelab.is/uk-biobank. UK Biobank SNP-heritbability estimates from Nealelab, https://nealelab.github.io/UKBB_ldsc/. The 1000 Genome Projects, http://tcag.ca/tools/1000genomes.html. CADD(v1.6) , https://cadd.gs.washington.edu. Eigen (v1.0) through ANNOVAR software, http://annovar.openbioinformatics.org/en/latest/user-guide/filter/#eigen-score-annotations. FINDOR, https://github.com/gkichaev/FINDOR. Nealab co-heritability browse, https://ukbb-rg.hail.is/rg_browser/.

## References

[1] Visscher PM (2017). 10 years of GWAS discovery: Biology, function, and translation. Am. J. Hum. Genet..

[2] Spencer CCA, Su Z, Donnelly P, Marchini J (2009). Designing genome-wide association studies: Sample size, power, imputation, and the choice of genotyping chip. PLoS Genet..

[3] Holland D (2016). Estimating effect sizes and expected replication probabilities from GWAS summary statistics. Front. Genet..

[4] Eskin E (2008). Increasing power in association studies by using linkage disequilibrium structure and molecular function as prior information. Genome Res..

[5] Yoo YJ, Bull SB, Paterson AD, Waggott D, Sun L (2010). Were genome-wide linkage studies a waste of time? Exploiting candidate regions within genome-wide association studies. Genet. Epidemiol..

[6] Cantor RM, Lange K, Sinsheimer JS (2010). Prioritizing GWAS results: A review of statistical methods and recommendations for their application. Am. J. Hum. Genet..

[7] Kim J, Bai Y, Pan W (2015). An adaptive association test for multiple phenotypes with GWAS summary statistics. Genet. Epidemiol..

[8] Zhu X, Stephens M (2017). Bayesian large-scale multiple regression with summary statistics from genome-wide association studies. Ann. Appl. Stat..

[9] Turley P (2018). Multi-trait analysis of genome-wide association summary statistics using MTAG. Nat. Genet..

[10] Cochran WG (1954). The combination of estimates from different experiments. Biometrics.

[11] Fisher RA (1938). Statistical Methods for Research Workers.

[12] Lin DY, Zeng D (2010). Meta-analysis of genome-wide association studies: No efficiency gain in using individual participant data. Genet. Epidemiol..

[13] Sung YJ (2014). An empirical comparison of meta-analysis and mega-analysis of individual participant data for identifying gene-environment interactions. Genet. Epidemiol..

[14] Kundaje A (2015). Integrative analysis of 111 reference human epigenomes. Nature.

[15] Dunham I (2012). An integrated encyclopedia of DNA elements in the human genome. Nature.

[16] Davydov EV (2010). Identifying a high fraction of the human genome to be under selective constraint using GERP++. PLOS Comput. Biol..

[17] Adzhubei IA (2010). A method and server for predicting damaging missense mutations. Nat. Methods.

[18] Lu Q, Powles RL, Wang Q, He BJ, Zhao H (2016). Integrative tissue-specific functional annotations in the human genome provide novel insights on many complex traits and improve signal prioritization in genome wide association studies. PLOS Genet..

[19] Shihab HA (2015). An integrative approach to predicting the functional effects of non-coding and coding sequence variation. Bioinformatics.

[20] Lu Q (2015). A statistical framework to predict functional non-coding regions in the human genome through integrated analysis of annotation data. Sci. Rep..

[21] Ritchie GRS, Dunham I, Zeggini E, Flicek P (2014). Functional annotation of noncoding sequence variants. Nat. Methods.

[22] Kircher M (2014). A general framework for estimating the relative pathogenicity of human genetic variants. Nat. Genet..

[23] Ionita-laza I, Mccallum K, Xu B, Buxbaum J (2016). A spectral approach integrating functional genomic annotations for coding and noncoding variants. Nat. Genet..

[24] Li X (2020). Dynamic incorporation of multiple in silico functional annotations empowers rare variant association analysis of large whole-genome sequencing studies at scale. Nat. Genet..

[25] Liang J (2019). Sequencing analysis at 8p23 identifies multiple rare variants in DLC1 associated with sleep-related oxyhemoglobin saturation level. Am. J. Hum. Genet..

[26] Pereira SV-N, Ribeiro JD, Ribeiro AF, Bertuzzo CS, Marson FAL (2019). Novel, rare and common pathogenic variants in the CFTR gene screened by high-throughput sequencing technology and predicted by in silico tools. Sci. Rep..

[27] Genovese CR, Roeder K, Wasserman L (2006). False discovery control with p-value weighting. Biometrika.

[28] Sun L, Craiu RV, Paterson AD, Bull SB (2006). Stratified false discovery control for large-scale hypothesis testing with application to genome-wide association studies. Genet. Epidemiol..

[29] Benjamini Y, Hochberg Y (1995). Controlling the false discovery rate: A practical and powerful approach to multiple testing. J. R. Stat. Soc. Ser. B (Methodol.).

[30] Roeder K, Bacanu S-A, Wasserman L, Devlin B (2006). Using linkage genome scans to improve power of association in genome scans. Am. J. Hum. Genet..

[31] Li L (2013). Using eQTL weights to improve power for genome-wide association studies: a genetic study of childhood asthma. Front. Genet..

[32] Keel BN (2020). Using SNP weights derived from gene expression modules to improve GWAS power for feed efficiency in pigs. Front. Genet..

[33] Andreassen OA (2013). Improved detection of common variants associated with schizophrenia and bipolar disorder using pleiotropy-informed conditional false discovery rate. PLoS Genet..

[34] Sudlow C (2015). UK Biobank: An open access resource for identifying the causes of a wide range of complex diseases of middle and old age. PLoS Med..

[35] Kichaev G (2019). Leveraging polygenic functional enrichment to improve GWAS power. Am. J. Hum. Genet..

[36] Finucane HK (2015). Partitioning heritability by functional annotation using genome-wide association summary statistics. Nat. Genet..

[37] Gazal S (2017). Linkage disequilibrium-dependent architecture of human complex traits shows action of negative selection. Nat. Genet..

[38] Bulik-Sullivan BK (2015). LD Score regression distinguishes confounding from polygenicity in genome-wide association studies. Nat. Genet..

[39] Visscher PM (2017). 10 years of GWAS discovery: Biology, function, and translation. Am. J. Hum. Genet..

[40] Li Y (2020). Integration of GWAS summary statistics and gene expression reveals target cell types underlying kidney function traits. J. Am. Soc. Nephrol..

[41] Uffelmann E (2021). Genome-wide association studies. Nat. Rev. Methods Primers.

[42] Thompson SG (1994). Why sources of heterogeneity in meta-analysis should be investigated. BMJ Br. Med. J..

[43] Begum F, Ghosh D, Tseng GC, Feingold E (2012). Comprehensive literature review and statistical considerations for GWAS meta-analysis. Nucleic Acids Res..

[44] Onengut-Gumuscu S (2015). Fine mapping of type 1 diabetes susceptibility loci and evidence for colocalization of causal variants with lymphoid gene enhancers. Nat. Genet..

[45] Van Hout CV (2020). Exome sequencing and characterization of 49,960 individuals in the UK Biobank. Nature.

[46] Hedges LV, Vevea JL (1998). Fixed- and random-effects models in meta-analysis. Psychol. Methods.

[47] Storey JD (2002). A direct approach to false discovery rates. J. R. Stat. Soc. Ser. B (Stat. Methodol.).

[48] Storey JD, Tibshirani R (2003). Statistical significance for genomewide studies. Proc. Natl. Acad. Sci. USA.

[49] Chen B, Craiu RV, Strug LJ, Sun L (2021). The x factor: A robust and powerful approach to x-chromosome-inclusive whole-genome association studies. Genet. Epidemiol..

[50] Derkach A, Lawless JF, Sun L (2014). Pooled association tests for rare genetic variants: A review and some new results. Stat. Sci..

[51] Rentzsch P, Witten D, Cooper GM, Shendure J, Kircher M (2019). CADD: Predicting the deleteriousness of variants throughout the human genome. Nucleic Acids Res..

[52] Wang K, Li M, Hakonarson H (2010). ANNOVAR: Functional annotation of genetic variants from high-throughput sequencing data. Nucleic Acids Res..

[53] Auton A (2015). A global reference for human genetic variation. Nature.

[54] Roslin NM, Weili L, Paterson AD, Strug LJ (2016). Quality control analysis of the 1000 Genomes Project Omni2.5 genotypes. bioRxiv.

[55] Dudbridge F, Gusnanto A (2008). Estimation of significance thresholds for genomewide association scans. Genet. Epidemiol..

[56] Purcell S (2007). PLINK: A tool set for whole-genome association and population-based linkage analyses. Am. J. Hum. Genet..

