# Supplementary material for: Integrating variant functional annotation scores have varied abilities to improve power of genome-wide association studies
Source: Sci Rep. 2022 Jun 24;12:10720. doi: 10.1038/s41598-022-14924-1 (PMC9232605; doi:10.1038/s41598-022-14924-1)
Supplement: Supplementary file 1 — Supplementary Tables. [file 41598_2022_14924_MOESM1_ESM.pdf]

Method	k_0	k_1	k_2	k_3	FWER
Baseline	47522	2409	68	1	0.04956
Meta-analysis	47617	2332	49	2	0.04766
Fisher's method	48172	1790	38	0	0.03656
Weighted p-value	47495	2443	62	0	0.05010
stratified FDR	47629	2303	67	1	0.04742
FINDOR	47316	2618	64	2	0.05368

Table S1. **Results of simulation study design I that leverages the real functional scores combined with GWAS summary statistics simulated under the null of no association.** Breakdown of the number of false positive findings for the $k = 50,000$ simulated replicates using the Bonferroni threshold of 1.2×10^{-7}. Among the $k = 50,000$ replicates, $k_n, n = 0, \ldots, 3$ represent number of replicates have n false findings. Assuming the true FWER is 0.05, we expect the estimate obtained from the 50,000 independent simulation replicates to have a standard error of $\sqrt{0.05 \times 0.95/50000} \approx 0.001$. Thus, a method with a FWER estimate outside [0.047, 0.053] can be considered inaccurate.

	Methods					
	GWAS alone	Data-integration of GWAS and Functional Annotation Score				
		75 individual scores	CADD meta-score		Eigen meta-score	
	UK Biobank GWAS by the Nealelab unless specified other wise[1]	FINDOR	Weighted p-value	Stratified FDR	Weighted p-value	Stratified FDR
(A). Simulation Design I: Simulated Null GWAS with Real Annotation Scores						
**Empirical FWER**[2]	0.0496	0.0537	0.0501	0.0474	0.0489	0.0442
(B). Simulation Design II: Real UK Biobank GWAS with Permuted Annotation Scores						
**Median of** ***Recall$_t$***[3] **[Q1, Q3]**	NA	98.46% [95.09%, 100%]	100% [95.87%, 100%]	100% [100%, 100%]	100% [96.38%,, 100%]	100% [100%, 100%]
**Median of** ***Precision$_t$***[4] **[Q1, Q3]**	NA	97.51% [87.56%, 100%]	96.96% [89.29%, 100%]	100% [100%, 100%]	97.53% [92.36%, 100%]	100% [100%, 100%]
(C). Application Study: Integrating UK Biobank GWAS summary data with Annotation Scores						
**Median of # of significant loci**[5] **[Q1, Q3]**	GWAS alone	FINDOR	Weighted p-value	Stratified FDR	Weighted p-value	Stratified FDR
overall (1,132 traits)	2 [0, 18]	2 [0, 21]	2 [0, 20]	2 [0, 18]	2 [0, 19.25]	2 [0, 18]
nonsig (182 traits)	0 [0, 0]	0 [0, 0]	0 [0, 0]	0 [0, 0]	0 [0, 0]	0 [0, 0]
nominal (277 traits)	0 [0, 1]	0 [0, 1]	0 [0, 1]	0 [0, 1]	0 [0, 1]	0 [0, 1]
z4 (235 traits)	1 [0, 4.5]	2 [0, 5.5]	1 [0, 5]	1 [0, 4.5]	1 [0, 4.5]	1 [0, 4.5]
z7 (438 traits)	26.5 [5, 245.8]	31.5 [6, 255]	27 [5, 255]	26.5 [5, 245.8]	27 [5, 251.5]	26 [5, 246.2]
**Median of** ***Recall$_t$***[6] **[Q1, Q3]**						
overall (337 traits)	NA	97.56% [96.15%, 98.73%]	96.88% [95.79%, 98.27%]	100% [99.91%, 100%]	97.43% [96.35%, 98.40%]	100% [99.85%, 100%]
nosig (4 traits)	NA	100% [98.21%, 100%]	100% [99%, 100%]	100% [98.21%, 100%]	100% [99%, 100%]	100% [100%, 100%]
nomial (11 traits)	NA	96% [90.04%, 98.92%]	97.84% [95.31%, 100%]	100% [100%, 100%]	98.85% [96.44%, 97.06%]	100% [100%, 100%]
z4 (42 traits)	NA	97.30% [94.12%, 98.35%]	97.15% [95.64%, 100%]	100% [100%, 100%]	97.53% [96.36%, 99.75%]	100% [100%, 100%]
z7 (280 traits)	NA	97.61% [96.47%, 98.69%]	96.83% [95.81%, 97.99%]	100% [99.89%, 100%]	97.37% [96.36%, 97.06%]	100% [98.36%, 100%]
**Median of # of** ***New Discoveries*** **[Q1, Q3]**						
overall (1,132 traits)	NA	0 [0, 3]	0 [0, 2]	0 [0, 0]	0 [0, 1]	0 [0, 0]
nonsig (182 traits)	NA	0 [0, 0]	0 [0, 0]	0 [0, 0]	0 [0, 0]	0 [0, 0]
nominal (277 traits)	NA	0 [0, 0]	0 [0, 0]	0 [0, 0]	0 [0, 0]	0 [0, 0]
z4 (235 traits)	NA	0 [0, 1]	0 [0, 1]	0 [0, 0]	0 [0, 1]	0 [0, 0]
z7 (438 traits)	NA	4 [1, 18.75]	3 [1, 13.75]	0 [0, 0]	2 [0, 11]	0 [0, 1]
**# of traits with >0, 5, 10 significant loci**						
overall (1,132 traits)	772, 402, 337	738, 420, 353	746, 408, 346	717, 403, 337	701, 405, 346	715, 402, 337
nonsig (182 traits)	36, 5, 4	39, 5, 5	40, 5, 4	36, 5, 4	41, 5, 4	36, 5, 4
nominal (277 traits)	110, 18,11	102, 21, 11	113, 21, 11	109, 18, 11	110, 20,12	107, 18, 11
z4 (235 traits)	160, 55, 42	169, 59, 45	172, 56, 43	157, 56, 42	169, 56, 42	158, 56, 42
z7 (438 traits)	416, 324, 280	428, 335, 292	421, 326, 288	415, 324, 280	421, 324, 288	414, 324, 280
**# of traits with >0, 5, 10** ***New Discoveries***						
overall (1,132 traits)	NA	553, 227, 165	472, 180, 139	89, 0, 0	422, 154, 119	144, 0, 0
nonsig (182 traits)	NA	20, 0, 0	15, 0, 0	0, 0, 0	9, 0, 0	1, 0, 0
nominal (277 traits)	NA	50, 3, 1	37, 1, 0	3, 0, 0	30, 1, 0	4, 0, 0
z4 (235 traits)	NA	103, 22, 11	77, 16, 10	9, 0, 0	75, 11, 8	14, 0, 0
z7 (438 traits)	NA	380, 202, 153	343, 163, 130	77, 0, 0	308, 142, 111	125, 0, 0

Table S2. **A summary of results from the simulation and application studies**

[1] GWAS summary statistics of 1,132 phenotypes from the UK Biobank data (Web Resources). These 1,132 traits were rated as with medium to high confidence for their heritability estimates by Nealelab and fall into four categories: nonsig (182 traits; SNP-heritability testing $p > 0.5$), nominal (277 traits; $p < 0.05$), z4 (235 traits; $p < 3.17 \times 10^{-5}$), and z7 (438 traits; $p < 1.28 \times 10^{-12}$).

[2] FWER estimated from 50,000 simulated replicates; a method with a FWER estimate outside [0.047, 0.053] can be considered inaccurate. See Table S1 for a detailed account of the numbers of replicates that have at least one, two or three false findings for each of the methods.

[3] $Recall_t = TP_t/m_{1,t}$, where $m_{1,t}$ is the number of genome-wide significant loci at 5×10^{-8} prior to data-integration for trait t, and TP_t is the number of true positives after data-integration. Recall was calculated only for 723 traits with $m_{1,t} > 0$. FINDOR was not evaluated here because the validity of running LDSC on permuted annotations is not clear. See Figure 2 for the distributions of $Recall_t$.

[4] $Precision_t = TP_t/P_t$, where P_t is the total number of positives at 5×10^{-8} after data-integration. See Figure 2 for the distributions of $Preciation_t$.

[5] The number of independent, significant loci detected at 5×10^{-8} before and after data-integration. See Figures 4 and S19 for the corresponding box-plots, and see Figure S21 for the overlap between the findings.

[6] Only for traits with greater than 10 significant loci ($m_{1,t} > 10$) for which the recall estimation is stable. See Figure 6(A) for the corresponding box-plots, and Figure 6(B) for results for the remaining 795 traits with $m_{1,t} \leq 10$. For box-plots of *Recall* when $m_{1,t} > 5$ see Figure S26, and without the $m_{1,t}$ restriction see Figure S25.
