# Supplementary material for: Integrating variant functional annotation scores have varied abilities to improve power of genome-wide association studies
Source: Sci Rep. 2022 Jun 24;12:10720. doi: 10.1038/s41598-022-14924-1 (PMC9232605; doi:10.1038/s41598-022-14924-1)
Supplement: Supplementary file 2 — Supplementary Figures. [file 41598_2022_14924_MOESM2_ESM.pdf]

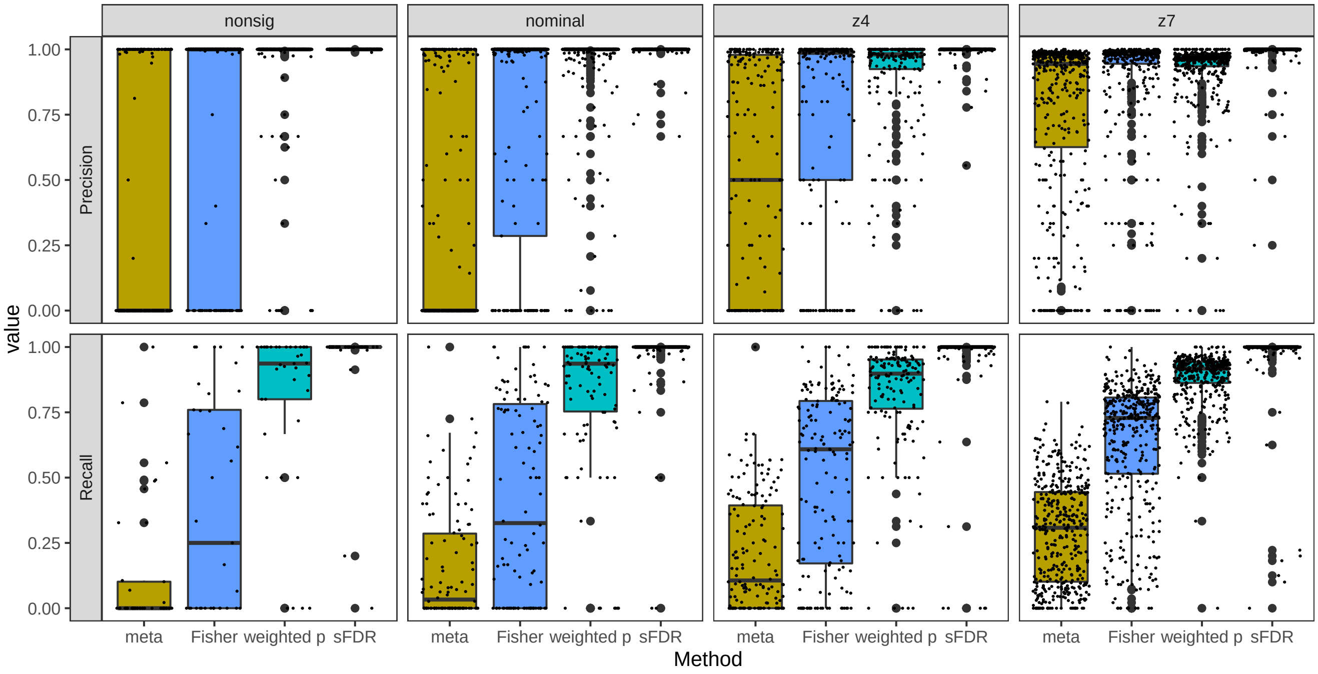

Figure S1. The trait-type-stratified *Recall* and *Precision* rates obtained from simulation study design II. The study integrated the 1,132 UK Biobank GWAS summary statistics with *permuted* CADD functional meta-scores, using meta-analysis, Fisher's method, the weighted p-value approach, and the stratified FDR control. The 1,132 traits fall into four categories by Nealelab: nonsig (182 traits; $p > 0.05$), nominal (277 traits; $p < 0.05$), z4 (235 traits; $p < 3.17 \times 10^{-5}$), and z7 (438 traits; $p < 1.28 \times 10 \times -12$). $Recall_t = TP_t/m_{1,t}$ and $Precision_t = 1 - FDR_t = TP_t/P_t$, where $m_{1,t}$ is the number of genome-wide significant independent loci prior to data-integration for trait t, and P_t and TP_t are the numbers of positives and true positives after data-integration.

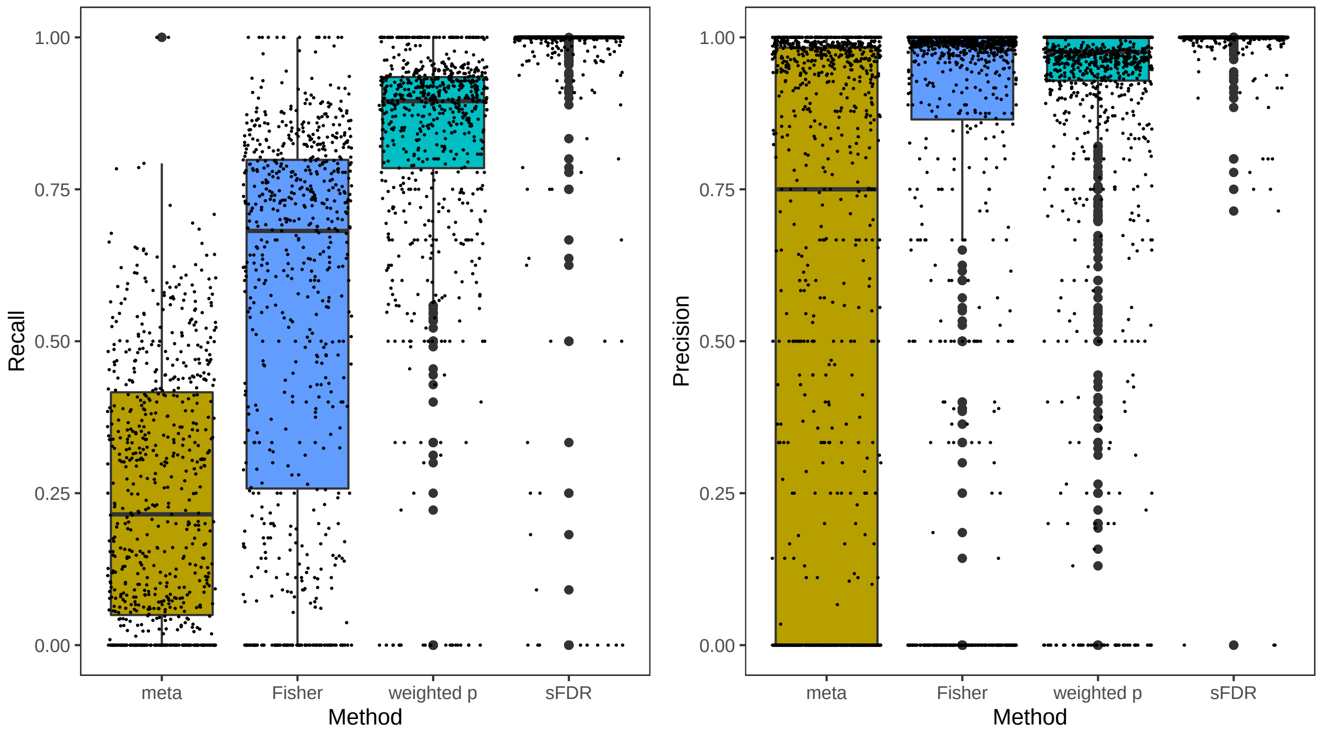

Figure S2. **SNP results for the *Recall* and *Precision* rates obtained from simulation study design II.** The study integrated the 1,132 UK Biobank GWAS summary statistics with *permuted* CADD functional meta-scores, using meta-analysis, Fisher's method, the weighted p-value approach, and the stratified FDR control. $Recall_t = TP_t/m_{1,t}$ and $Precision_t = 1 - FDR_t = TP_t/P_t$, where $m_{1,t}$ is the number of genome-wide significant SNPs prior to data-integration for trait t, and P_t and TP_t are the numbers of positives and true positives after data-integration.

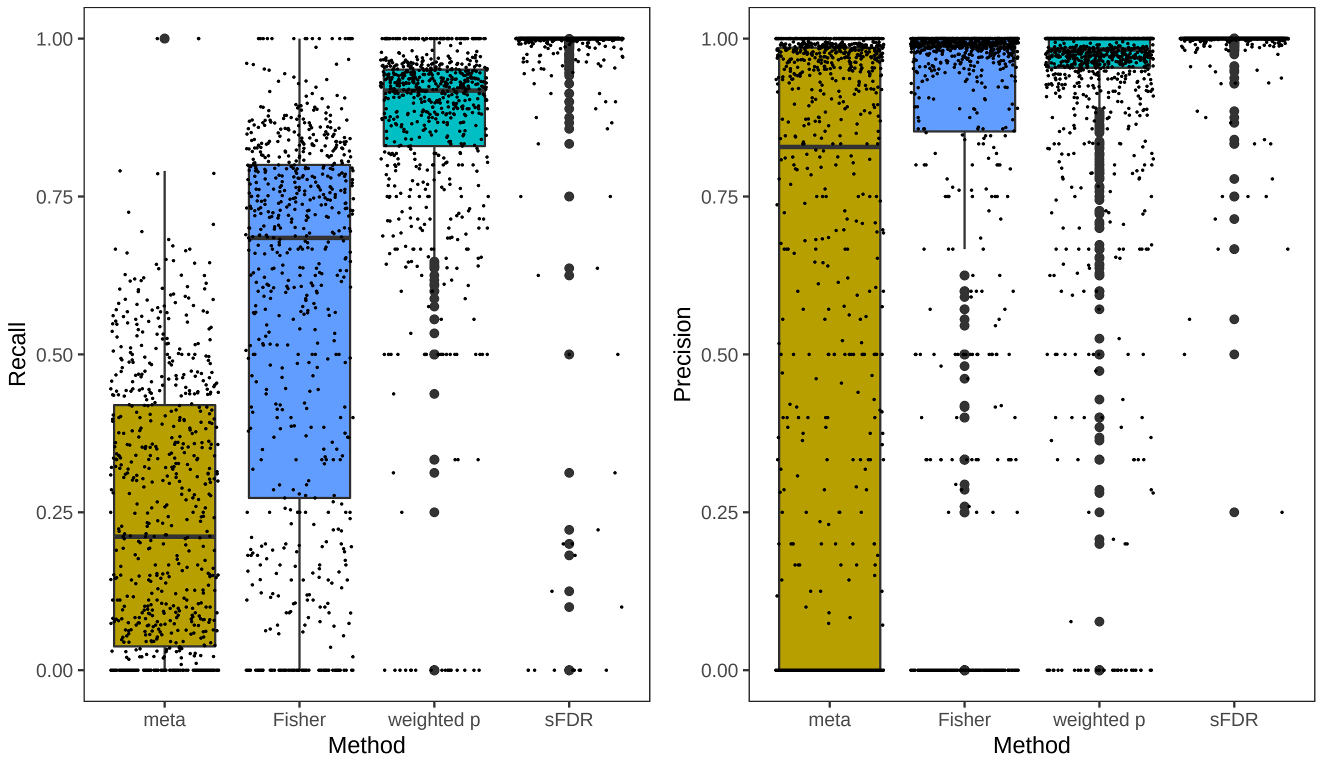

Figure S3. Eigen results for the *Recall* and *Precision* rates obtained from simulation study design II. The study integrated the 1,132 UK Biobank GWAS summary statistics with *permuted* Eigen functional meta-scores, using meta-analysis, Fisher's method, the weighted p-value approach, and the stratified FDR control. $Recall_t = TP_t/m_{1,t}$ and $Precision_t = 1 - FDR_t = TP_t/P_t$, where $m_{1,t}$ is the number of genome-wide significant independent loci prior to data-integration for trait t, and P_t and TP_t are the numbers of positives and true positives after data-integration.

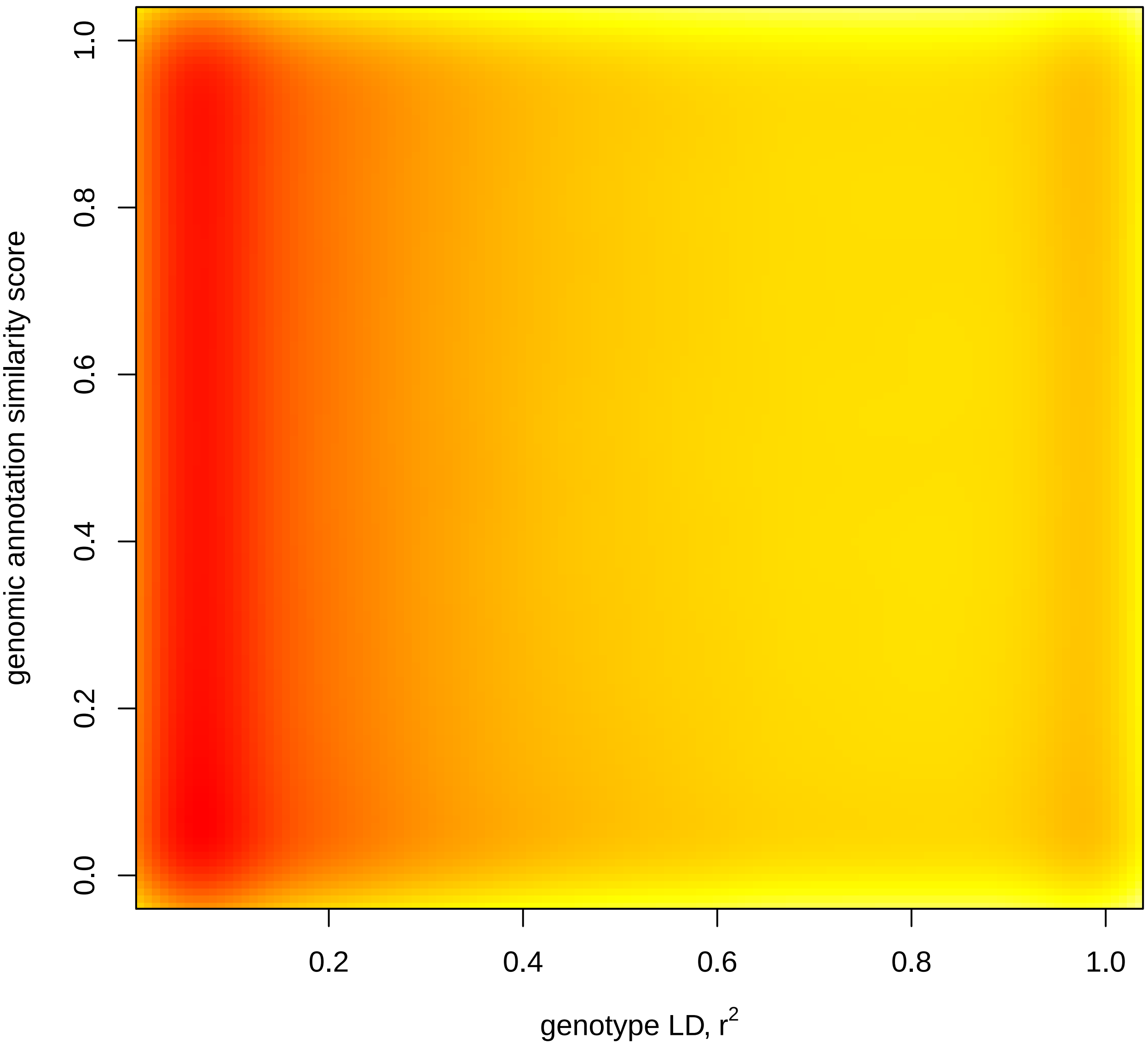

Figure S4. Contrast between $s^2_{i,j}$, a pair-wise similarity measure of annotation scores of SNPs i and j, and $r^2_{i,j}$, the linkage disequilibrium measure of genotype correlation, for the common bi-allelic autosomal SNPs of the UK Biobank data. Annotation similarity measure is defined as $s^2_{i,j} = 1 - |CADD_i - CADD_j|/(CADD_i + CADD_j)$, which is bounded between 0 and 1, where 1 means two scores are identical whereas a value close to 0 suggests a lack of similarity. The pair-wise LD $r^2_{i,j}$ values were calculated for all possible pairs within $1MB$ on each chromosome using emeraLD[1]. Area with darker color (red) represents higher number of points are observed in that region.

(a) Chr 1: 231218651-231322749

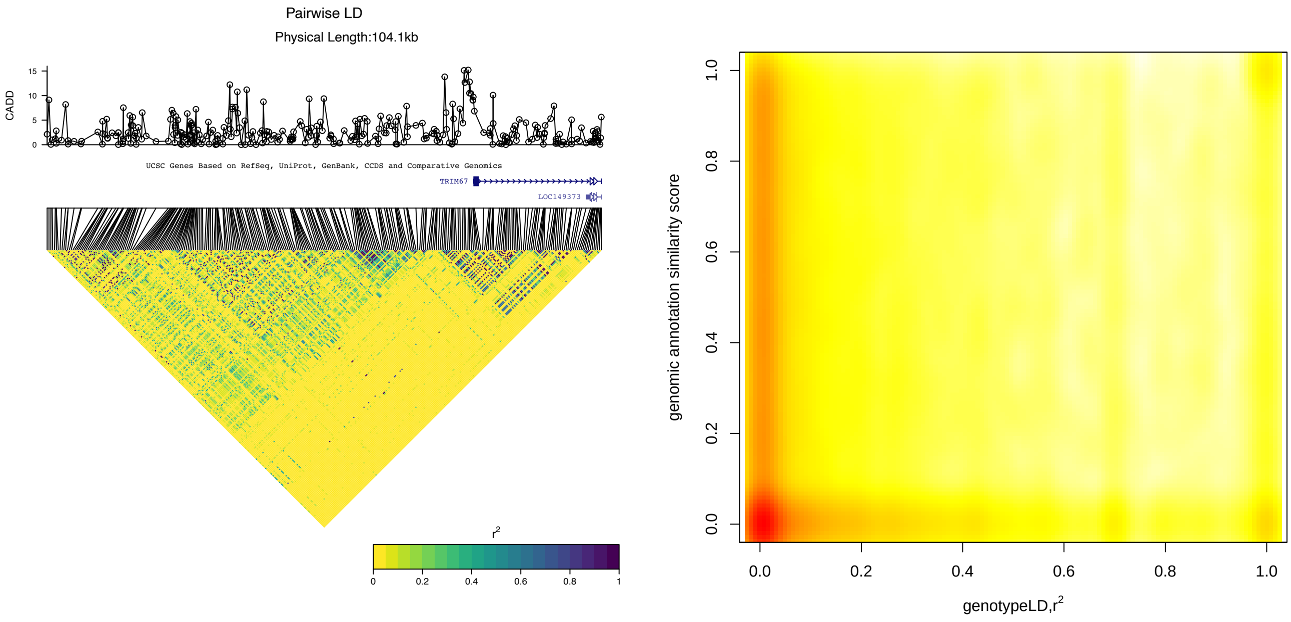

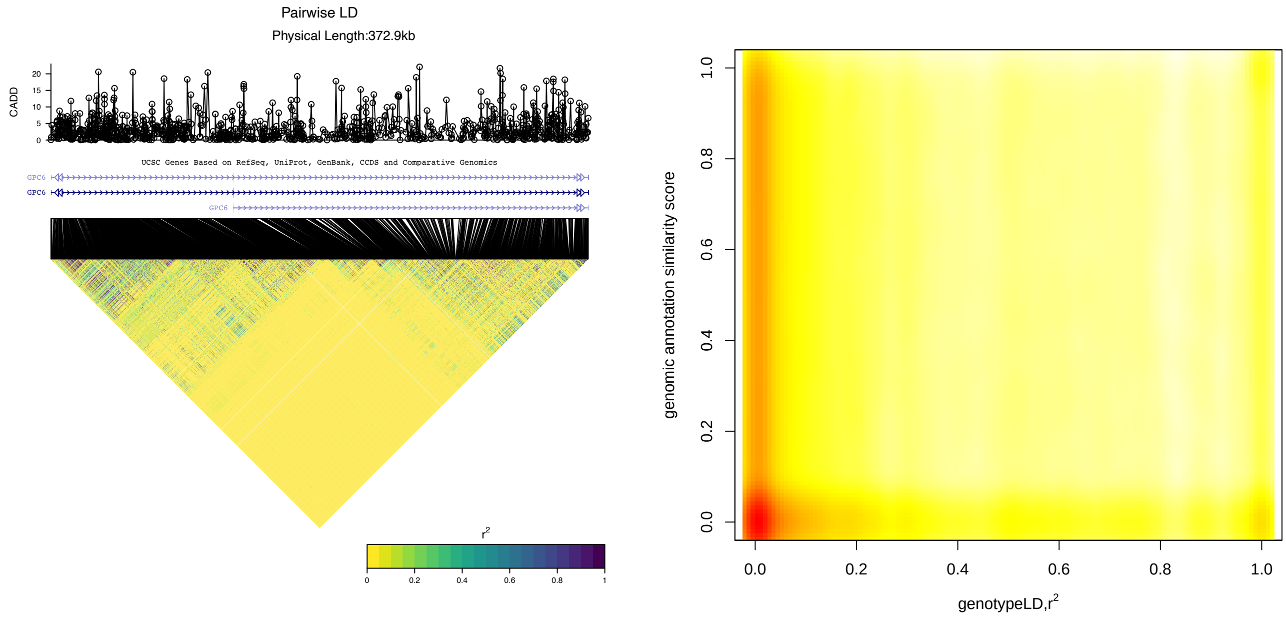

(b) Chr 13: 94071151-94444092

Figure S5. For two randomly selected regions, Left: the CADD scores and the standard pair-wise linkage disequilibrium (LD) plot along with gene information. Right: contrast between $s^2_{i,j}$, a pair-wise similarity measure of annotation scores of SNPs i and j, and $r^2_{i,j}$, the LD measure of genotype correlation. Annotation similarity measure is defined as $s^2_{i,j} = 1 - |CADD_i - CADD_j|/(CADD_i + CADD_j)$, which is bounded between 0 and 1, where 1 means two scores are identical whereas a value close to 0 suggests a lack of similarity. Area with darker color (red) represents higher number of points are observed in that region.

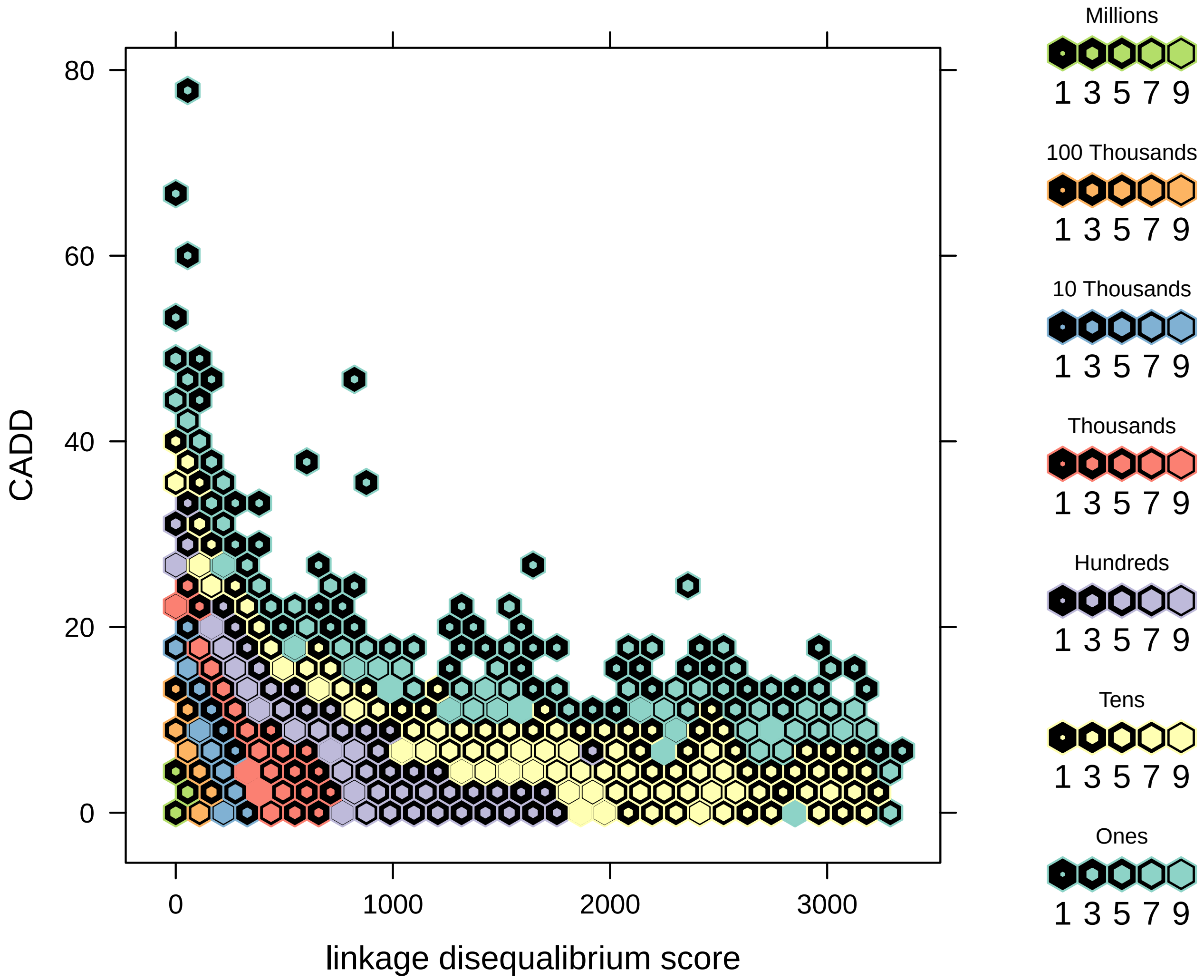

Figure S6. **Contrast between CADD meta-score and LD score for the 7,895,174 common, bi-allelic autosomal SNPs of the UK Biobank data.** For each variant i, the plot shows its CADD meta-score and the sum of $r_{i,j}^2$ across all other j variants analyzed. The color and inner size of each hexagon represents the number of SNP counts.

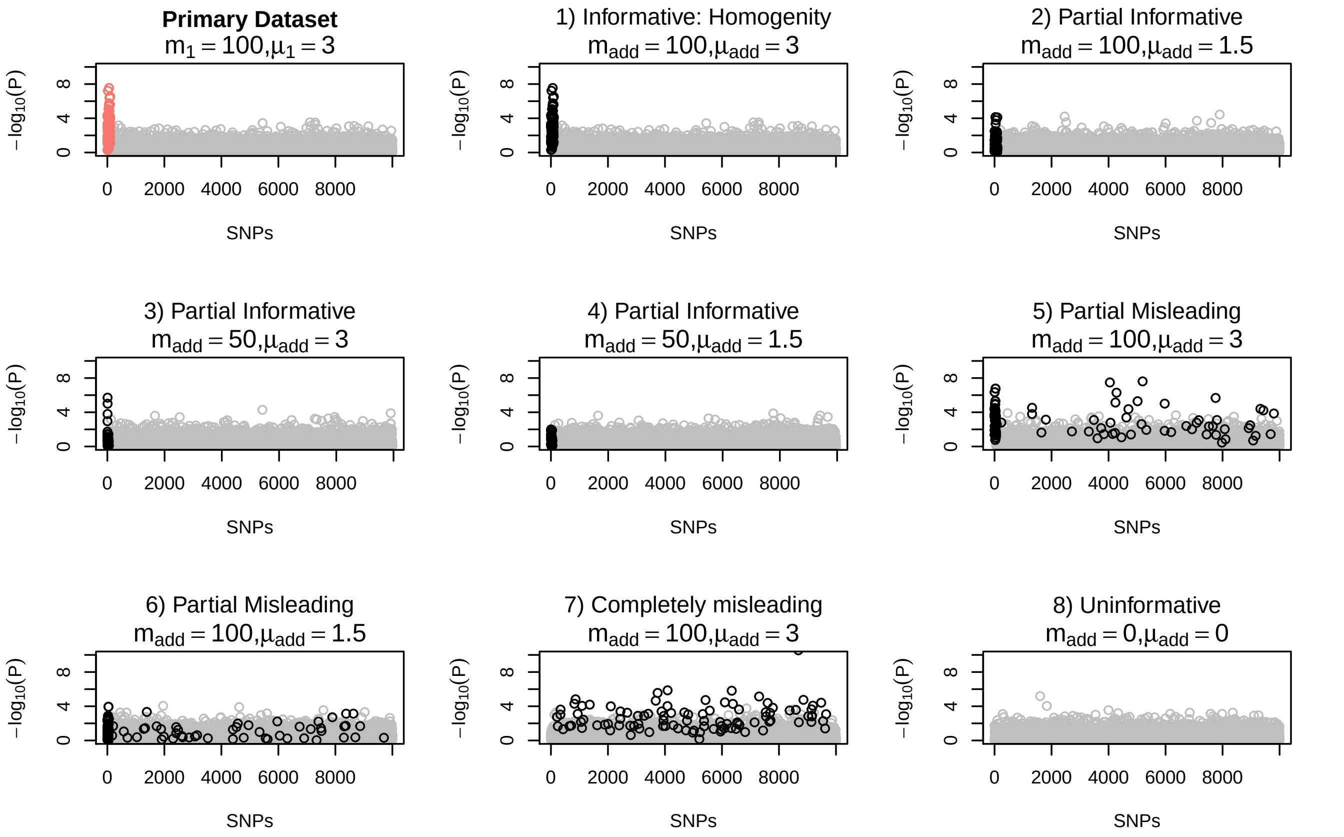

Figure S7. **Illustration of Simulation study design III, varying the informativeness of genomic information.** Among the total $m = 10,000$ SNPs, the first $m_1 = 100$ SNPs are truly associated (red circles) whose GWAS association summary statistics were drawn from $N(\mu_1 = 3, 1)$; the remaining SNPs are not associated whose association summary statistics were drawn from $N(0,1)$. The top left figure shows the Manhattan plot of of one simulation run. The other figures demonstrate eight scenarios for the additional information available for data-integration. The summary statistics of the additional information available were drawn, independently, from $N(\mu_{add}, 1)$ for m_{add} SNPs (black circles) and $N(0,1)$ for the remaining SNPs; the locations of the m_{add} SNPs may differ from those of the m_1 associated SNPs. The eight scenarios fall into four categories. Category I is completely informative (homogeneity): (1) $m_{add} = 100$, $\mu_{add} = 3$ and locations of the m_{add} SNPs perfectly match those of m_1 GWAS truly associated SNPs. Category II is partially informative: (2) $m_{add} = 100$ and $\mu_{add} = 1.5$; (3) $m_{add} = 50$ and $\mu_{add} = 3$; (4) $m_{add} = 50$, $\mu_{add} = 1.5$, and all m_{add} SNPs coincide with (some of) the m_1 SNPs. Category III is (partially or completely) misleading: (5) $m_{add} = 100$ and $\mu_{add} = 3$; (6) $m_{add} = 100$ and $\mu_{add} = 1.5$, but in both scenarios only 50 out of the m_{add} SNPs coincide with 50 of the m_1 SNPs. And (7) $m_{add} = 100$ and $\mu_{add} = 3$, but none of the m_{add} SNPs coincide with the m_1 SNPs. Category IV is uninformative: (8) $m_{add} = 0$ and $\mu_{add} = 0$. That is, the additional information available is white noise.

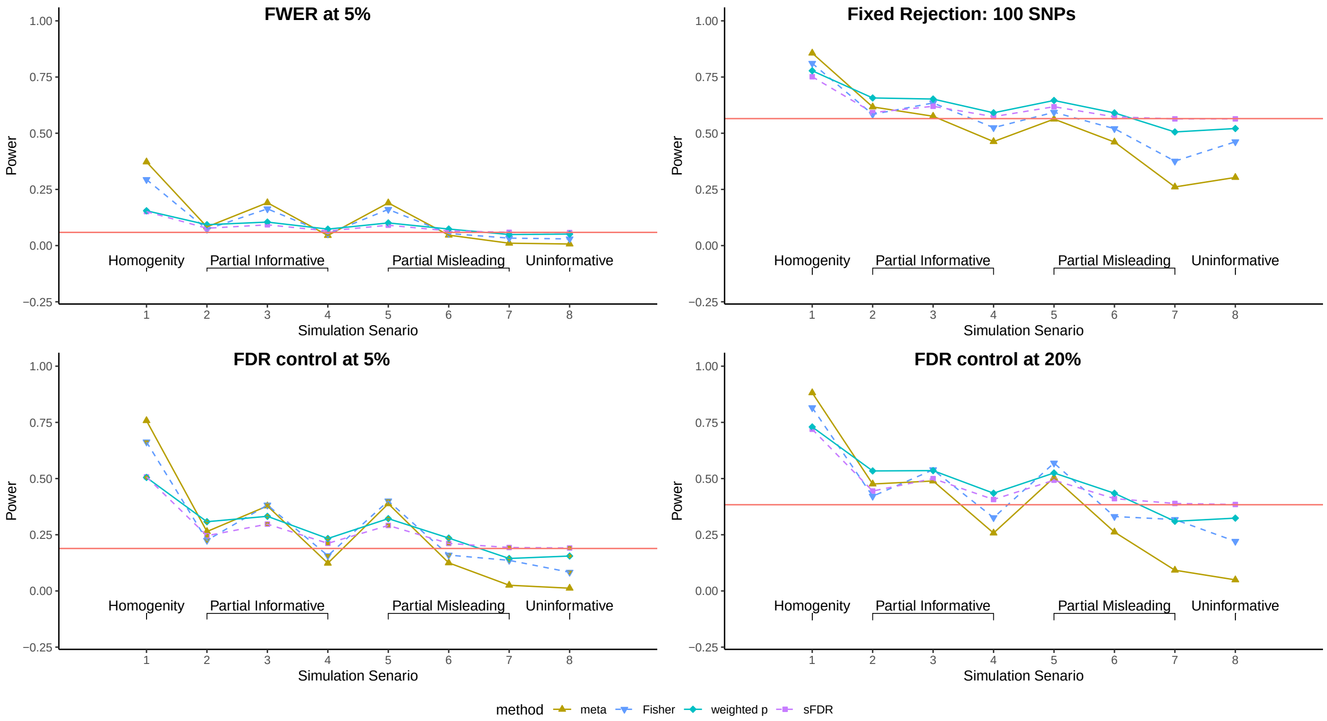

Figure S8. **Power obtained from simulation study design III.** The study integrated simulated GWAS summary statistics with simulated additional information with varying degrees of informativeness, using meta-analysis, Fisher's method, the weighted p-value approach, and the stratified FDR control. There are 10,000 independent SNPs among which 100 are truly associated whose summary statistics were drawn from $N(3,1)$; the rest from $N(0,1)$. For the additional information available for data integration, the details of the eight simulation scenarios are provided in the text and illustrated in Figure S7. Power is the proportion of true signals detected after data integration, estimated from 1,000 simulation replicates, using four different decision rules, controlling family-wise error rate at 0.05, rejecting top 100 ranked SNPs, and controlling false discovery rate at 5% or 20%. The red line represents baseline power of using GWAS data alone without data-integration.

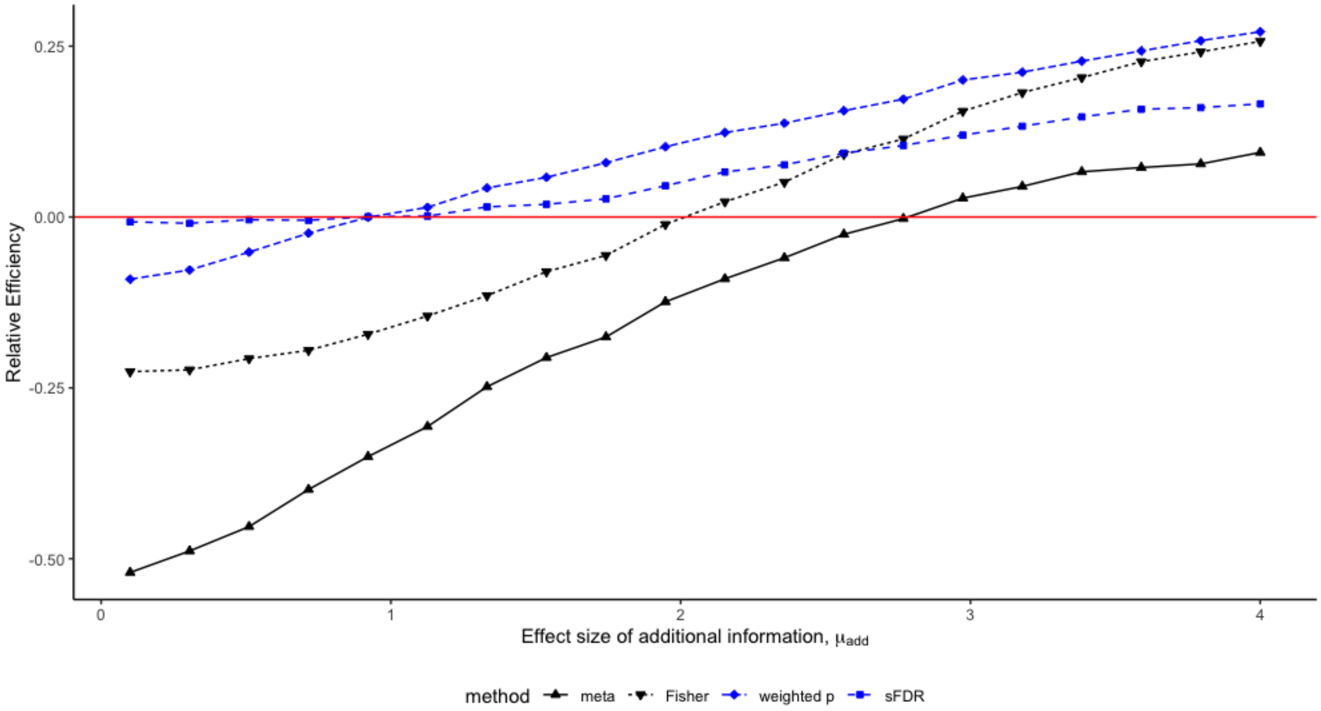

Figure S9. The relative efficiency (RE) obtained from Category II (partially informative) in simulation study design III. We let $\mu_1 = 3$ and number of additional informative SNPs $m_{add} = 50$. The effect size of additional information varied from 0.1 to 4. We assumed the total number of SNPs $m = 10,000$, among which the first $m_1 = 100$ SNPs are truly associated. The corresponding summary statistics z_i's were drawn, independently, from $N(\mu_1, 1)$ for the m_1 associated SNPs, and from $N(0,1)$ for the remaining null SNPs. We then assumed $z_{i,add}$'s as the additional information available, which were drawn, independently, from $N(\mu_{add}, 1)$ for m_{add} SNPs and $N(0,1)$ for the remaining SNPs.

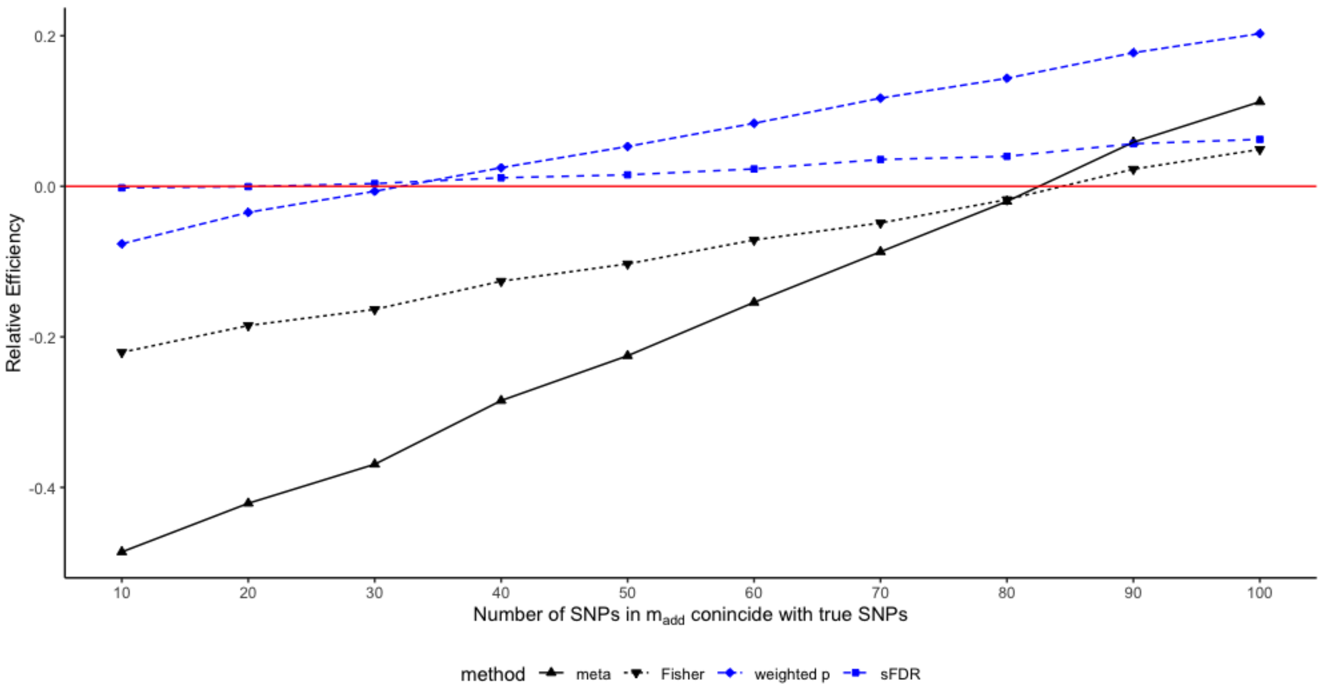

Figure S10. **The relative efficiency** (RE) **obtained from Category III (partially misleading) in simulation study design III**. We let $m_{add} = 100$, $\mu_{add} = 1.5, \mu_1 = 3$. Among 100 m_{add} SNPs, the number of SNPs conincide with true SNPs varied from 10 to 100. We assumed the total number of SNPs $m = 10,000$, among which the first $m_1 = 100$ SNPs are truly associated. The corresponding summary statistics z_i's were drawn, independently, from $N(\mu_1, 1)$ for the m_1 associated SNPs, and from $N(0,1)$ for the remaining null SNPs. We then assumed $z_{i,add}$'s as the additional information available, which were drawn, independently, from $N(\mu_{add}, 1)$ for m_{add} SNPs and $N(0,1)$ for the remaining SNPs.

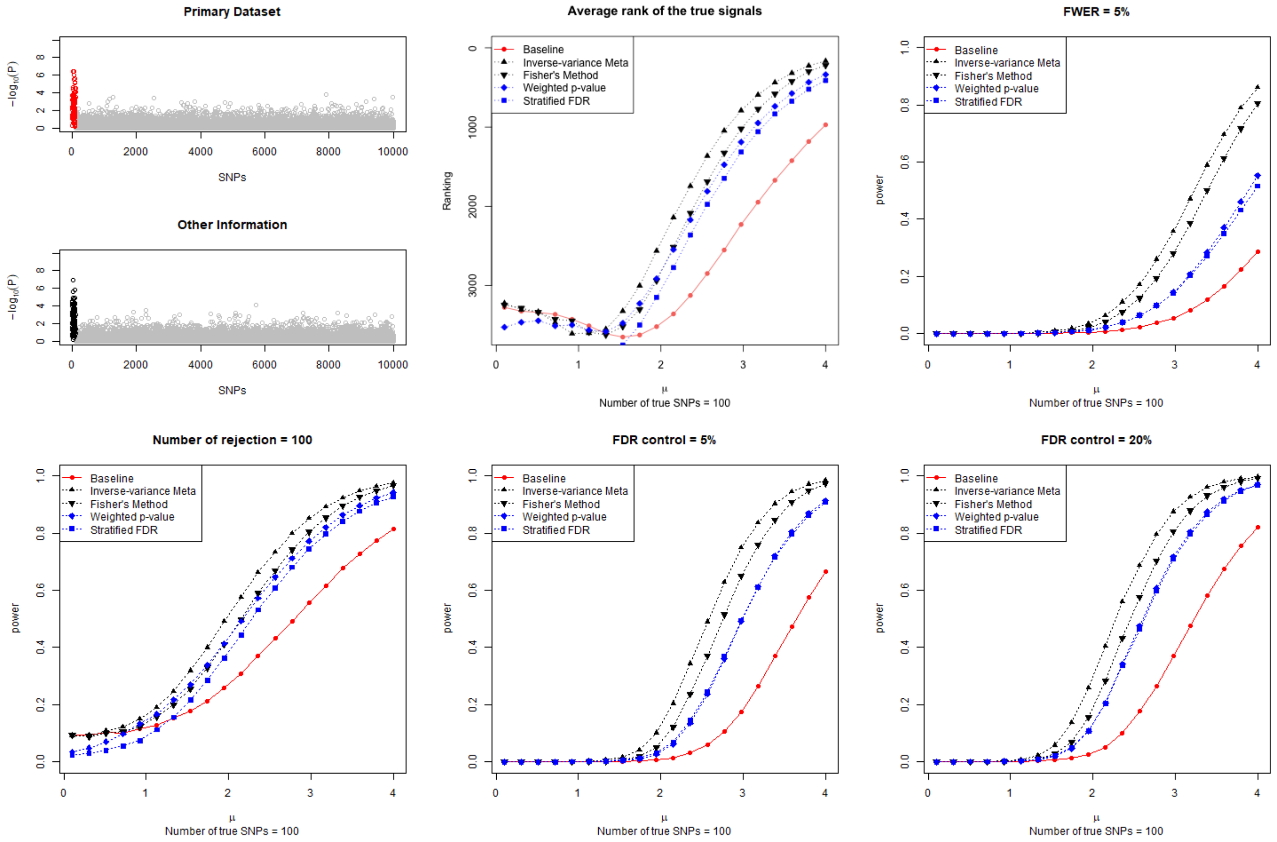

Figure S11. Results of simulation study design III for scenario (1) in Figure S7 from Category I type of informativeness, with the true effect size μ_1 for the m_1 causal SNPs varying from 0.1 to 4. Completely Informative (Homogeneity) : $m_{add} = 100$, $\mu_{add} = \mu_1$ and locations of the m_{add} SNPs perfectly match those of m_1 GWAS truly associated SNPs. We assumed the total number of SNPs $m = 10,000$, among which the first $m_1 = 100$ SNPs (in red on top-left panel) are truly associated. The corresponding summary statistics z_i's were drawn, independently, from $N(\mu_1, 1)$ for the m_1 associated SNPs, and from $N(0,1)$ for the remaining null SNPs. We then assumed $z_{i,add}$'s (in black on top-left panel) as the additional information available, which were drawn, independently, from $N(\mu_{add}, 1)$ for m_{add} SNPs and $N(0,1)$ for the remaining SNPs.

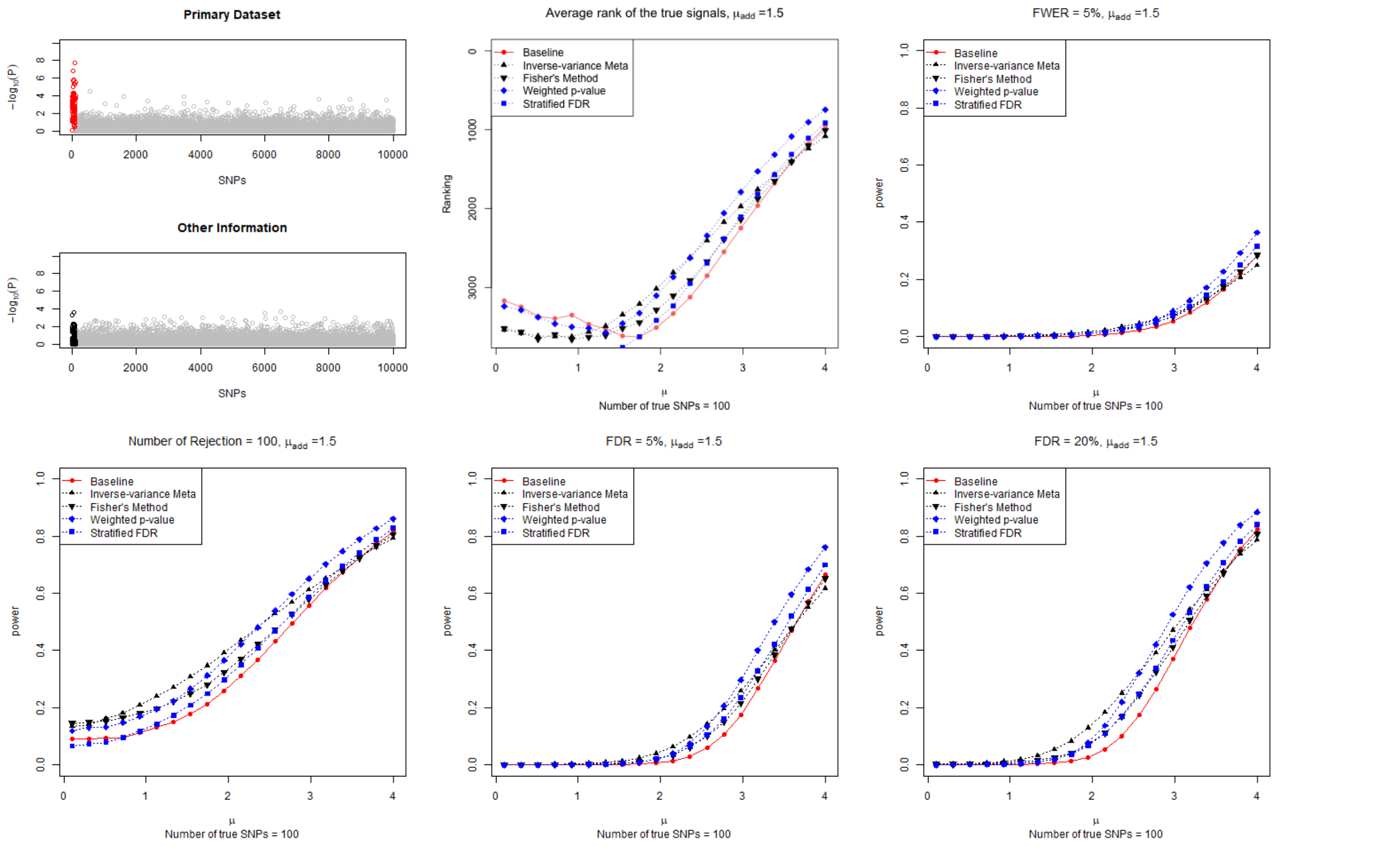

Figure S12. Results of simulation study design III for scenario (2) in Figure S7 from Category II type of informativeness, with the true effect size μ_1 for the m_1 causal SNPs varying from 0.1 to 4. Partially Informative: $m_{add} = 100$, $\mu_{add} = 1.5$, and all m_{add} SNPs coincide with (some of) the m_1 SNPs. We assumed the total number of SNPs $m = 10,000$, among which the first $m_1 = 100$ SNPs (in red on top-left panel) are truly associated. The corresponding summary statistics z_i's were drawn, independently, from $N(\mu_1, 1)$ for the m_1 associated SNPs, and from $N(0,1)$ for the remaining null SNPs. We then assumed $z_{i,add}$'s (in black on top-left panel) as the additional information available, which were drawn, independently, from $N(\mu_{add}, 1)$ for m_{add} SNPs and $N(0,1)$ for the remaining SNPs.

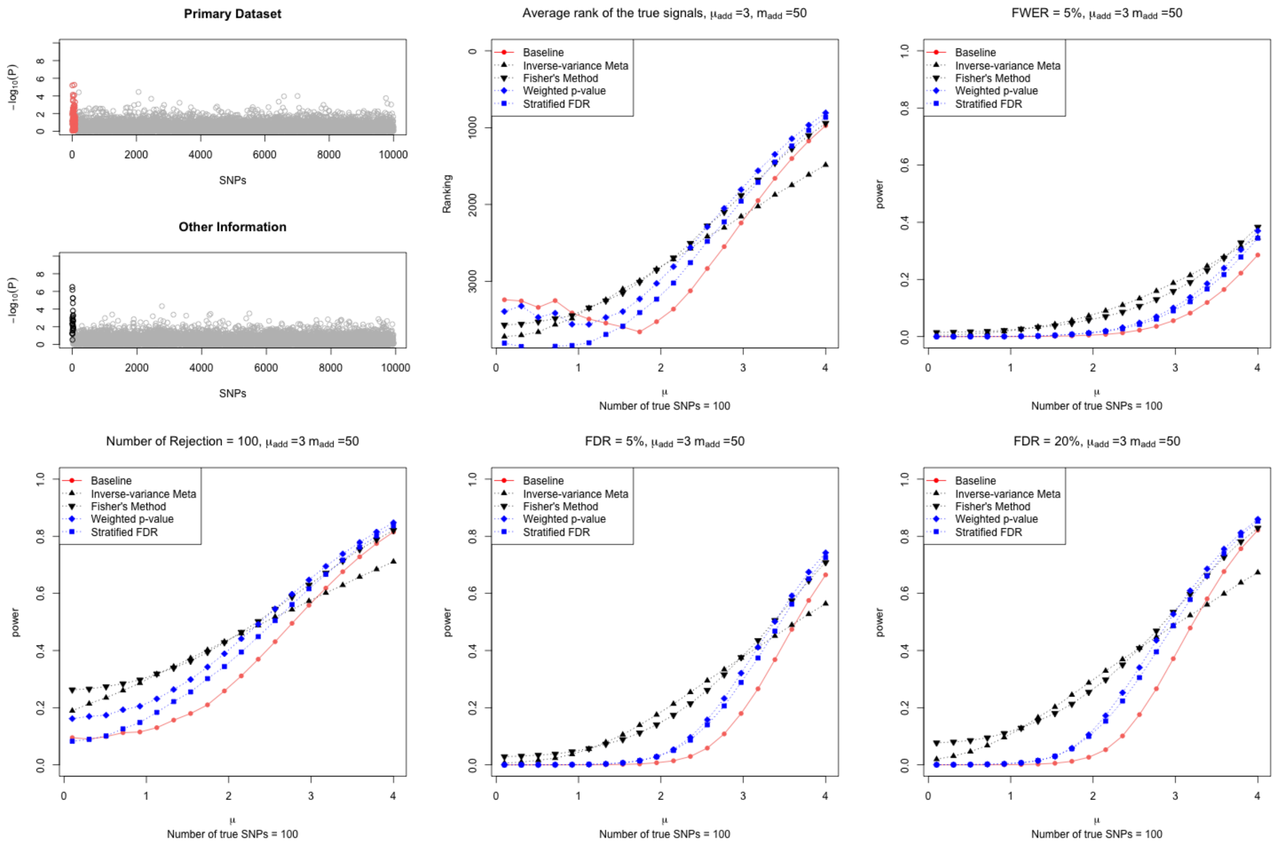

Figure S13. **Results of simulation study design III for scenario (3) in Figure S7 from Category II type of informativeness, with the true effect size μ_1 for the m_1 causal SNPs varying from 0.1 to 4.** Partially Informative: $m_{add} = 50$, $\mu_{add} = 3$, and all m_{add} SNPs coincide with (some of) the m_1 SNPs. We assumed the total number of SNPs $m = 10,000$, among which the first $m_1 = 100$ SNPs (in red on top-left panel) are truly associated. The corresponding summary statistics z_i's were drawn, independently, from $N(\mu_1, 1)$ for the m_1 associated SNPs, and from $N(0,1)$ for the remaining null SNPs. We then assumed $z_{i,add}$'s (in black on top-left panel) as the additional information available, which were drawn, independently, from $N(\mu_{add}, 1)$ for m_{add} SNPs and $N(0,1)$ for the remaining SNPs.

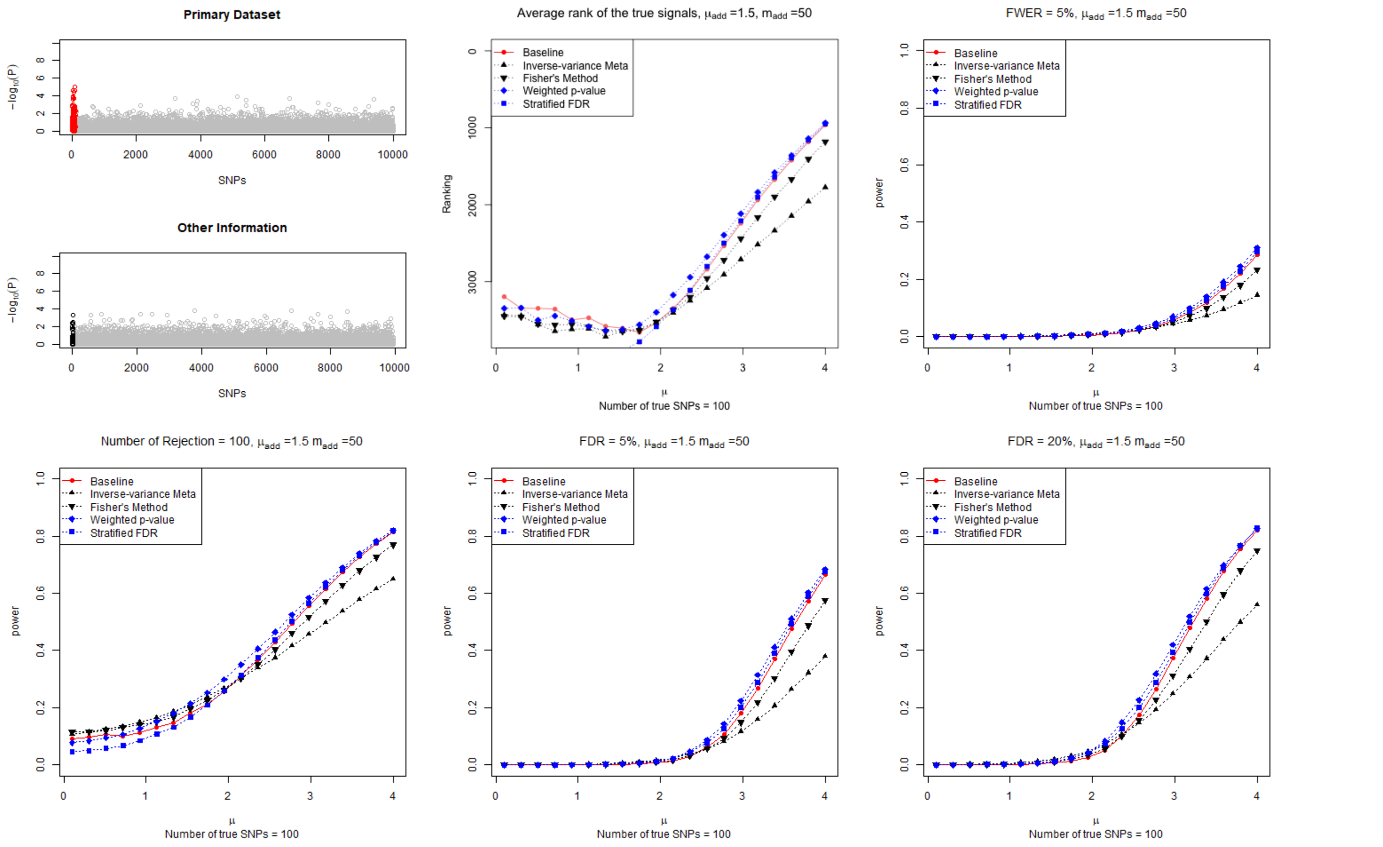

Figure S14. Results of simulation study design III for scenario (4) in Figure S7 from Category II type of informativeness, with the true effect size μ_1 for the m_1 causal SNPs varying from 0.1 to 4. Partially Informative: $m_{add} = 50$, $\mu_{add} = 1.5$, and all m_{add} SNPs coincide with (some of) the m_1 SNPs. We assumed the total number of SNPs $m = 10,000$, among which the first $m_1 = 100$ SNPs (in red on top-left panel) are truly associated. The corresponding summary statistics z_i's were drawn, independently, from $N(\mu_1, 1)$ for the m_1 associated SNPs, and from $N(0,1)$ for the remaining null SNPs. We then assumed $z_{i,add}$'s (in black on top-left panel) as the additional information available, which were drawn, independently, from $N(\mu_{add}, 1)$ for m_{add} SNPs and $N(0,1)$ for the remaining SNPs.

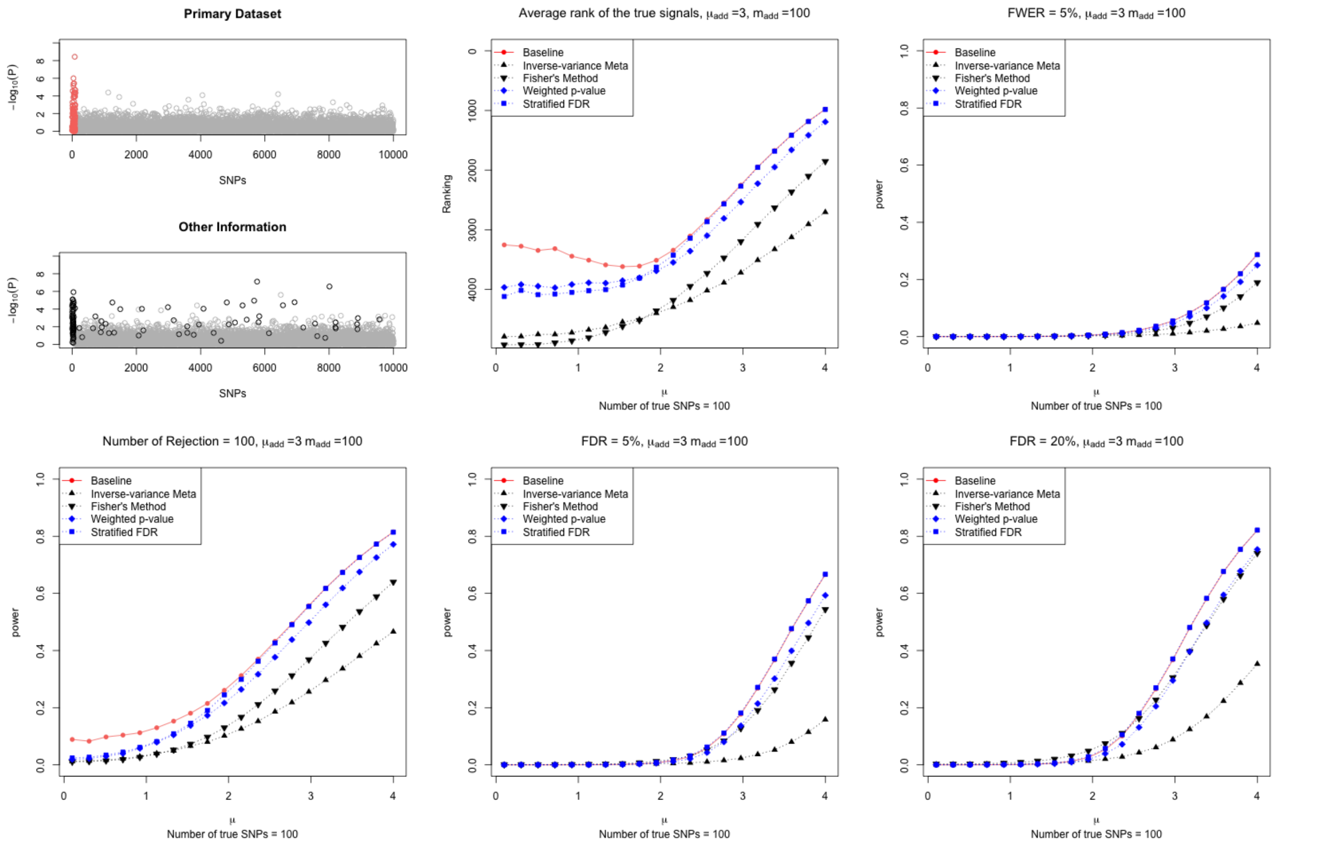

Figure S15. **Results of simulation study design III for scenario (5) in Figure S7 from Category III type of informativeness, with the true effect size μ_1 for the m_1 causal SNPs varying from 0.1 to 4.** Partially Misleading: $m_{add} = 100$, $\mu_{add} = 3$ but only 50 out of the m_{add} SNPs coincide with 50 of the m_1 SNPs. We assumed the total number of SNPs $m = 10,000$, among which the first $m_1 = 100$ SNPs (in red on top-left panel) are truly associated. The corresponding summary statistics z_i's were drawn, independently, from $N(\mu_1, 1)$ for the m_1 associated SNPs, and from $N(0, 1)$ for the remaining null SNPs. We then assumed $z_{i,add}$'s (in black on top-left panel) as the additional information available, which were drawn, independently, from $N(\mu_{add}, 1)$ for m_{add} SNPs and $N(0, 1)$ for the remaining SNPs.

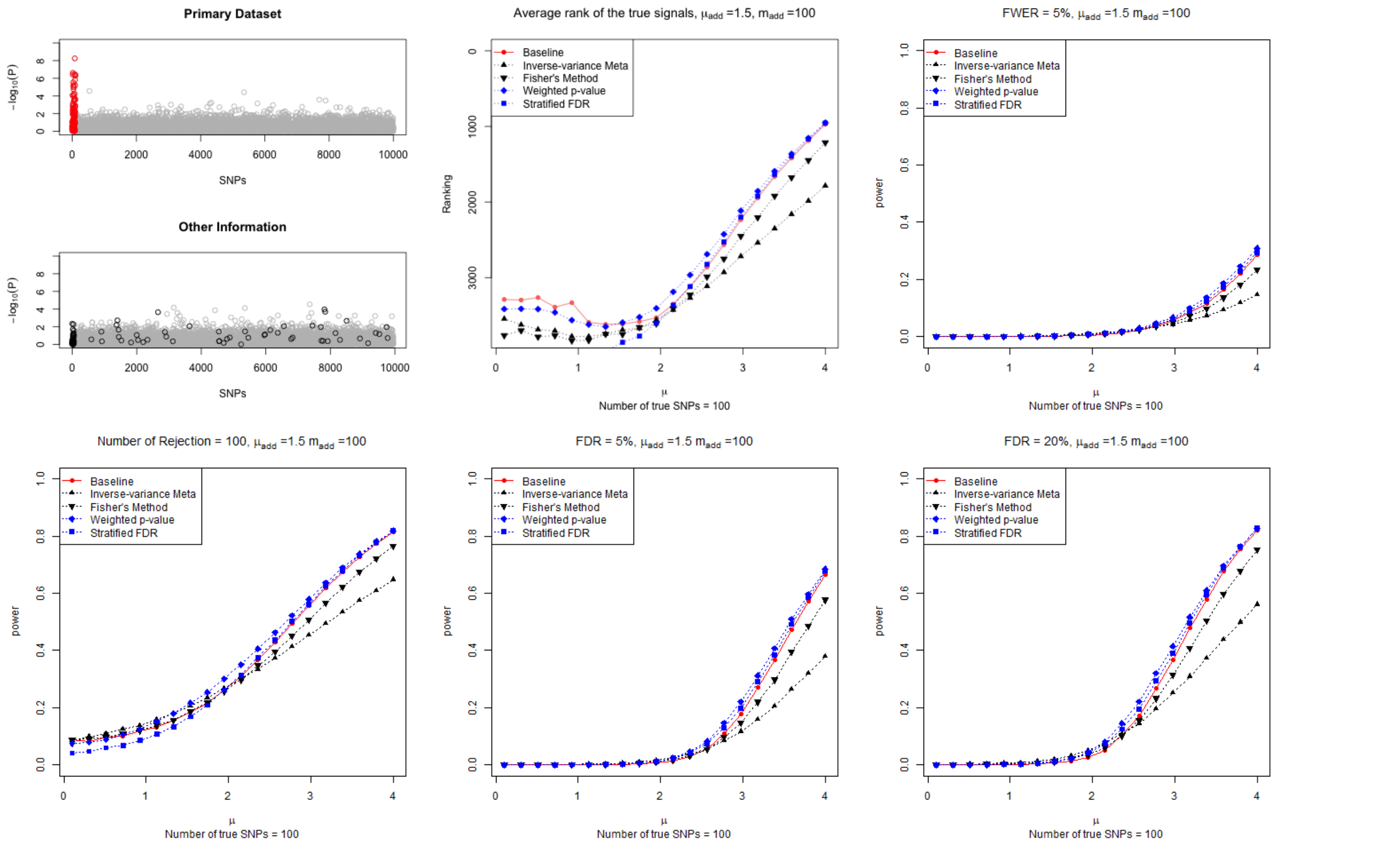

Figure S16. **Results of simulation study design III for scenario (6) in Figure S7 from Category III type of informativeness, with the true effect size μ_1 for the m_1 causal SNPs varying from 0.1 to 4.** Partially Misleading: $m_{add} = 100$, $\mu_{add} = 1.5$ but only 50 out of the m_{add} SNPs coincide with 50 of the m_1 SNPs. We assumed the total number of SNPs $m = 10,000$, among which the first $m_1 = 100$ SNPs (in red on top-left panel) are truly associated. The corresponding summary statistics z_i's were drawn, independently, from $N(\mu_1, 1)$ for the m_1 associated SNPs, and from $N(0,1)$ for the remaining null SNPs. We then assumed $z_{i,add}$'s (in black on top-left panel) as the additional information available, which were drawn, independently, from $N(\mu_{add}, 1)$ for m_{add} SNPs and $N(0,1)$ for the remaining SNPs.

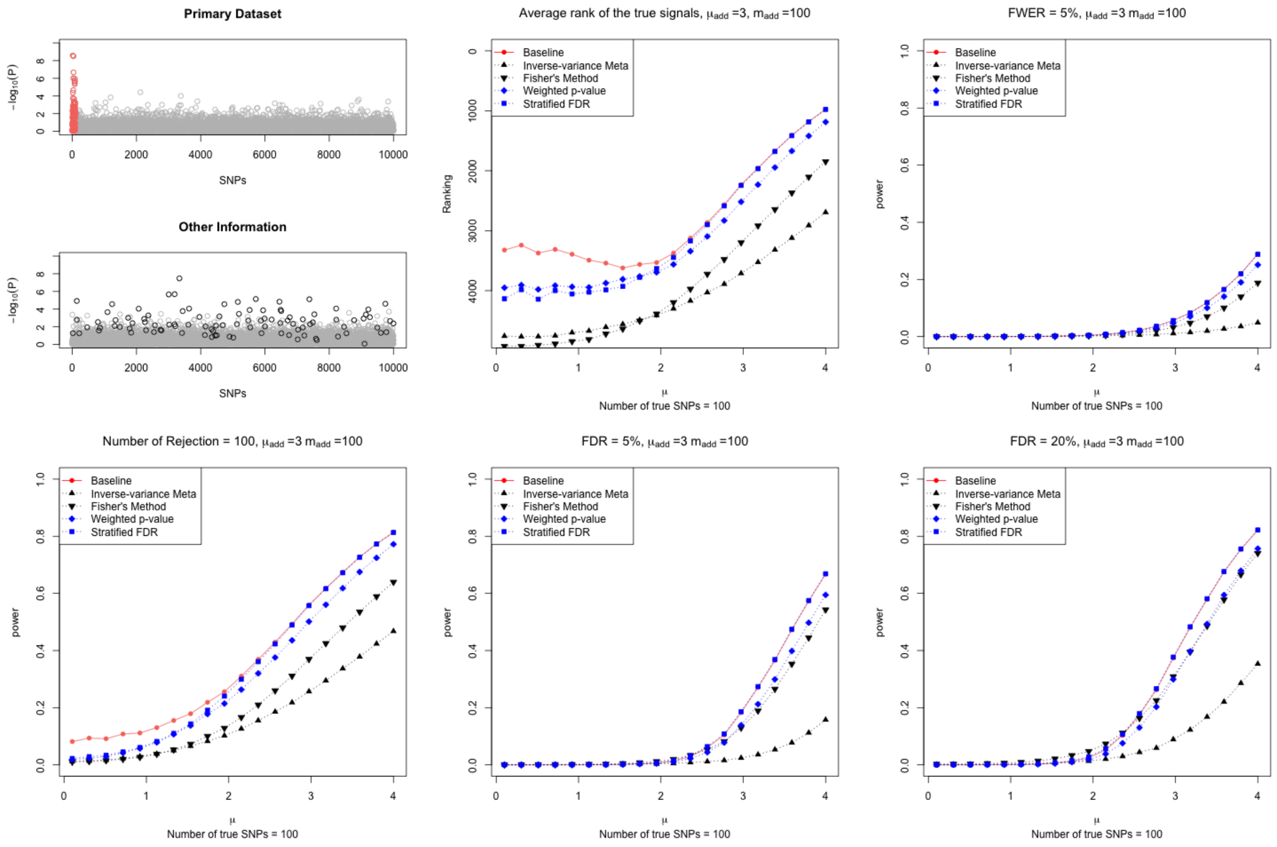

Figure S17. Results of simulation study design III for scenario (7) in Figure S7 from Category III type of informativeness, with the true effect size μ_1 for the m_1 causal SNPs varying from 0.1 to 4. Completely Misleading: $m_{add} = 100$ and $\mu_{add} = 3$, but none of the m_{add} SNPs coincide with the m_1 SNPs. We assumed the total number of SNPs $m = 10,000$, among which the first $m_1 = 100$ SNPs (in red on top-left panel) are truly associated. The corresponding summary statistics z_i's were drawn, independently, from $N(\mu_1, 1)$ for the m_1 associated SNPs, and from $N(0,1)$ for the remaining null SNPs. We then assumed $z_{i,add}$'s (in black on top-left panel) as the additional information available, which were drawn, independently, from $N(\mu_{add}, 1)$ for m_{add} SNPs and $N(0,1)$ for the remaining SNPs.

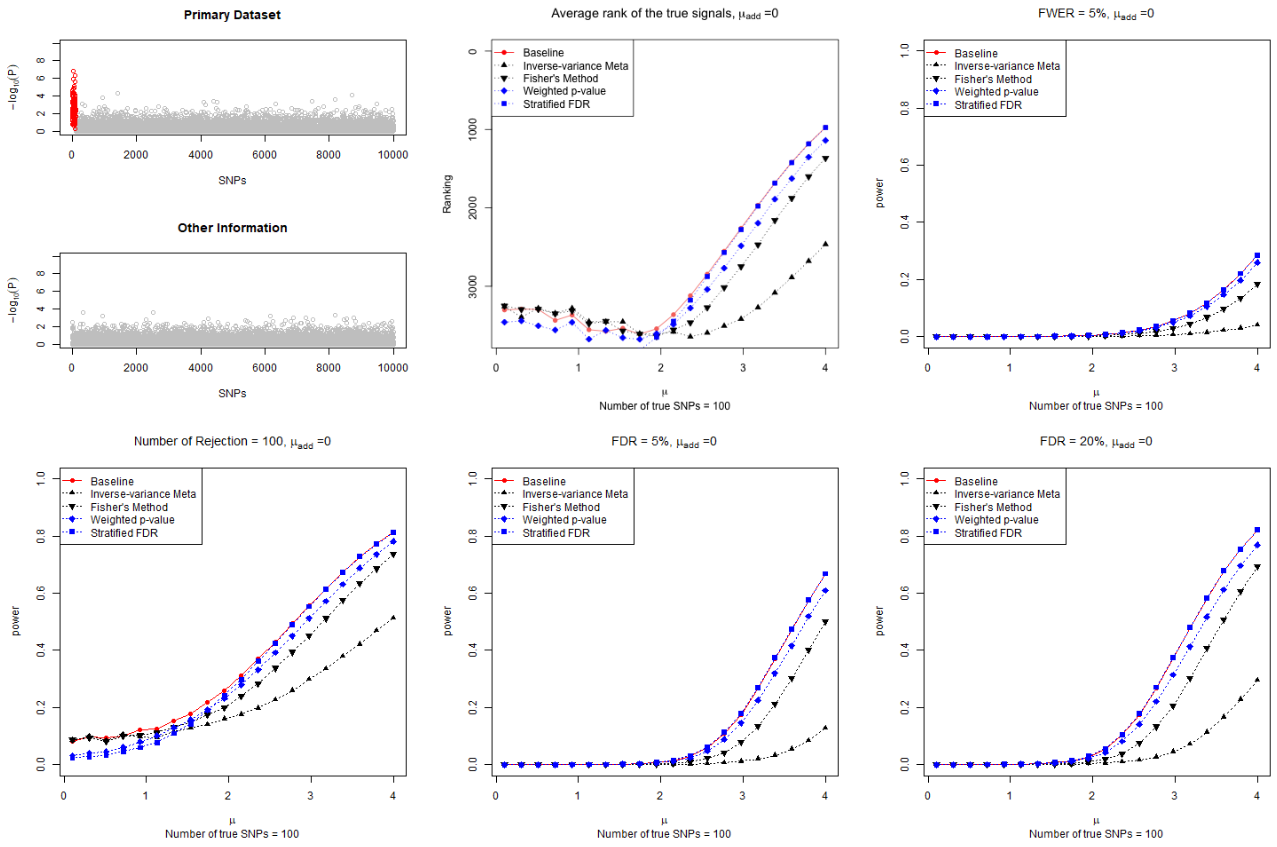

Figure S18. Results of simulation study design III for scenario (8) in Figure S7 from Category I type of informativeness, with the true effect size μ_1 for the m_1 causal SNPs varying from 0.1 to 4. Uninformative: $m_{add} = 0$ and $\mu_{add} = 0$. That is, the additional information available is white noise. We assumed the total number of SNPs $m = 10,000$, among which the first $m_1 = 100$ SNPs (in red on top-left panel) are truly associated. The corresponding summary statistics z_i's were drawn, independently, from $N(\mu_1, 1)$ for the m_1 associated SNPs, and from $N(0,1)$ for the remaining null SNPs. We then assumed $z_{i,add}$'s (in black on top-left panel) as the additional information available, which were drawn, independently, from $N(\mu_{add}, 1)$ for m_{add} SNPs and $N(0,1)$ for the remaining SNPs.

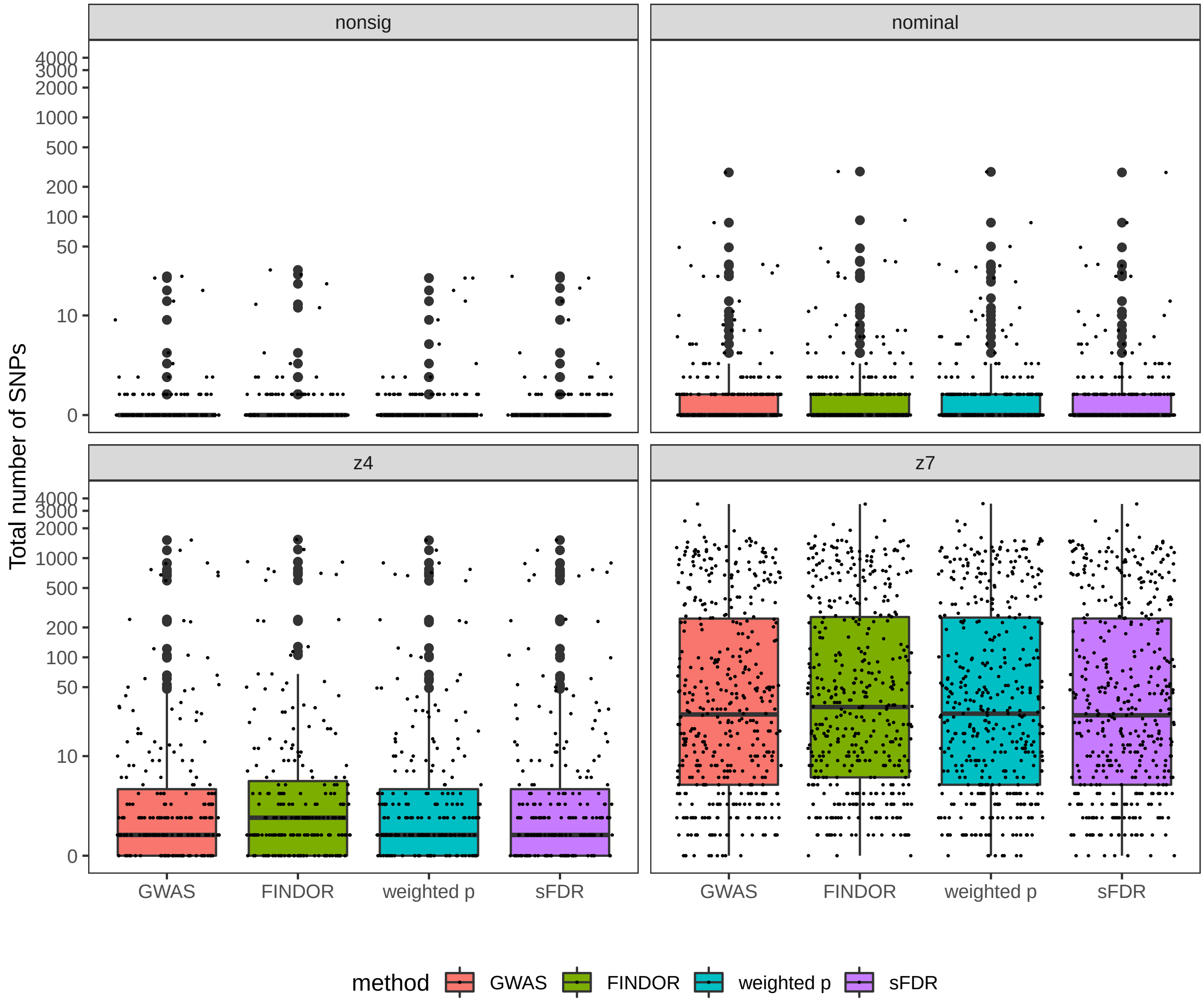

Figure S19. Results of using Eigen meta-scores for the total numbers of genome-wide significant independent loci of the UK Biobank GWAS application study, before and after data-integration with functional annotations, stratified by the four phenotype categories. In each figure, the total number of significant loci identified based on the UK Biobank GWAS data alone serves as a baseline. The GWAS baseline box-plot is followed by the box-plots for the total numbers of significant loci after integrating the UK Biobank GWAS summary statistics with functional annotations using FINDOR (using 75 individual annotation scores), and the weighted p-value and stratified FDR control methods (each using the Eigen meta-score), analyzing 7,895,174 variants for each of the 1,132 UK Biobank traits. The 1,132 traits were rated by Nealelab as having medium to high confidence for their heritability estimates, and they fall into four categories: nonsig (182 traits; heritability testing p-value $p > 0.05$), nominal (277 traits; $p < 0.05$), z4 (235 traits; $p < 3.17 \times 10^{-5}$), and z7 (438 traits; $p < 1.28 \times 10^{-12}$). Independent loci were defined using PLINK's LDclumping algorithm with a 1 Mb window and an r^2 threshold of 0.1.

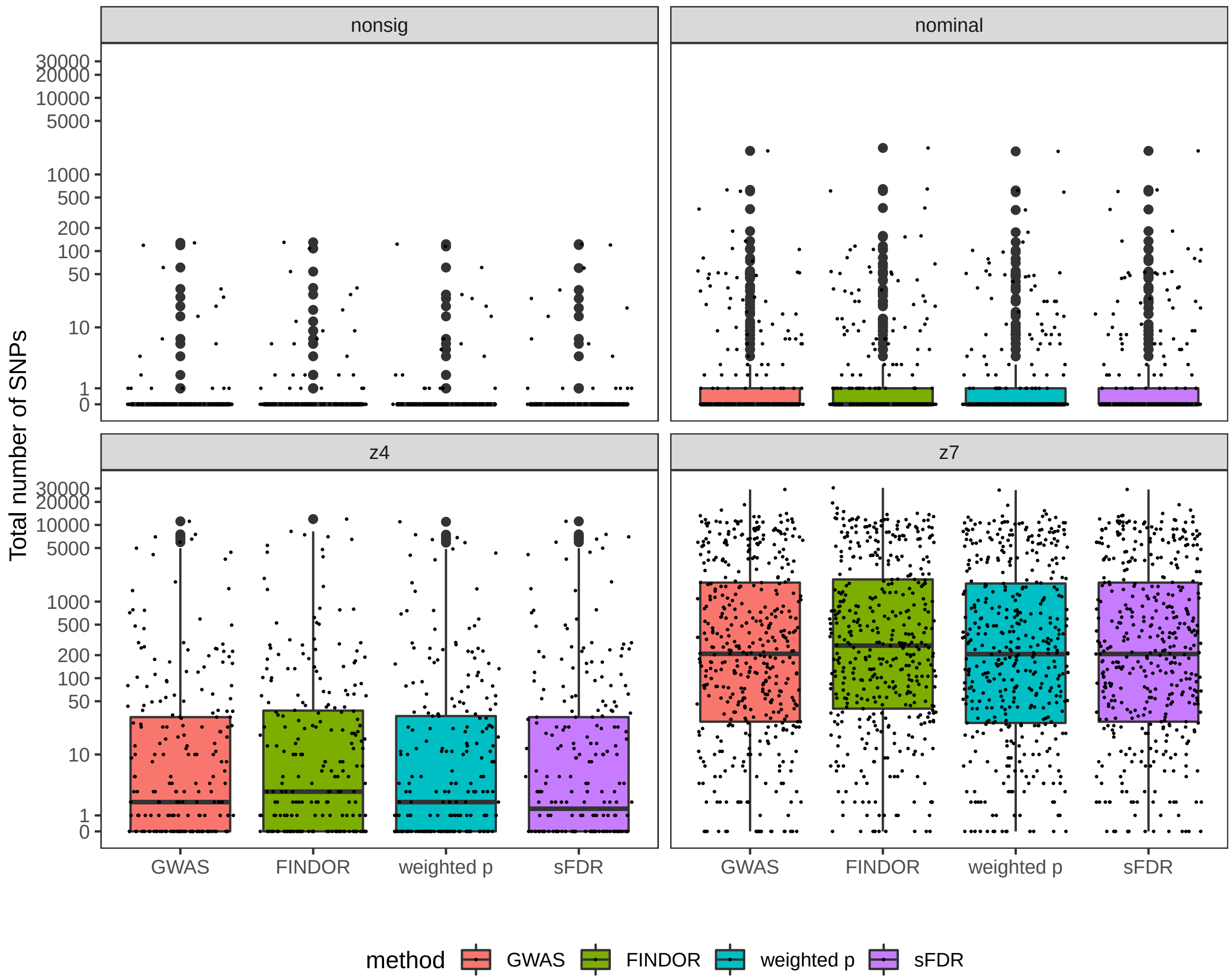

Figure S20. Results of counting significant SNPs instead of loci for the total numbers of genome-wide significant SNPs of the UK Biobank GWAS application study, before and after data-integration with functional annotations, stratified by the four phenotype categories. In each figure, the total number of significant loci identified based on the UK Biobank GWAS data alone serves as a baseline. The GWAS baseline box-plot is followed by the box-plots for the total numbers of significant loci after integrating the UK Biobank GWAS summary statistics with functional annotations using FINDOR (using 75 individual annotation scores), and the weighted p-value and stratified FDR control methods (each using the CADD meta-score), analyzing 7,895,174 variants for each of the 1,132 UK Biobank traits. The 1,132 traits were rated by Nealelab as having medium to high confidence for their heritability estimates, and they fall into four categories: nonsig (182 traits; heritability testing p-value $p > 0.05$), nominal (277 traits; $p < 0.05$), z4 (235 traits; $p < 3.17 \times 10^{-5}$), and z7 (438 traits; $p < 1.28 \times 10^{-12}$). Independent loci were defined using PLINK's LDclumping algorithm with a 1 Mb window and an r^2 threshold of 0.1.

(a) nonsig

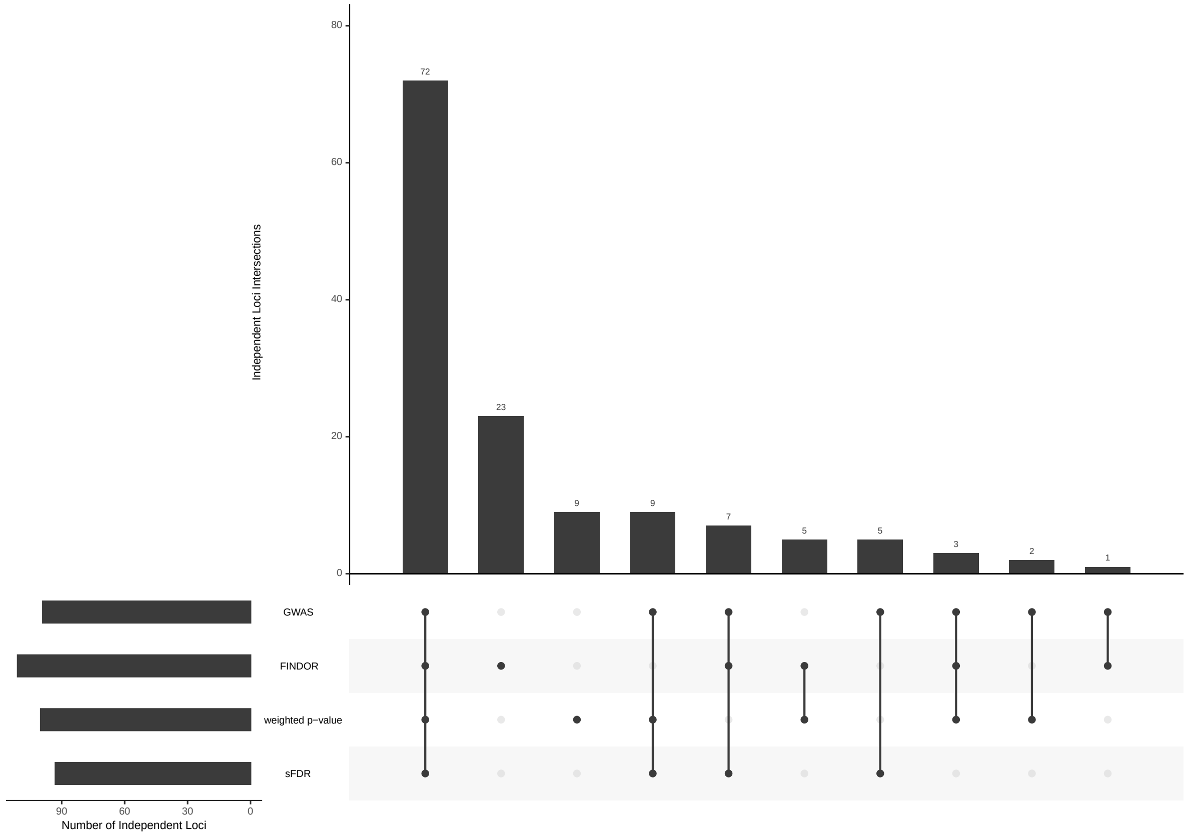

(b) nominal

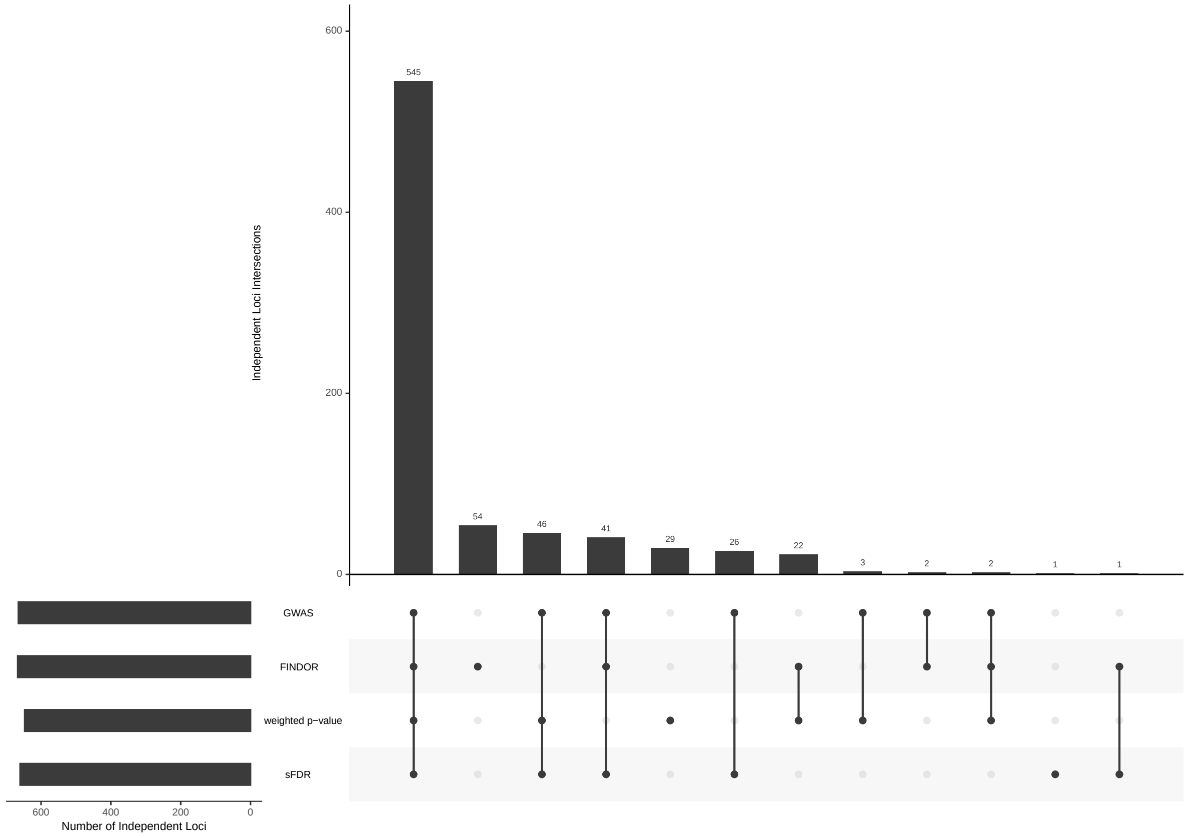

(c) z4

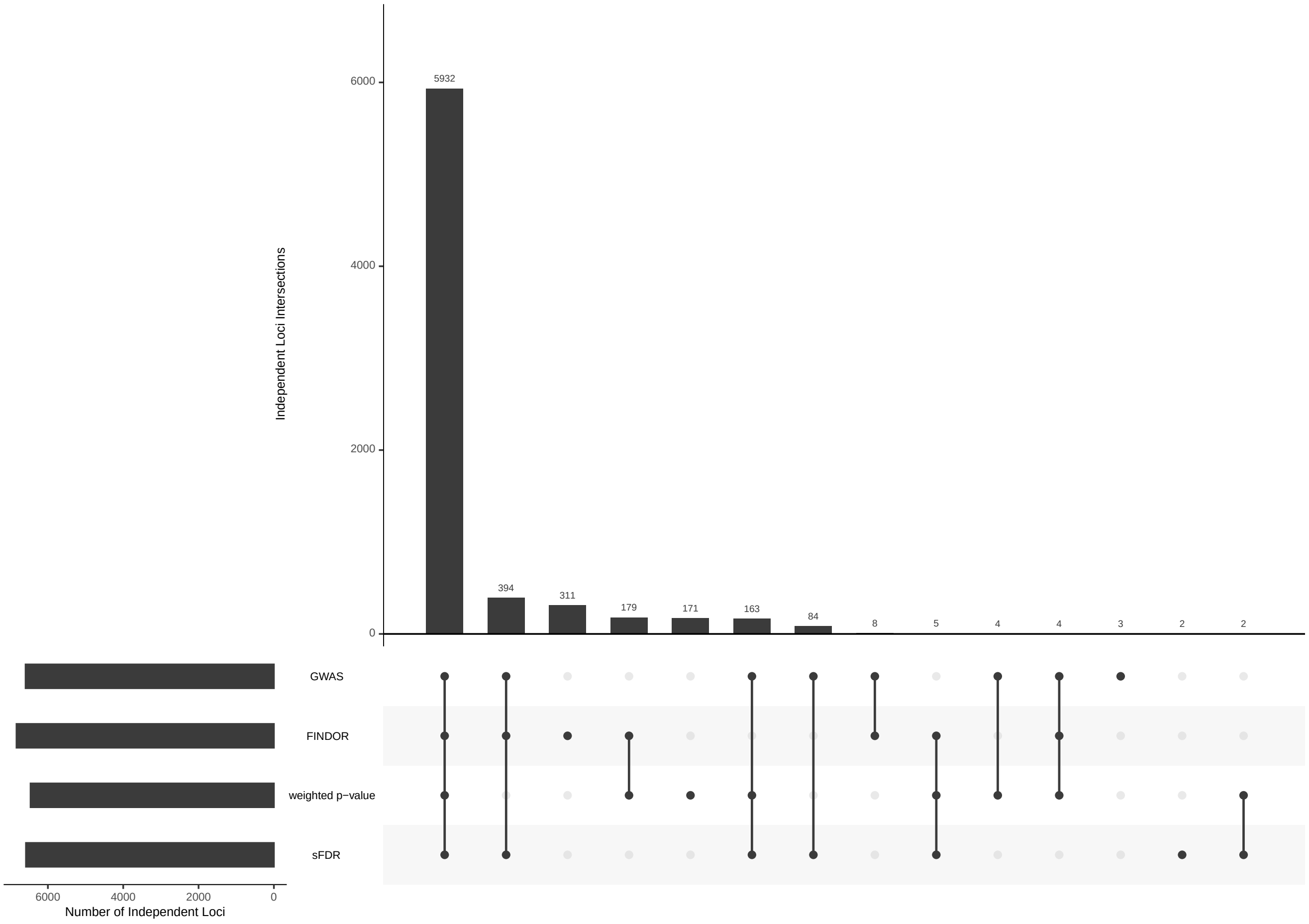

Independent Loci Intersections
6000
4000
2000
0
5932
394
311
179
171
163
84
8
5
4
4
3
2
2
GWAS
FINDOR
weighted p−value
sFDR
6000
4000
2000
0
Number of Independent Loci

(d) z7

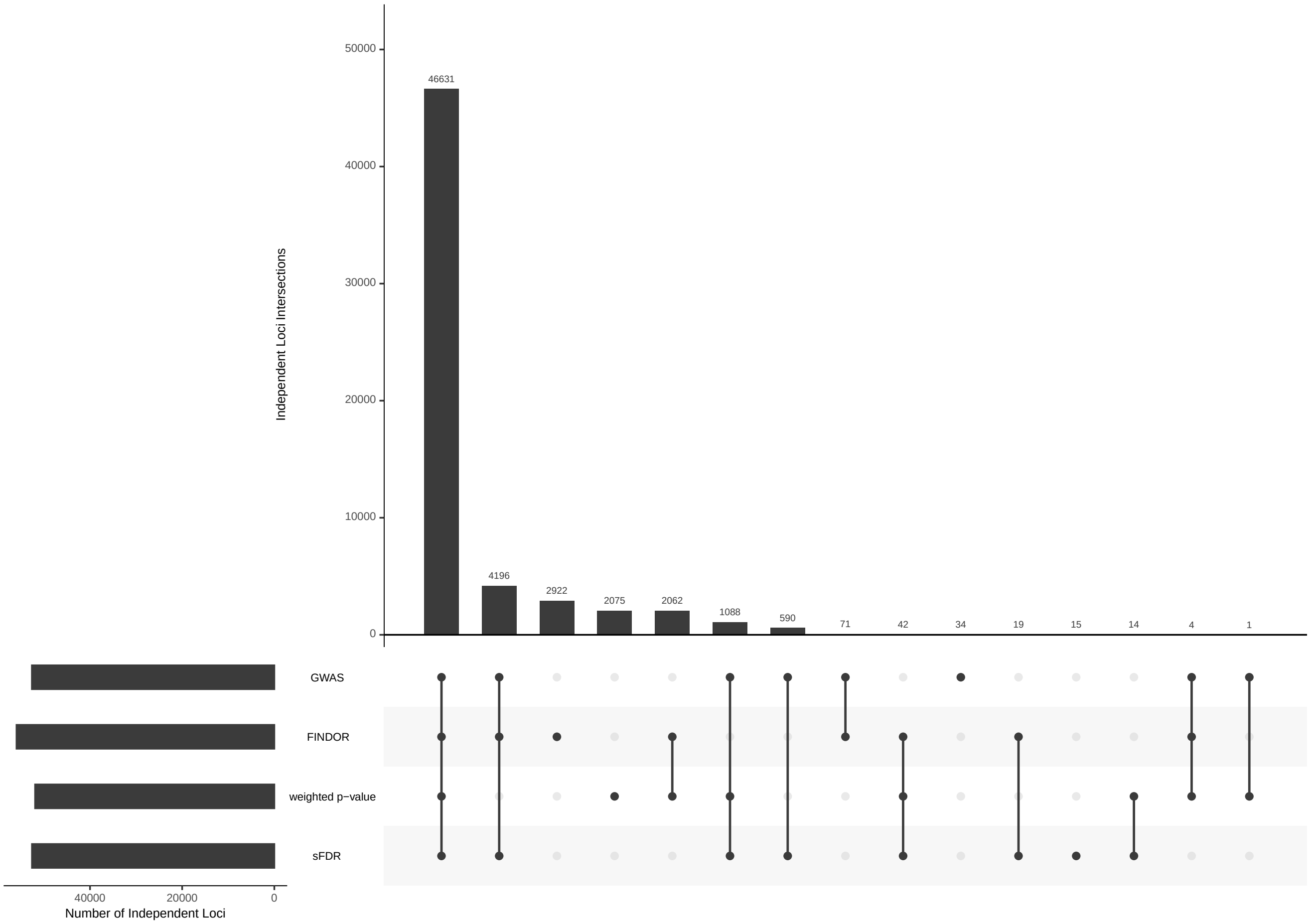

Figure S21. Intersection of independent, significant loci of the UK Biobank GWAS application study, before and after data-integration with functional annotations, stratified by the four phenotype categories. The three data-integration methods integrated the UK Biobank GWAS summary statistics with functional annotations using FINDOR (using 75 individual annotation scores), and the weighted p-value andstratified FDR control methods (each using the CADD meta-score), analyzing 7,895,174 variants for each of the 1,132 UK Biobank traits. The 1,132 traits were rated by Nealelab having medium to high confidence for their heritability estimates, and they fall into four categories: nonsig (182 traits; heritability testing $p > 0.05$), nominal (277 traits; $p < 0.05$), z4 (235 traits; $p < 3.17 \times 10^{-5}$), and z7 (438 traits; $p < 1.28 \times 10^{-12}$). Independent loci were defined using PLINK's LDclumping algorithm with a 1 Mb window and an r^2 threshold of 0.1.

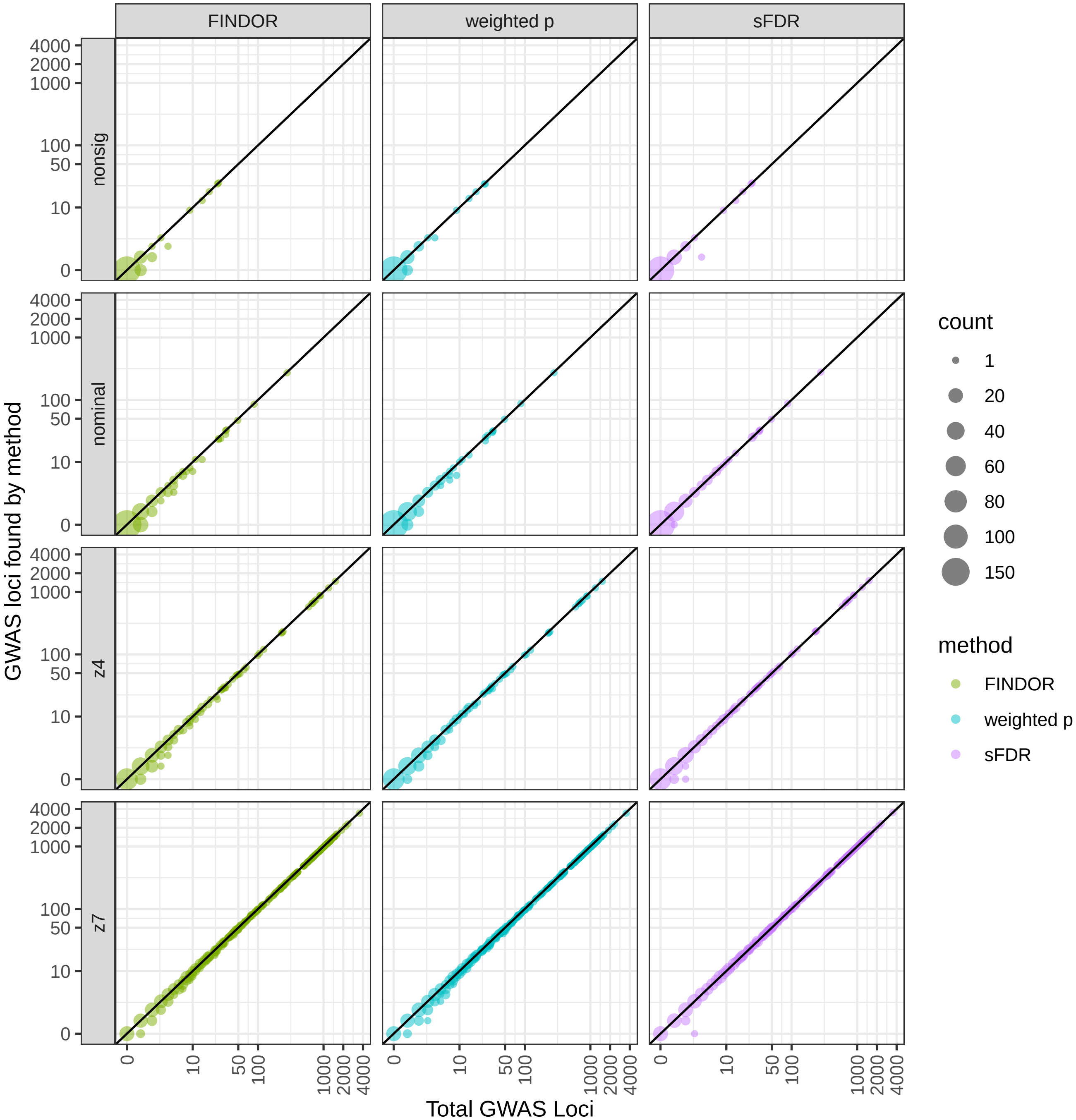

Figure S22. Contrast of the numbers of significant loci before and after data-integration for all 1,132 traits, i.e. $m_{1,t} \geq 0$. $m_{1,t}$ is the number of genome-wide significant independent loci prior to data-integration for trait t, The three data-integration methods integrated the UK Biobank GWAS summary statistics with functional annotations using FINDOR (using 75 individual annotation scores), and the weighted p-value and stratified FDR control methods (each using the CADD meta-score), analyzing 7,895,174 variants for each of the 1,132 UK Biobank traits. The 1,132 traits were rated by Nealelab having medium to high confidence for their heritability estimates, and they fall into four categories: nonsig (182 traits; heritability testing $p > 0.05$), nominal (277 traits; $p < 0.05$), z4 (235 traits; $p < 3.17 \times 10^{-5}$), and z7 (438 traits; $p < 1.28 \times 10^{-12}$). Independent loci were defined using PLINK's LDclumping algorithm with a 1 Mb window and an r^2 threshold of 0.1.

(a) Stratified by the four trait types

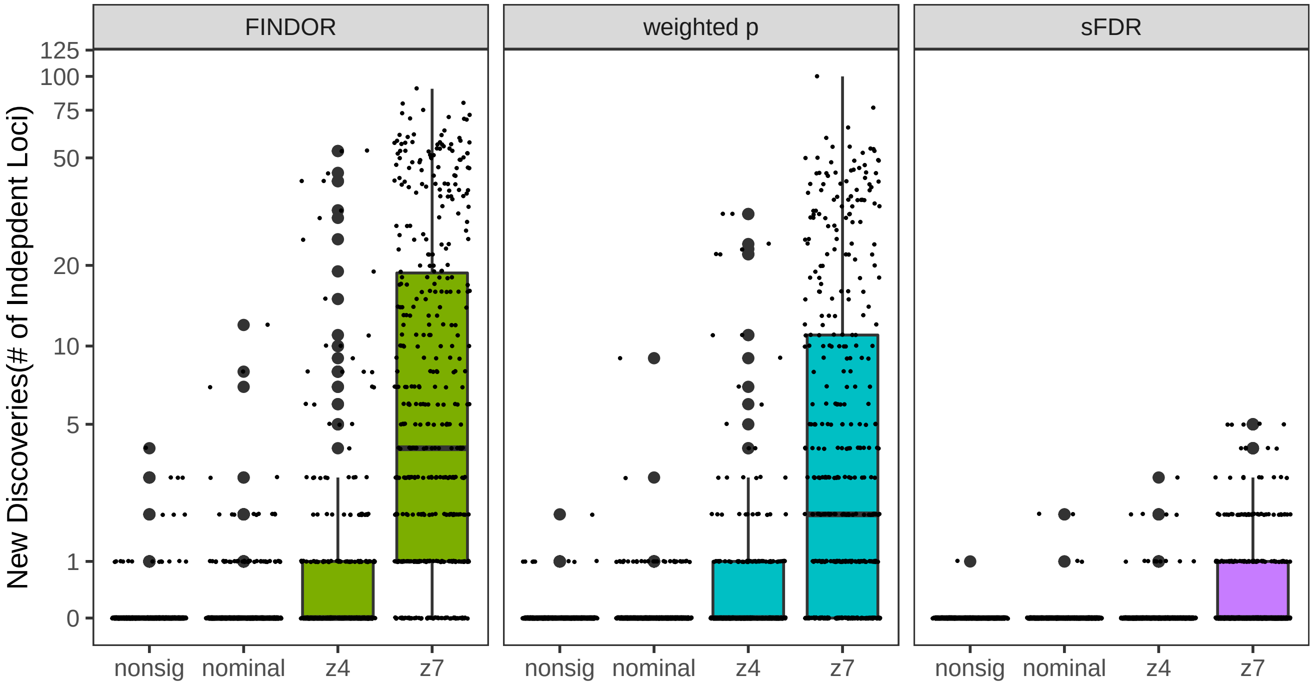

(b) Contrasting with the SNP-heritability estimates

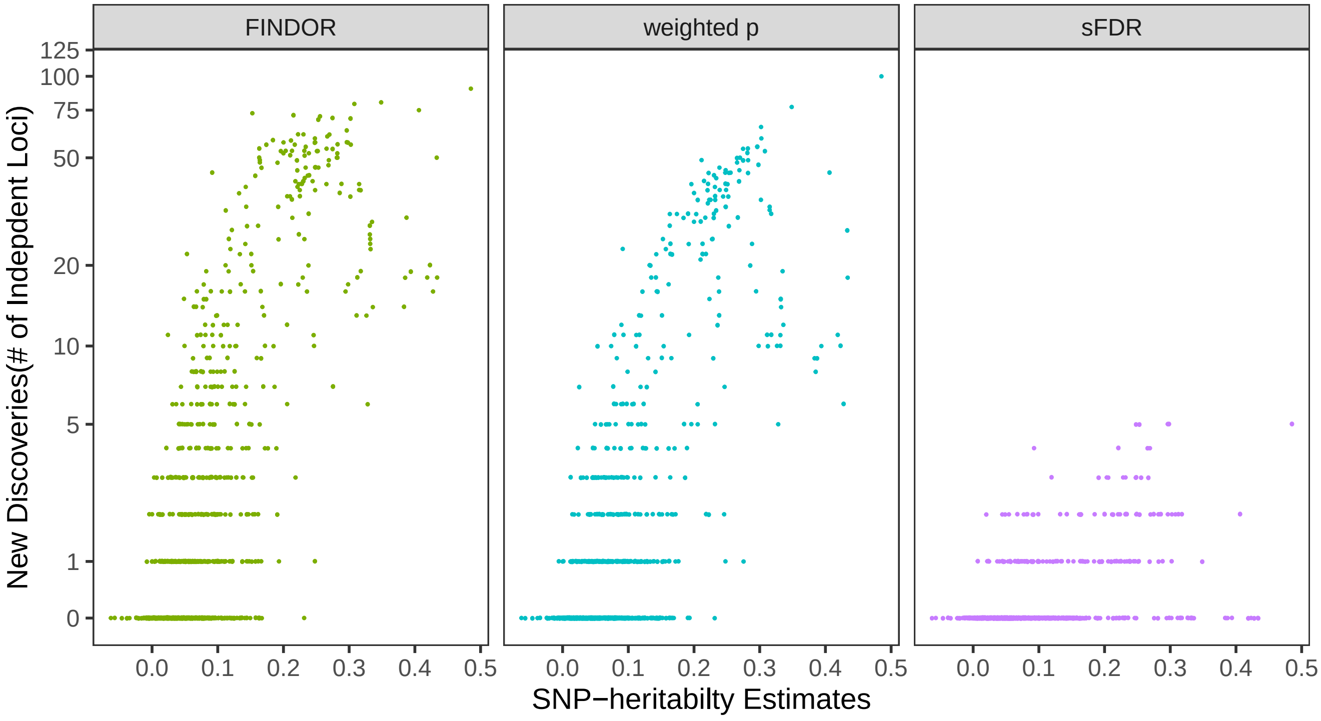

Figure S23. The Eigen results of the total *New Discoveries* of the application study (A) stratified by the four trait types or (B) Contrasting with the SNP-heritability estimates. The three data-integration methods integrated the UK Biobank GWAS summary statistics with functional annotations using FINDOR (using 75 individual annotation scores), and the weighted p-value andstratified FDR control methods (each using the Eigen meta-score), analyzing 7,895,174 variants for each of the 1,132 UK Biobank traits. The 1,132 traits were rated by Nealelab having medium to high confidence for their heritability estimates, and they fall into four categories: nonsig (182 traits; heritability testing $p > 0.05$), nominal (277 traits; $p < 0.05$), z4 (235 traits; $p < 3.17 \times 10^{-5}$), and z7 (438 traits; $p < 1.28 \times 10^{-12}$). Independent loci were defined using PLINK's LDclumping algorithm with a 1 Mb window and an r^2 threshold of 0.1.

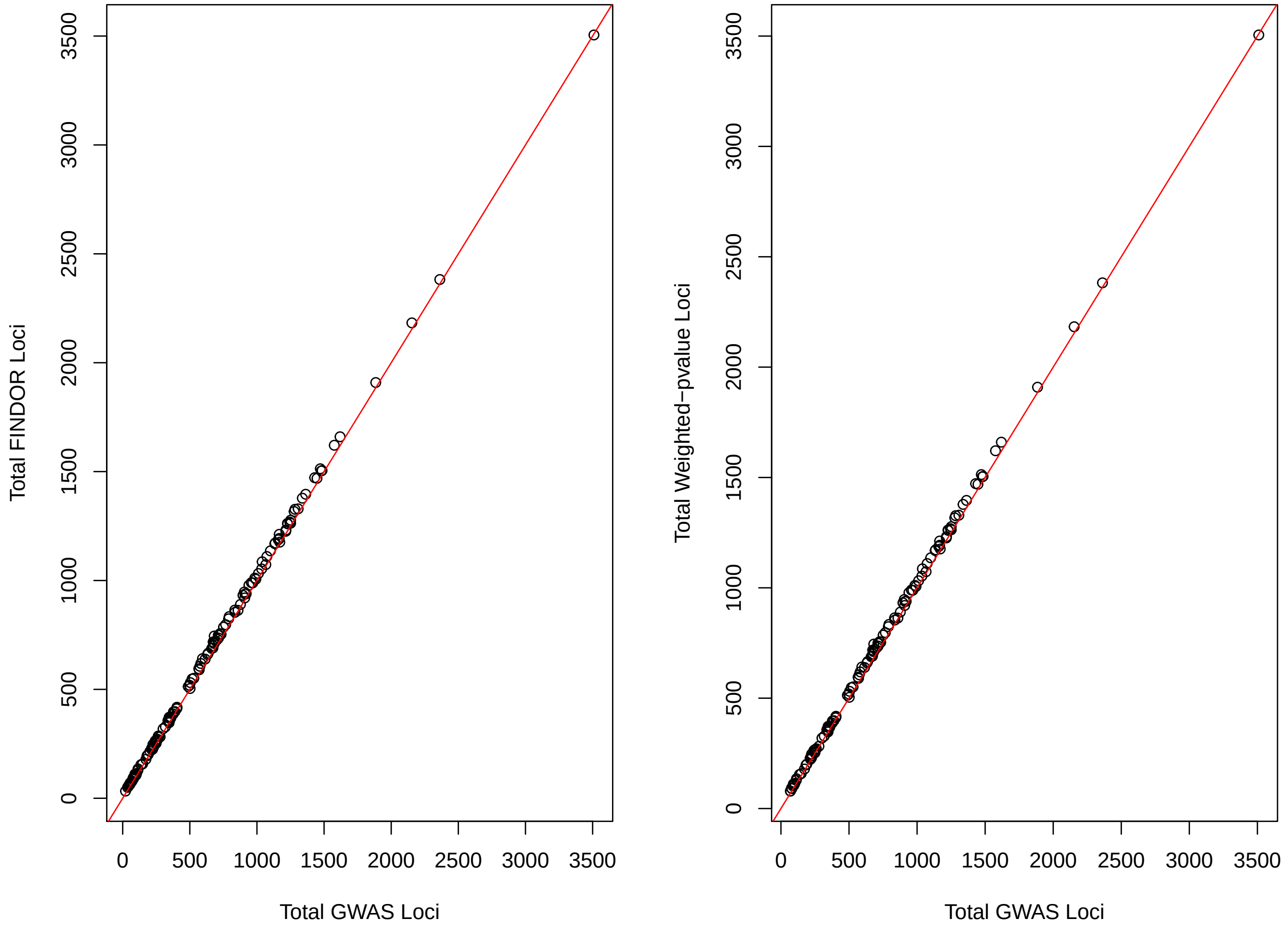

Figure S24. **Contrast of the numbers of significant loci before and after data-integration for traits in the z7 category with more than ten** *New Discoveries*: 153 traits by FINDOR and130 traits by weighted p-value; no traits by sFDR. As the two data-integration methods also had loss of significant loci that were present in the baseline GWAS, the total numbers of significant loci before and after data-integration are similar. The contrast plots for all 1,132 traits analyzed are shown in Figure S22

(a) Stratified by phenotype categories

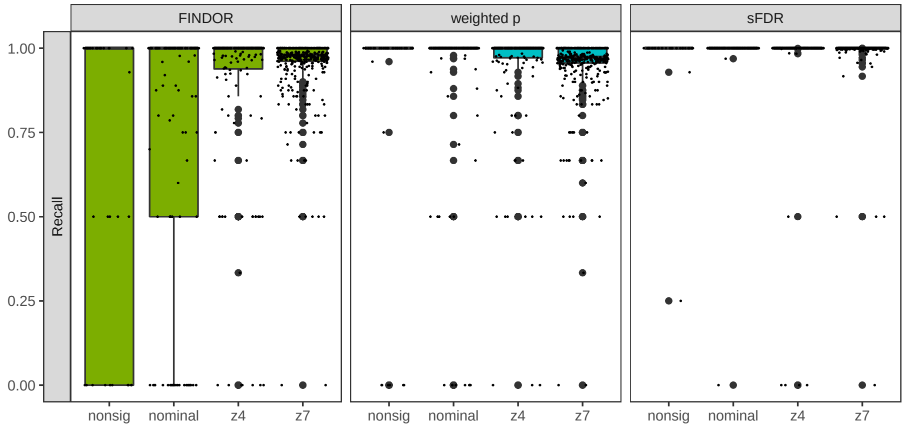

(b) Stratified by SNP-heritability estimates

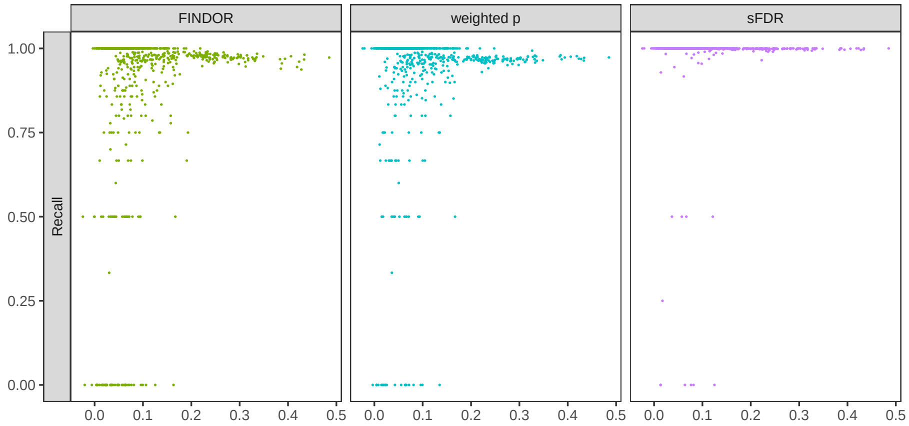

Figure S25. ***Recall*** **results of the UK Biobank GWAS application study stratified by (A) the four phenotype categories (B) SNP-heritability estimates, for all 722 traits with $m_{1,t>0}$.** $Recall_t = TP_t/m_{1,t}$, where $m_{1,t}$ is the number of genome-wide significant independent loci prior to data-integration for trait t, and TP_t is the number of true positives after data-integration. The three data-integration methods integrated the UK Biobank GWAS summary statistics with functional annotations using FINDOR (using 75 individual annotation scores), and the weighted p-value andstratified FDR control methods (each using the CADD meta-score), analyzing 7,895,174 variants for each of the 1,132 UK Biobank traits. The 1,132 traits were rated by Nealelab having medium to high confidence for their heritability estimates, and they fall into four categories: nonsig (182 traits; heritability testing $p > 0.05$), nominal (277 traits; $p < 0.05$), z4 (235 traits; $p < 3.17 \times 10^{-5}$), and z7 (438 traits; $p < 1.28 \times 10^{-12}$). Independent loci were defined using PLINK's LDclumping algorithm with a 1 Mb window and an r^2 threshold of 0.1.

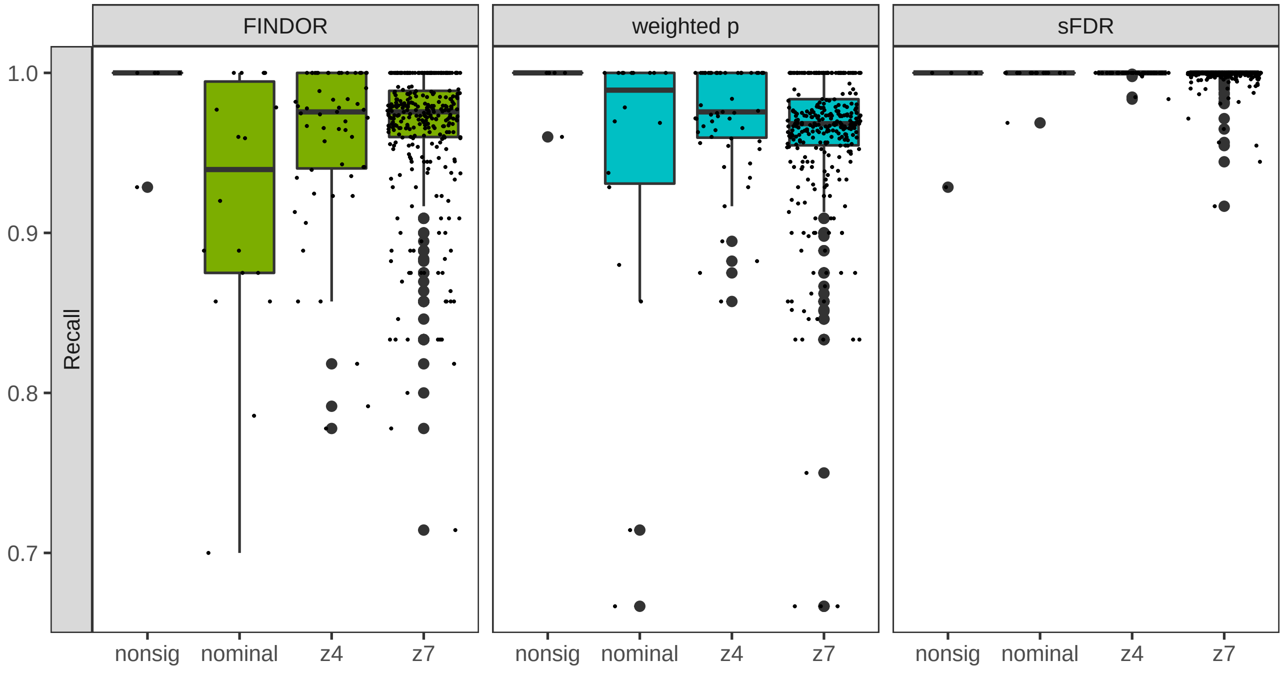

Figure S26. ***Recall* results of the UK Biobank GWAS application study stratified by the four phenotype categories, for 402 traits with $m_{1,t>5}$.** $Recall_t = TP_t/m_{1,t}$, where $m_{1,t}$ is the number of genome-wide significant independent loci prior to data-integration for trait t, and TP_t is the number of true positives after data-integration. The three data-integration methods integrated the UK Biobank GWAS summary statistics with functional annotations using FINDOR (using 75 individual annotation scores), and the weighted p-value andstratified FDR control methods (each using the CADD meta-score), analyzing 7,895,174 variants for each of the 1,132 UK Biobank traits. The 1,132 traits were rated by Nealelab having medium to high confidence for their heritability estimates, and they fall into four categories: nonsig (182 traits; heritability testing $p > 0.05$), nominal (277 traits; $p < 0.05$), z4 (235 traits; $p < 3.17 \times 10^{-5}$), and z7 (438 traits; $p < 1.28 \times 10^{-12}$). Independent loci were defined using PLINK's LDclumping algorithm with a 1 Mb window and an r^2 threshold of 0.1.

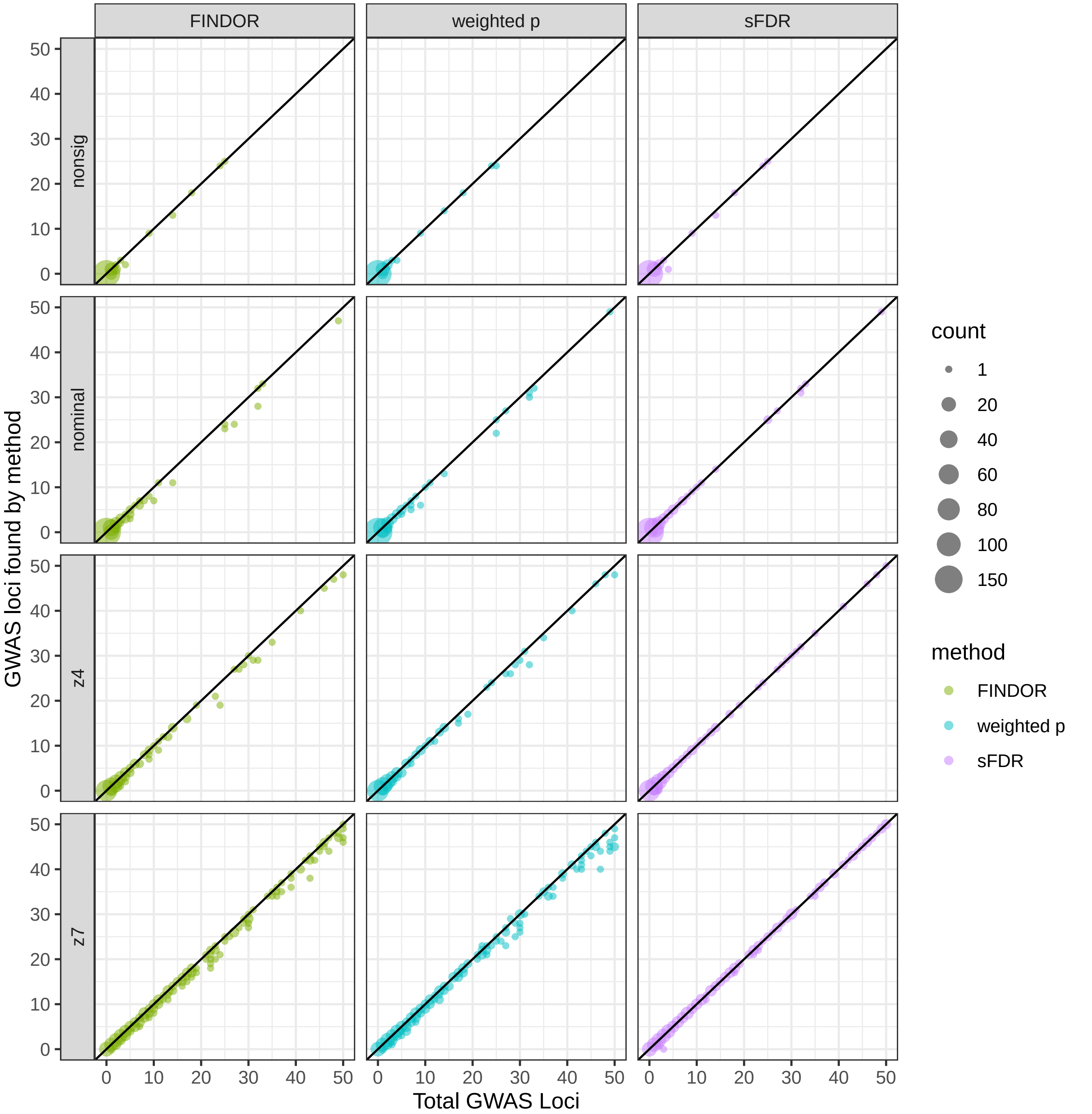

Figure S27. Contrast of the numbers of significant loci before and after data-integration for 942 traits with $m_{1,t} \leq 50$. $m_{1,t}$ is the number of genome-wide significant independent loci prior to data-integration for trait t, The three data-integration methods integrated the UK Biobank GWAS summary statistics with functional annotations using FINDOR (using 75 individual annotation scores), and the weighted p-value and stratified FDR control methods (each using the CADD meta-score), analyzing 7,895,174 variants for each of the 1,132 UK Biobank traits. The 1,132 traits were rated by Nealelab having medium to high confidence for their heritability estimates, and they fall into four categories: nonsig (182 traits; heritability testing $p > 0.05$), nominal (277 traits; $p < 0.05$), z4 (235 traits; $p < 3.17 \times 10^{-5}$), and z7 (438 traits; $p < 1.28 \times 10^{-12}$). Independent loci were defined using PLINK's LDclumping algorithm with a 1 Mb window and an r^2 threshold of 0.1.

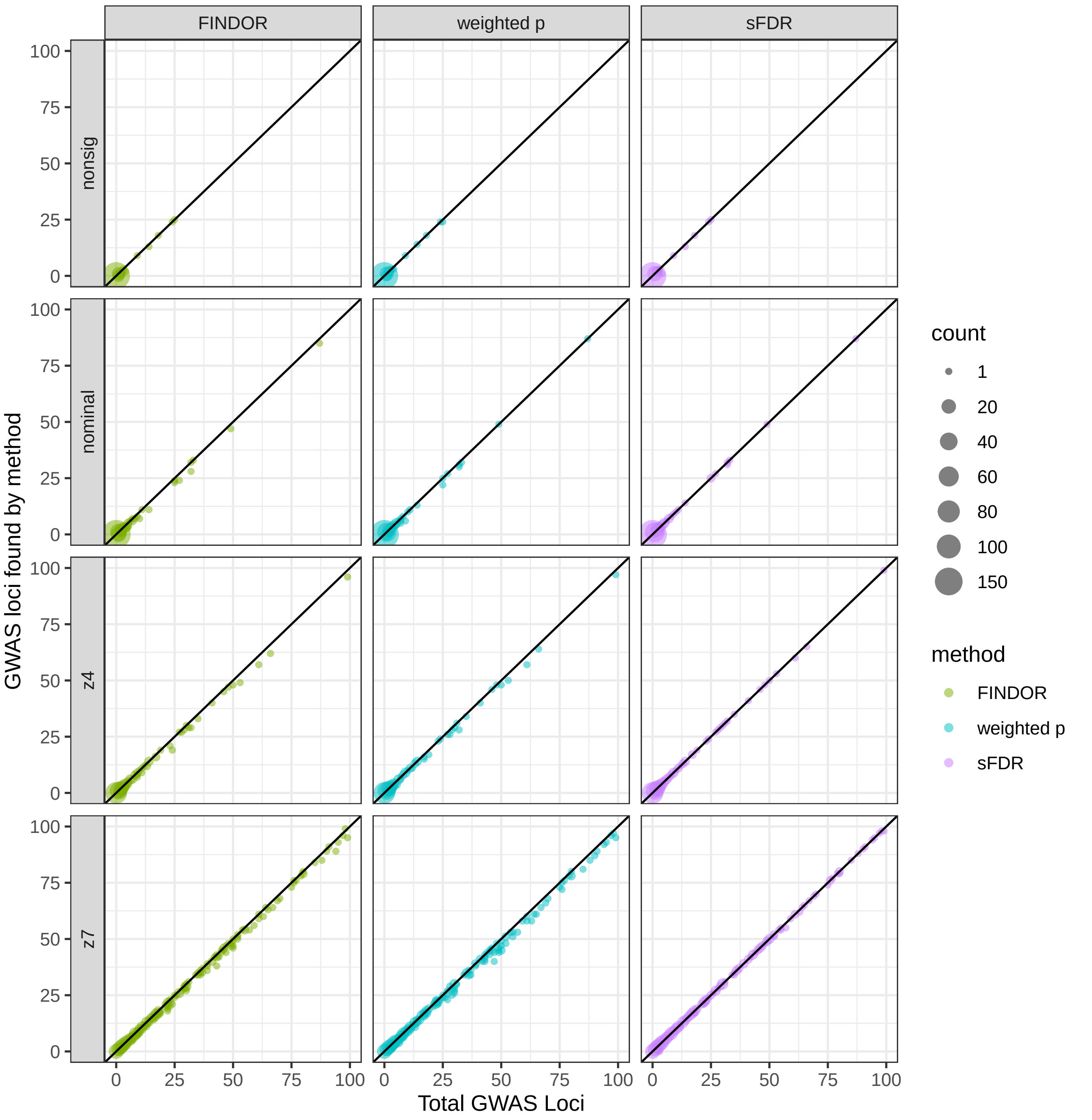

Figure S28. Contrast of the numbers of significant loci before and after data-integration for 980 traits with $m_{1,t} \leq 100$. $m_{1,t}$ is the number of genome-wide significant independent loci prior to data-integration for trait t, The three data-integration methods integrated the UK Biobank GWAS summary statistics with functional annotations using FINDOR (using 75 individual annotation scores), and the weighted p-value and stratified FDR control methods (each using the CADD meta-score), analyzing 7,895,174 variants for each of the 1,132 UK Biobank traits. The 1,132 traits were rated by Nealelab having medium to high confidence for their heritability estimates, and they fall into four categories: nonsig (182 traits; heritability testing $p > 0.05$), nominal (277 traits; $p < 0.05$), z4 (235 traits; $p < 3.17 \times 10^{-5}$), and z7 (438 traits; $p < 1.28 \times 10^{-12}$). Independent loci were defined using PLINK's LDclumping algorithm with a 1 Mb window and an r^2 threshold of 0.1.

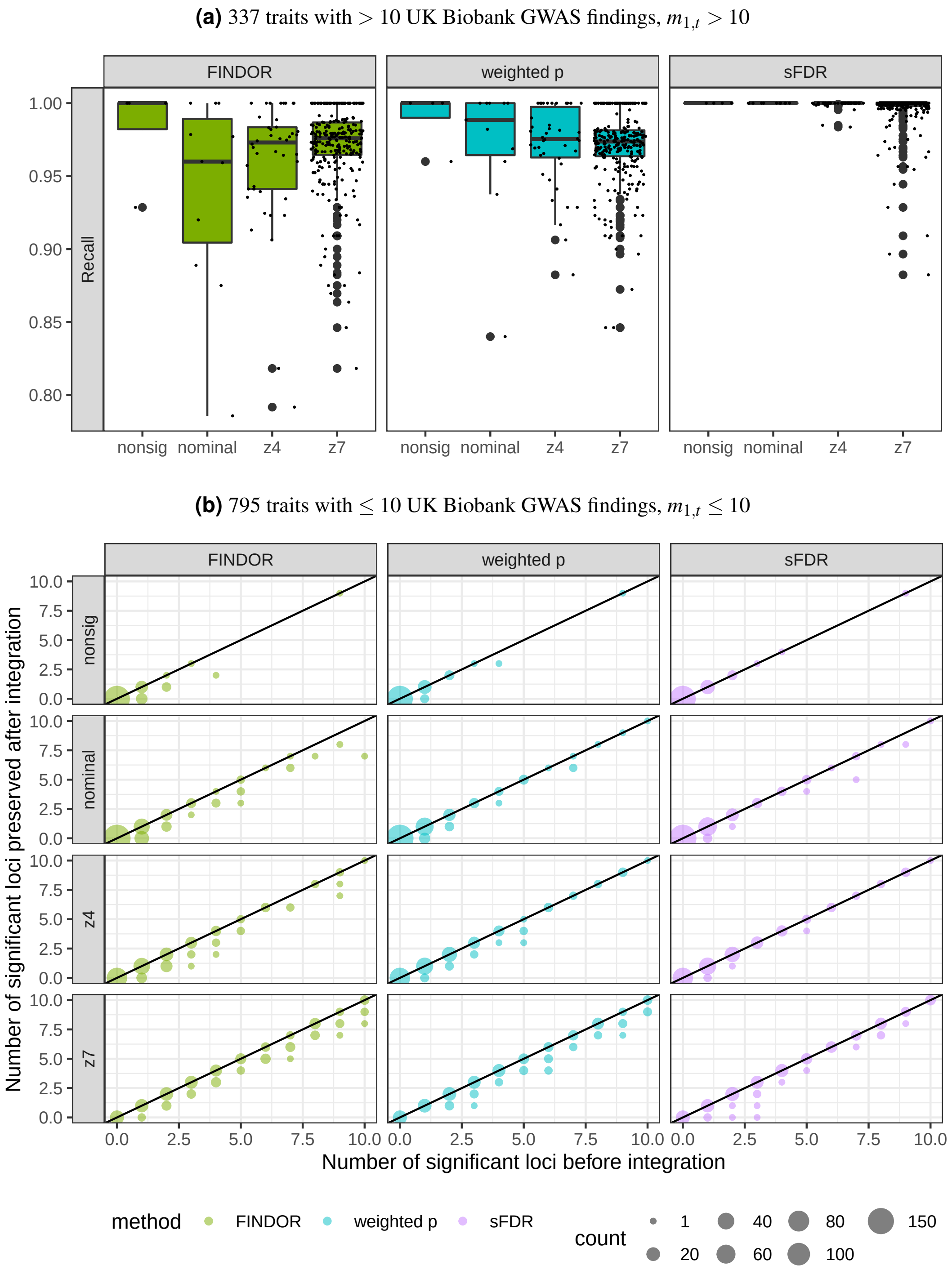

Figure S29. **Eigen results of the UK Biobank GWAS application study, before and after data-integration with functional annotations, stratified by the four phenotype categories.** (A) $Recall_t = TP_t/m_{1,t}$, where $m_{1,t}$ is the number of genome-wide significant independent loci prior to data-integration for trait t, and TP_t is the number of true positives after data-integration. *Recall* estimation is not stable when $m_{1,t}$ is small so for $m_{1,t} \leq 10$, (B) contrasts the number of significant loci preserved after data-integration with $m_{1,t}$. The three data-integration methods integrated the UK Biobank GWAS summary statistics with functional annotations using FINDOR (using 75 individual annotation scores), and the weighted p-value andstratified FDR control methods (each using the Eigen meta-score), analyzing 7,895,174 variants for each of the 1,132 UK Biobank traits. The 1,132 traits were rated by Nealelab having medium to high confidence for their heritability estimates, and they fall into four categories: nonsig (182 traits; heritability testing $p > 0.05$), nominal (277 traits; $p < 0.05$), z4 (235 traits; $p < 3.17 \times 10^{-5}$), and z7 (438 traits; $p < 1.28 \times 10^{-12}$). Independent loci were defined using PLINK's LDclumping algorithm with a 1 Mb window and an r^2 threshold of 0.1.

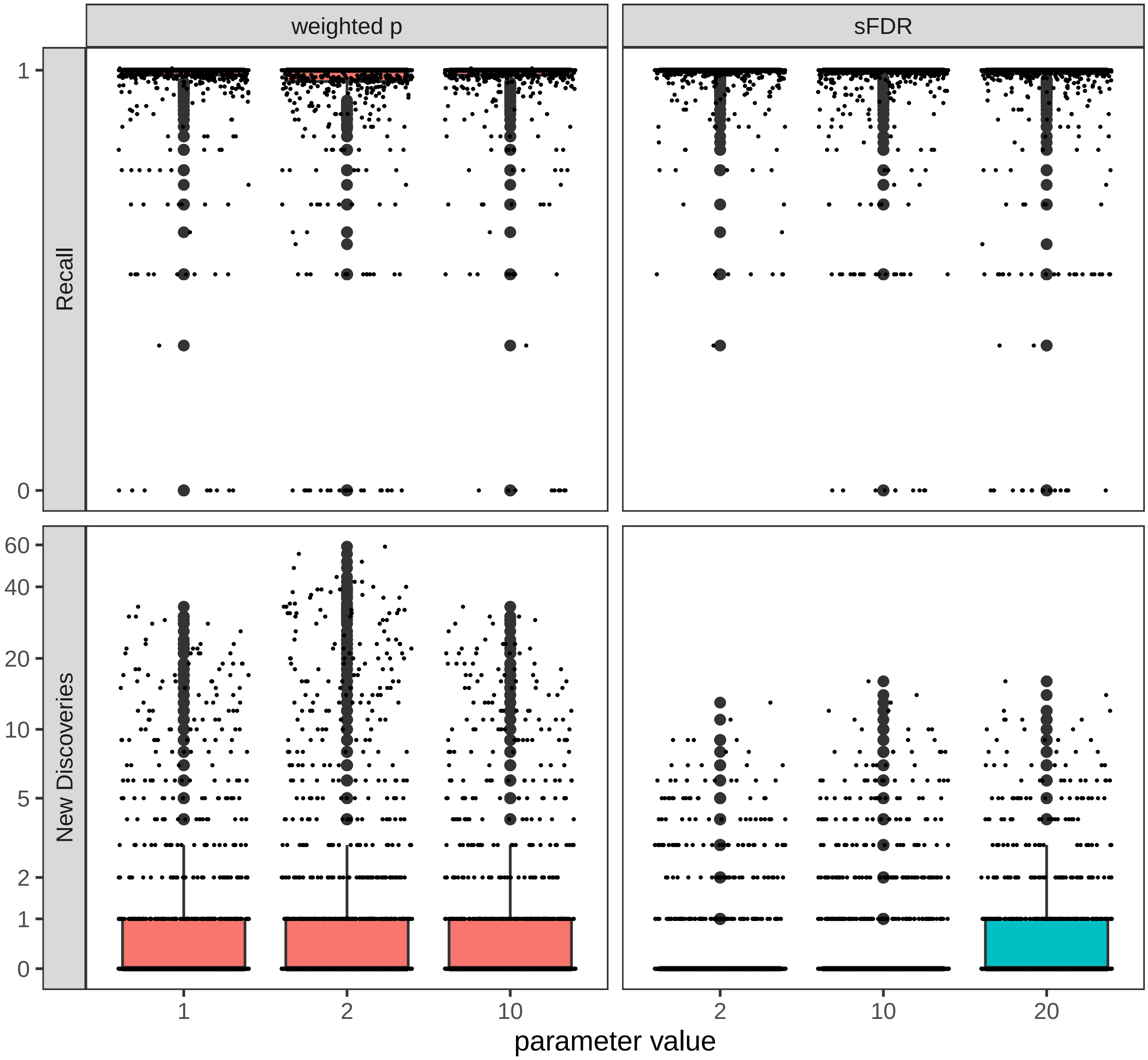

Figure S30. **Performance of weighted p-value and sFDR methods using different weighting schemes and stratification for the UK Biobank GWAS application study.** The two data-integration methods integrated the UKBiobank GWAS summary statistics with CADD meta-score, analyzing 7,895,174 variants for each of the 1,132 UK Biobank traits. Independent loci were defined using PLINK's LDclumping algorithm with a 1 Mb window and an r^2 threshold of 0.1. For the weighted p-value approach, the parameter values represent $\beta = 1$, 2 and 10 for the cumulative weighting scheme, $w_i = m\frac{\Phi(z_{i,add}-\beta)}{\sum_i \Phi(z_{i,add}-\beta)}$, where $z_{i,add}$ is the CADD meta-score for variant i. For the sFDR approach, the parameter values 2, 10 and 20 represent the number of strata used, where the stratification is based on quantiles of $z_{i,add}$, the CADD meta-score for variant i.

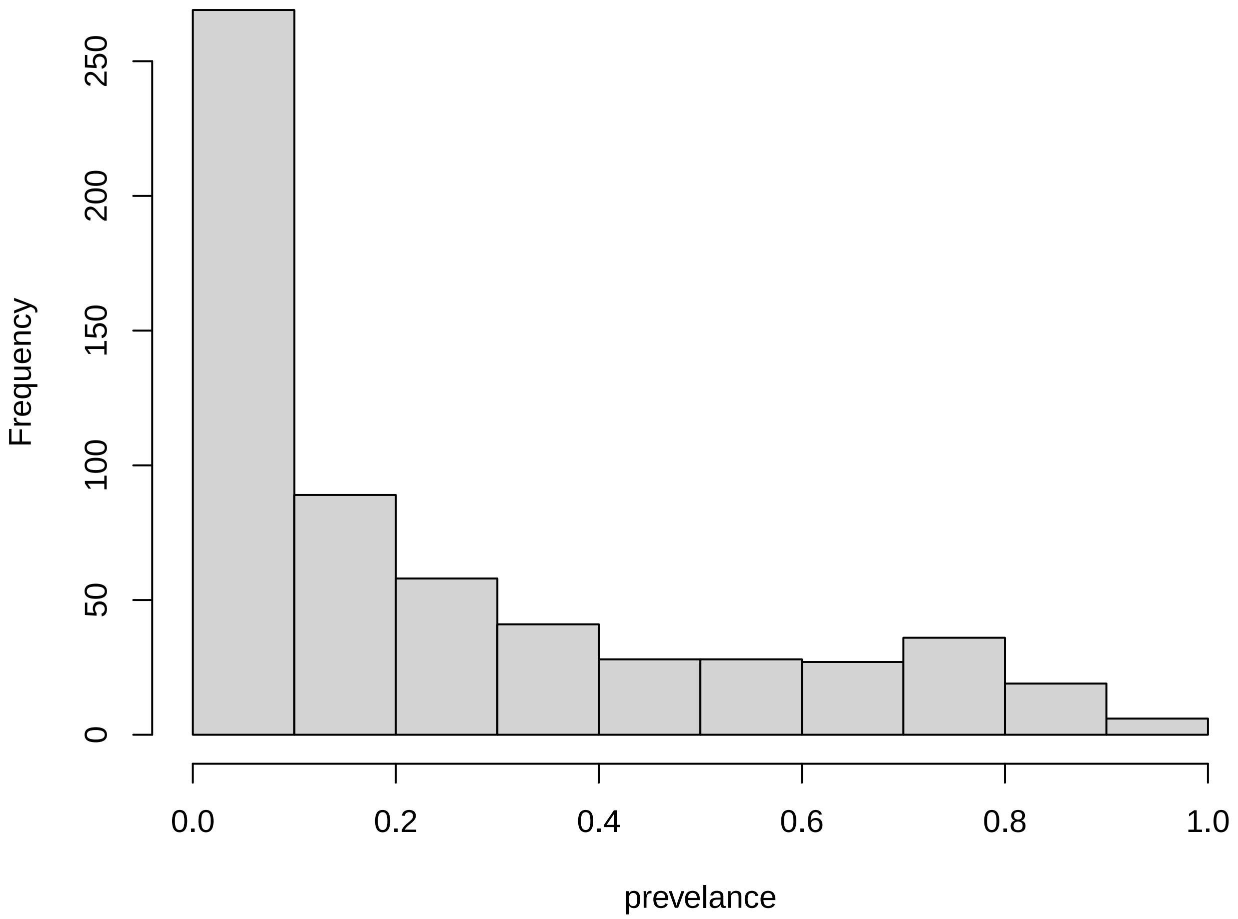

Figure S31. Histogram of prevalence for the 601 binary traits from the 1,132 UK Biobank traits analyzed in the application study.

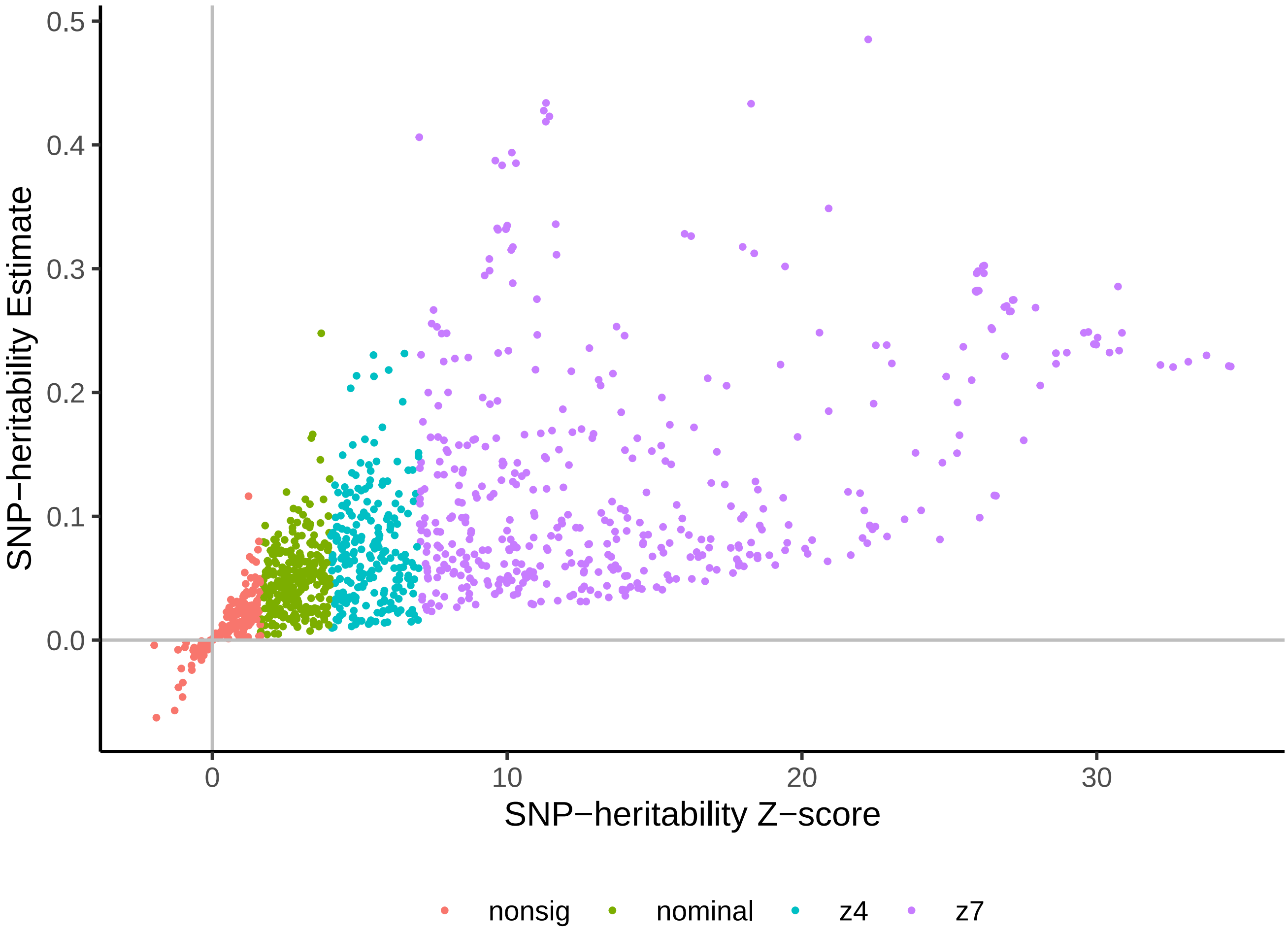

Figure S32. Contrast between SNP-heritability estimates and their z-scores for the 1,132 complex traits, based on the UK Biobank data analyzed by Nealelab. The 1,132 traits are categorized by Nealelab into four categories: nonsig (182 traits; $z < 1.96$), nominal (277 traits; $1.96 \leq z < 4$), z4 (235 traits; $4 \leq z < 7$), and z7 (438 traits; $z \geq 7$).

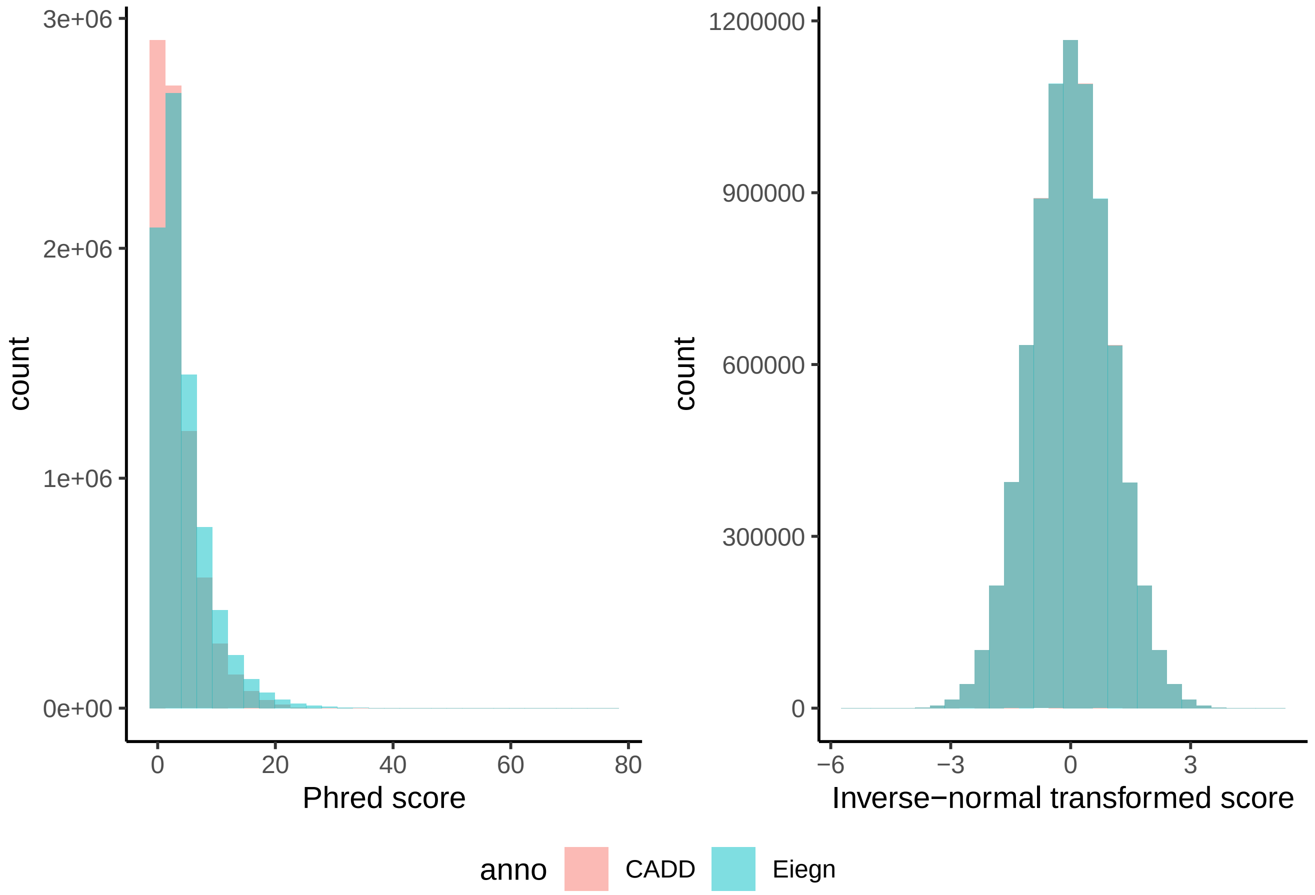

Figure S33. **Distributions of CADD and Eigen functional meta-scores.** The left figure shows the phred-scaled meta-scores, $-10\log_{10}(\text{ranks of the raw scores/total number SNPs})$, and right figure shows the inverse-normal transformed meta-scores.

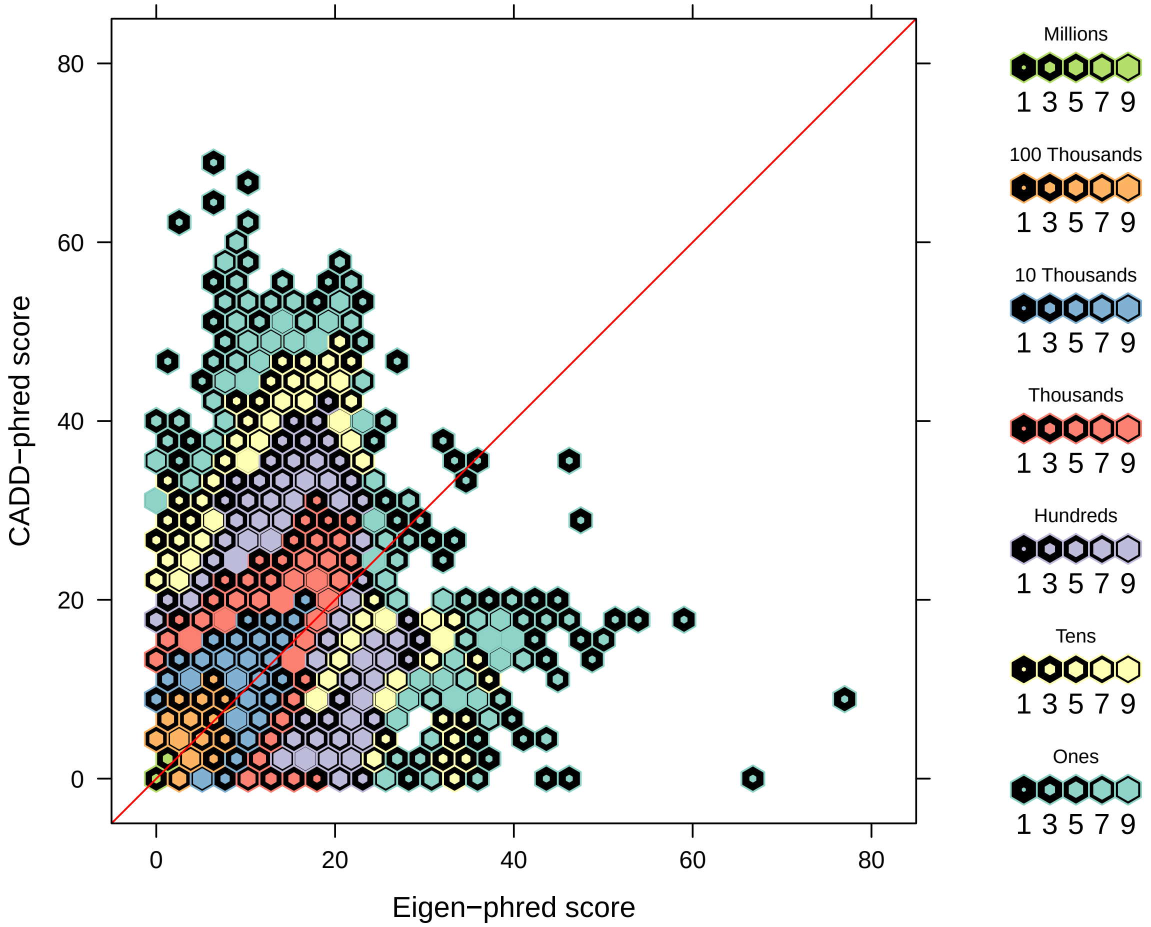

Figure S34. Contrast between CADD and Eigen functional meta-scores on the phred-scale. The phred scores are $-10\log_{10}(\text{ranks of the raw scores/total number SNPs})$. The color and inner size of each hexagon represents the number of SNP counts.

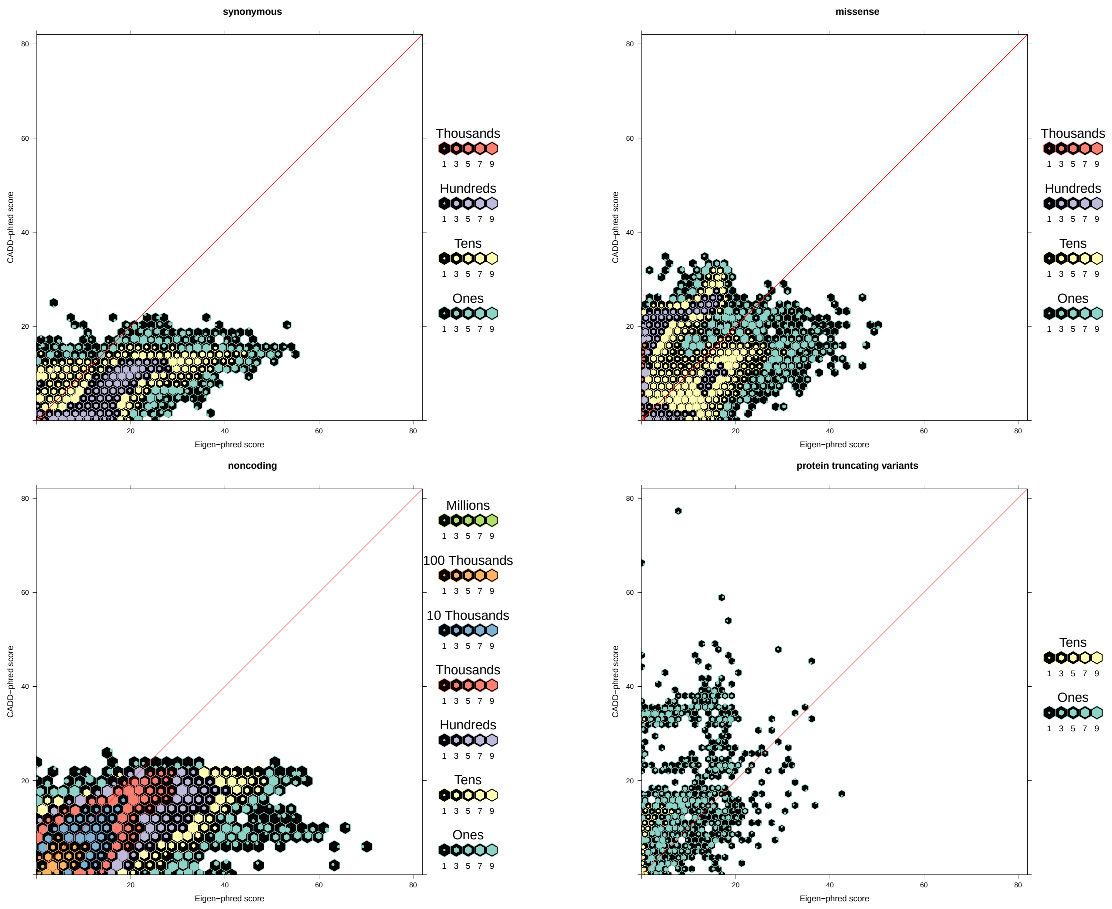

Figure S35. **Contrast between CADD and Eigen functional meta-scores on the phred-scale.** The phred scores are $-10\log_{10}$(ranks of the raw scores/total number SNPs), stratified by four variant consequence categories: missense, non-coding, synonymous, and protein truncating variants. The color and inner size of each hexagon represents the number of SNP counts.

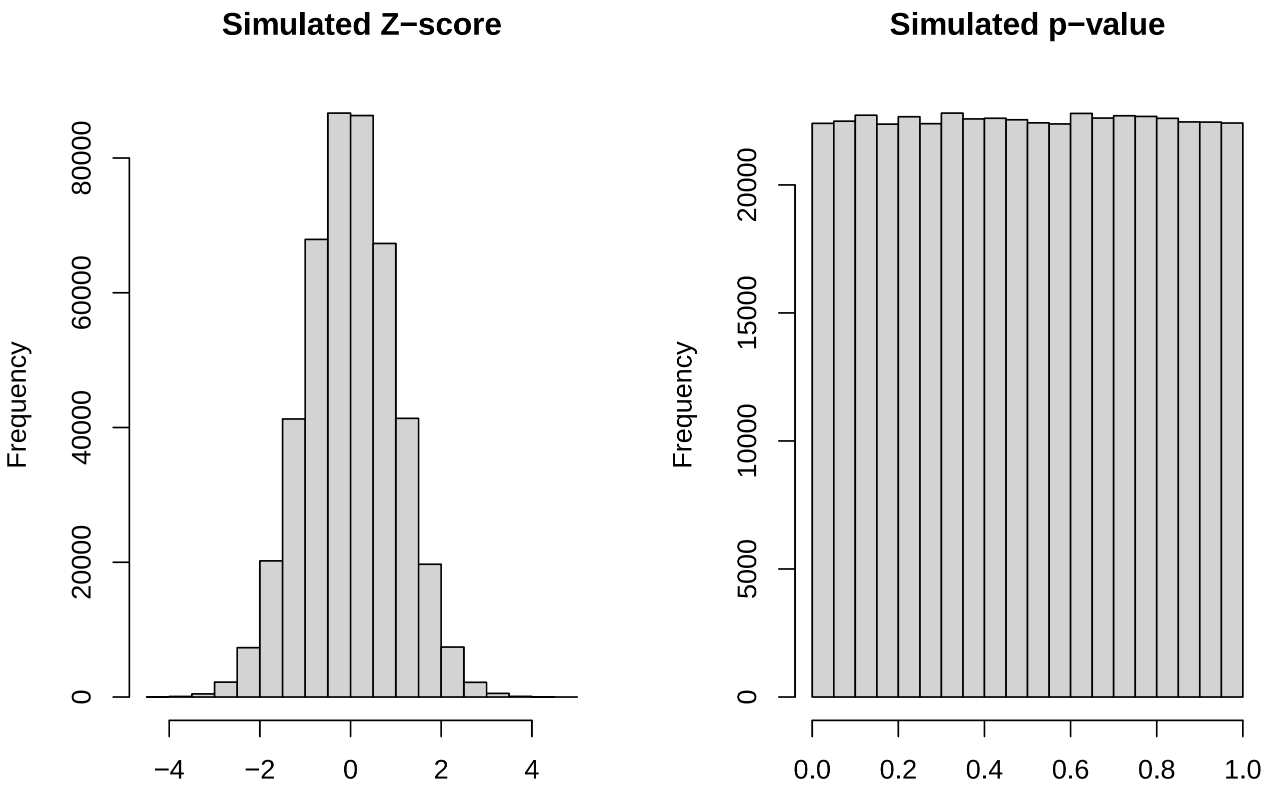

Figure S36. Histograms of GWAS Z values and p-values from the simulation study design III. A $N(0,1)$ distributed trait was simulated for 1,756 individuals from the 1000 Genomes Project, *independently* of the genotypes of 422,923 bi-allelic, autosomal and common (MAF $> 5\%$) SNPs. The GWAS summary statistics for the 422,923 SNPs were obtained by regressing the trait values of the 1,756 individuals on the additively coded genotypes. Results shown here are from one simulation run, randomly selected from a total of 50,000 simulation runs.

References

1. Quick, C. *et al.* emeraLD: rapid linkage disequilibrium estimation with massive datasets. *Bioinformatics* **35**, 164–166, DOI: 10.1093/bioinformatics/bty547 (2019).
